# Site Selectivity for the Spin States and Spin Crossover in Undecanuclear Heterometallic Cyanido-Bridged Clusters

**DOI:** 10.1021/acs.inorgchem.3c00325

**Published:** 2023-04-25

**Authors:** Le Shi, Jedrzej Kobylarczyk, Katarzyna Dziedzic-Kocurek, Jan J. Stanek, Barbara Sieklucka, Robert Podgajny

**Affiliations:** † Faculty of Chemistry, Jagiellonian University, Gronostajowa 2, 30-387 Krakow, Poland; ‡ Stoddart Institute of Molecular Science, Department of Chemistry, Zhejiang University, Hangzhou 310027, P. R. China; § Institute of Nuclear Physics PAN, Radzikowskiego 152, 31-342 Kraków, Poland; ∥ Marian Smoluchowski Institute of Physics, Jagiellonian University, Łojasiewicza 11, 30-348 Krakow, Poland

## Abstract

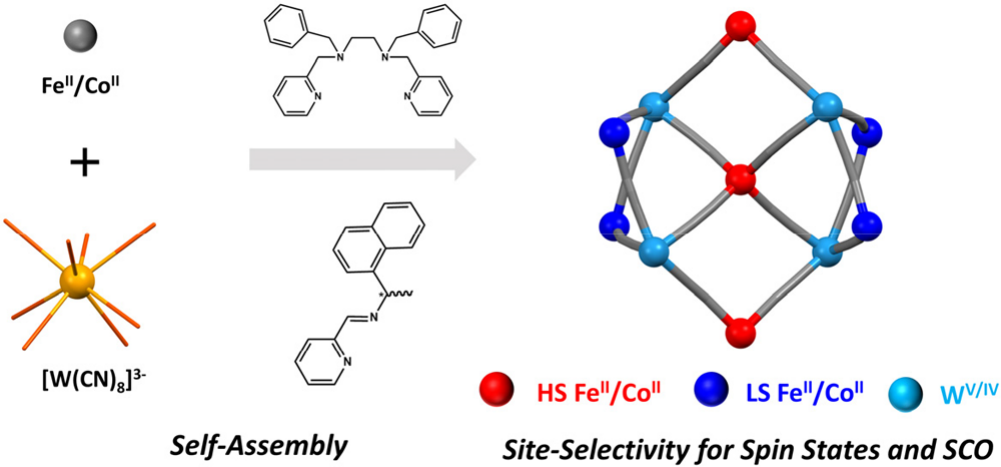

Polynuclear molecular clusters offer an opportunity to design new hierarchical switchable materials with collective properties, based on variation of the chemical composition, size, shapes, and overall building blocks organization. In this study, we rationally designed and constructed an unprecedented series of cyanido-bridged nanoclusters realizing new undecanuclear topology: Fe^II^[Fe^II^(bzbpen)]_6_[W^V^(CN)_8_]_2_[W^IV^(CN)_8_]_2_·18MeOH (**1**), Na^I^[Co^II^(bzbpen)]_6_[W^V^(CN)_8_]_3_[W^IV^(CN)_8_]·28MeOH (**2**), Na^I^[Ni^II^(bzbpen)]_6_[W^V^(CN)_8_]_3_[W^IV^(CN)_8_]·27MeOH (**3**), and Co^II^[Co^II^(*R*/*S*-pabh)_2_]_6_[W^V^(CN)_8_]_2_[W^IV^(CN)_8_]_2_·26MeOH [**4*R*** and **4*S***; bzbpen = *N*^1^,*N*^2^-dibenzyl-*N*^1^,*N*^2^-bis(pyridin-2-ylmethyl)ethane-1,2-diamine; *R*/*S*-pabh = (*R*/*S*)-*N*-(1-naphthyl)-1-(pyridin-2-yl)methanimine], of size up to 11 nm^3^, ca. 2.0 × 2.2 × 2.5 nm (**1**–**3**) and ca. 1.4 × 2.5 × 2.5 nm (**4**). **1**, **2**, and **4** exhibit site selectivity for the spin states and spin transition related to the structural speciation based on subtle exogenous and endogenous effects imposed on similar but distinguishable 3d metal-ion-coordination moieties. **1** exhibits a mid-temperature-range spin-crossover (SCO) behavior that is more advanced than the previously reported SCO clusters based on octacyanidometallates and an onset of SCO behavior close to room temperature. The latter feature is also present in **2** and **4**, which suggests the emergence of Co^II^-centered SCO not observed in previous bimetallic cyanido-bridged Co^II^–W^V/IV^ systems. In addition, reversible switching of the SCO behavior in **1** via a single-crystal-to-single-crystal transformation during desolvation was also documented.

## Abstract 2

Self-assembled undecanuclear heterometallic cyanido-bridged clusters exhibit site selectivity for spin states and SCO.

## Introduction

The rational design of new molecular clusters exhibiting multifunctionality has unceasingly attracted considerable attention owing to the demands for the miniaturization of electronic devices. In particular, clusters exhibiting spatial control over geometric electronic structures and multiple or heterofunctional groups might promote emergent properties.^1,2^ In this regard, the topology of clusters is appropriately controlled by the polyhedral features of metal-ion complexes and by the denticity (or hapticity) and connectivity of the bridging ligands, which enables cluster geometry ranging from molecular triangles^3−5^ to cages or capsules,^6^ and various superpolyhedral structures.^7^ Advanced physicochemical and functional properties can be achieved by modular modification of the overall shape, periphery zone, and metallic composition of cluster cores, which has led to many breakthroughs in the fields of catalysis,^8^ reactivity,^9^ fluorescence,^10^ host–guest recognition,^11^ and magnetism.^12,13^

One of the challenges in the field of molecular magnetism is the design of molecular spin-crossover (SCO) clusters with bistability controlled by external stimuli such as temperature, pressure, light irradiation, electric field, or guest inclusion and exchange.^14−16^ Moreover, specific arrangement of high-spin (HS) and low-spin (LS) sites in one cluster may provide more than two stable phases, being beneficial for site-selective switching and multistability,^16^ the property that provides a potential toward applications in nanotechnological devices such as memory storage units, quantum cellular automata, and molecular binary logic devices.^17,18^ Aiming at this goal, a number of SCO clusters of various nuclearities were prepared by combining the SCO-active ions with diverse bridging ligands that might cooperate in the fine-tuning of steric and electronic strain, e.g., multidentate organic ligands,^19−29^ metalloligands,^30−32^ cyanides,^33,34^ and cyanidometallates.^35−38^ Multistability and site-selective switching under thermal stimulation or photostimulation have been revealed in some dinuclear,^20^ square-like,^33−35^ grid-like,^25−27^ or metallocubane complexes^28^ as well as in the largest icosanuclear [Fe_20_] SCO clusters.^29^

As an essential contribution to the above studies, we and others applied [M(CN)_8_]^3–/4–^ (M = W, Mo, Nb, Re) building blocks of diverse structural and redox nonrigidity^39,40^ to promote thermal-induced and photoinduced charge transfer (CT) within W^V/IV^–Co^II/III^, W^V/IV^–Fe^II/III^, or Mo^IV^–Cu^II^ pairs^40^ and the SCO effect in M^V/IV^–Fe^II^ (M = W, Mo, Nb, Re) coordination networks.^41−46^ Moreover, the SCO behavior and light-induced excited-spin-state trapping effects (LIESST) were also noted at the Fe^II^ sites in discrete structures, such as a [Fe_2_M_2_] molecular rhombus^47,48^ and a [Fe_4_M_2_] octahedron,^49−51^ with the latter one featuring also site-selective double photoswitching of the Fe^II^ and Mo^IV^ sites.^50^ Particular attention was paid to the family of 15-nuclear {M_9_M′_6_} (M = Mn^II^, Fe^II^, Co^II^, Ni^II^) clusters easily affordable by crystallization from the methanol (MeOH) solution of the 3d metal salts and [M(CN)_8_].^3−60^ The composition of the core of these clusters might be appropriately adjusted to achieve HS in the ground state,^52^ slow magnetic relaxation,^53,54^ or phase transitions accompanied by CT^55−57^ and SCO.^58,59^ The SCO phenomena were systematically observed for the central [Fe(μ-NC)_6_] site owing to the favorable ligand-field stabilization energy, whereas the external [Fe(μ-NC)_3_(MeOH)_3_] sites remained in the HS state during the transition. This feature definitely precluded the observation of multistability, although multichannel bistability was achieved through a solid/solution approach.^57,59^ Furthermore, the weakly bonded MeOH molecules on the surface of a cluster allow facile ligand substitution chemistry at these specific positions to create extended clusters and polymers.^61−66^ However, until now, only the use of dedicated capping 1,4,7-trimethyl-1,4,7-triazacyclononane (Me_3_tacn) ligands allowed one to extend the SCO behavior onto the peripheral cluster areas.^64^ Thus, in a further pursuit to activate as much as possible potential SCO sites and appointing the new topologies of heterometallic clusters of modular potential, we exploited the self-assembly between [W(CN)_8_]^3–^ and Fe^II^, Co^II^, or Ni^II^ complexes with bulky N donors of various degrees of flexibility, commonly used to construct SCO complexes.^67,68^ As a result, here we present an unprecedented series of cyanido-bridged clusters realizing new undecanuclear topology: Fe^II^[Fe^II^(bzbpen)]_6_[W^V^(CN)_8_]_2_[W^IV^(CN)_8_]_2_·18MeOH (**1**), Na^I^[Co^II^(bzbpen)]_6_[W^V^(CN)_8_]_3_[W^IV^(CN)_8_]·28MeOH (**2**), Na^I^[Ni^II^(bzbpen)]_6_[W^V^(CN)_8_]_3_[W^IV^(CN)_8_]·27MeOH (**3**) (group **I**), and Co^II^[Co^II^(*R*/*S*-pabh)_2_]_6_[W^V^(CN)_8_]_2_[W^IV^(CN)_8_]_2_·26MeOH [**4*R*** and **4*S***) (group **II**); bzbpen = *N*^1^,*N*^2^-dibenzyl-*N*^1^,*N*^2^-bis(pyridin-2-ylmethyl)ethane-1,2-diamine; *R*/*S*-pabh = (*R*/*S*)-*N*-(1-naphthyl)-1-(pyridin-2-yl)methanimine]. The series was characterized by scanning electron microscopy/energy-dispersive spectroscopy (SEM/EDS), flame atomic absorption spectroscopy (FAAS), single-crystal X-ray diffraction (XRD), powder XRD (PXRD), superconducting quantum interference device (SQUID) magnetometry, UV–vis–NIR (in the solid state and in solution), IR and ^57^Fe Mössbauer spectroscopic techniques, and electrospray ionization mass spectrometry (ESI MS). We demonstrate modular features of all clusters: remarkably reproducible shape and topology, site-selective SCO activity, and corresponding site-selective spin states and bond lengths (the latter includes also SCO-inactive Ni^II^ ions), together with the overall stability and accessibility of clusters in organic media and in the gas phase. In addition, we describe the structure and SCO behavior modification associated with the single-crystal-to-single-crystal (SC–SC) transformation in **1**.

## Results and Discussion

A series of undecanuclear M_7_W_4_ clusters, Fe_7_W_4_ (**1** and **1**^**de**^), NaCo_6_W_4_ (**2**), NaNi_6_W_4_ (**3**), and (*R*/*S*)-Co_7_W_4_ (**4*R*** and **4*S*** or just **4**), were obtained by the self-assembly of 3d divalent metal-ion salts, [W(CN)_8_]^3–^ precursors and bzbpen (group **I**), or *R*/*S*-pabh ligands (group **II**) in MeOH (see the Experimental details). The general formula {M′[M^II^L_*x*_]_6_[W^V^(CN)_8_]_*y*_[W^IV^(CN)_8_]_4–*y*_}·*n*MeOH, phase purity, and composition of all compounds were confirmed by IR spectra (Figure S1), SEM/EDS, FAAS (Figures S2–S6), PXRD (Figures S7–S13), thermogravimetric analysis (TGA; Figures S14–S17), and bond-valence-sum calculations (Tables S15–S17). For the sake of simplicity, we will consider the M_7_W_4_ forms as the fundamental structural components (see further description).

### Molecular Structure

**Figure 1:**
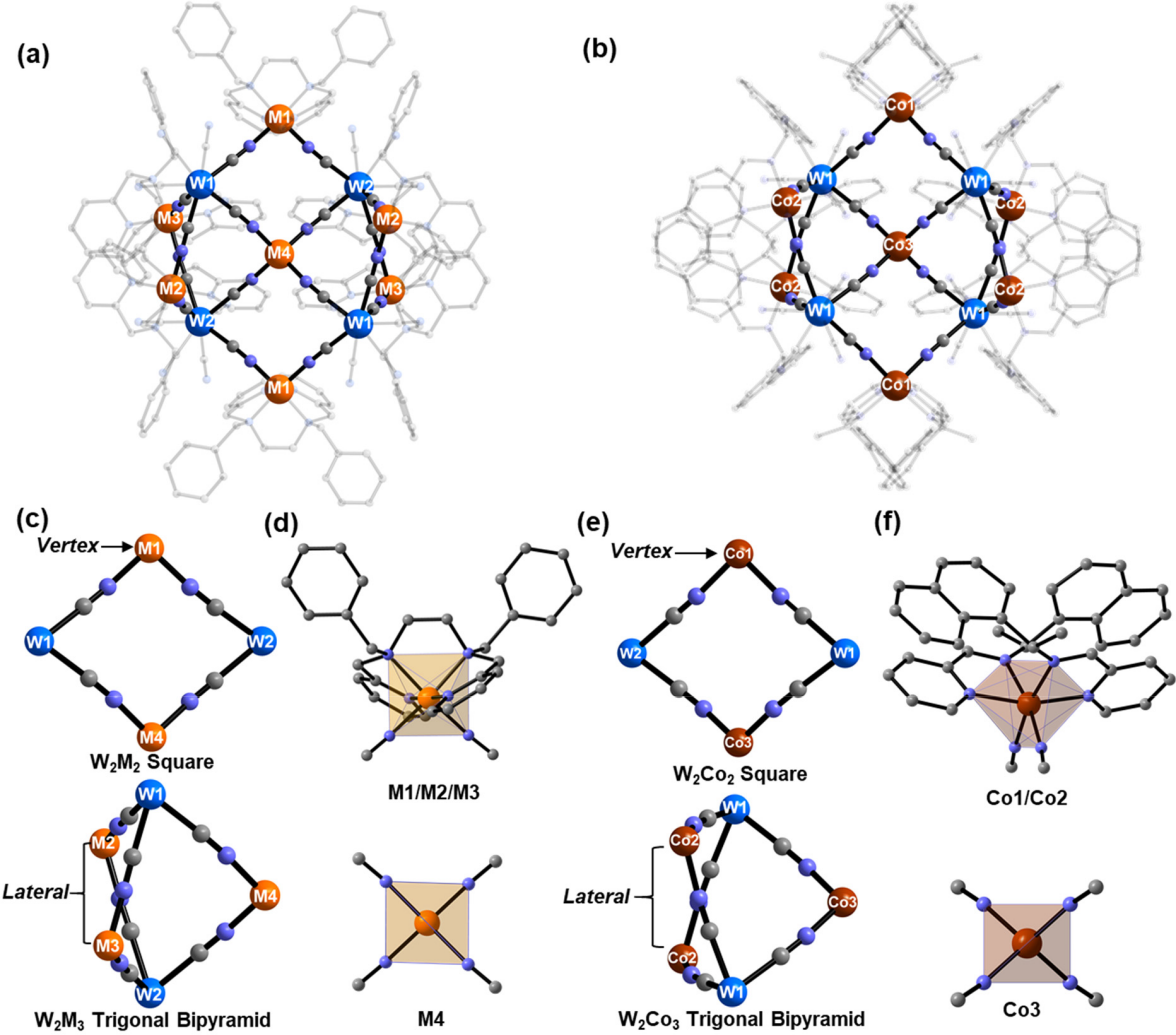
Molecular structure of group **I** (a) and group **II** (b) complexes highlighting the undecanuclear metal core, together with the positioning of *cis*-[M^II^(μ-NC)_2_(bzbpen)] moieties (topologically identical for the M1, M2, and M3 sites in **1**–**3**) (a) or of *cis*-[Co^II^(μ-NC)_2_(*R*/*S*-pabh)_2_] (topologically identical for Co1 and Co2 in **4*S*** and **4*R***) (b). Note that, in the actual crystal structure, the central M4 (M′ in general) position in **2** and **3** is Na1. (c and e) Locations of the distinguished *vertex* 3d metal-ion site in the rhombus fragments and the *lateral* 3d metal-ion sites in the trigonal-bipyramidal fragments. (d and f) N_6_ coordination spheres of 3d metal-ion moieties. Atom color code: blue, W; orange, Fe, Co, or Ni in **1**–**3**; brown, Co in **4**; gray, C; pale violet, N. MeOH molecules and H atoms were omitted for clarity.

**1**–**4** crystallize in various space groups, *C*2/*c* (isomorphous **1** and **2**), *P*2_1_/*c* (**3**), and *P*2_1_2_1_2 (**4**) (Tables S1–S3), and show the relevant different symmetry-independent units (Figures S18–S22); however, *in all of the cases*, they are composed of topologically identical cluster motifs (Figure 1). The central pseudotetrahedral [M′(μ-NC)_4_] (group **I**: M′ = M4 = Fe, **1**; Na, **2** and **3**; group **II**: M′ = Co3, **4**) moiety forms four cyanido bridges toward four neighboring [W(CN)_8_]^*n*−^ units located in the periphery of the cluster. Their supertetrahedral arrangement reproduces the connectivity of the central unit. Each [W(μ-CN)_4_(CN)_4_] unit connects with three neighboring *cis*-[M(μ-NC)_2_(bzbpen)] (group **I**) or *cis*-[Co(μ-NC)_2_(*R*/*S*-pabh)_2_] (group **II**) pseudooctahedral moieties and one tetrahedral metal center, exploiting further the formation of W–CN–M/Co linkages. In total, six such general units, topologically identical within each group, are present in the related peripheral regions of the cluster in each case. As a result, within the undecanuclear skeleton, we distinguished the rhombus M_2_W_2_ fragments involving the single peripheral *vertex* sites (marked in Figure 1 as M1 or Co1) and trigonal-bipyramidal M_3_W_2_ fragments involving the pairs of *lateral* sites (marked in Figure 1 as M2 and M3 or Co2). This assignment is important and convenient for the systematic description of the structure–property scheme of SCO and will be further used.

### Supramolecular Interactions and Contacts

**Figure 2:**
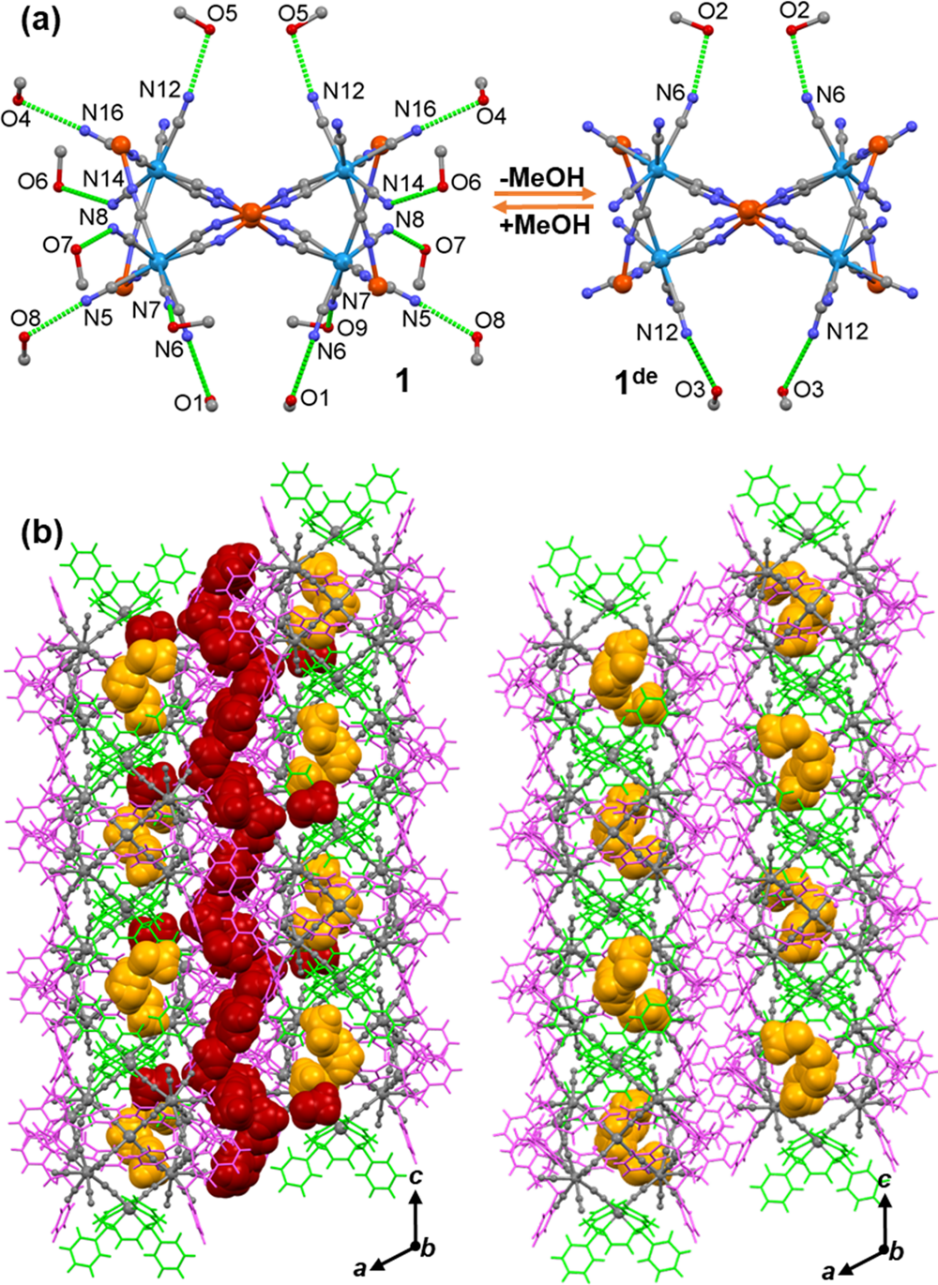
(a) Illustration of hydrogen-bond contacts between the CN^–^ groups and MeOH molecules during the SCSC transformation in **1** and **1**^**de**^. (b) Supramolecular arrangement of undecanuclear clusters of **1** and **1**^**de**^ within the *ac* plane. Color code: gray, cluster core; green, organic ligands attached to the *vertex* metal ions; pink, organic ligands attached to the *lateral* metal ions; red, MeOH molecules removed during the **1** → **1**^**de**^ process; yellow, MeOH molecules that were retained during the **1** → **1**^**de**^ process.

The crystal packing, supramolecular interactions, and contacts between the neighboring clusters in both groups are shown in Figures 2 and S23–S29. They are dominated by numerous weak hydrogen bonds involving C_phenyl_–H···N_CN_ and some perpendicular C_phenyl_–H···ring_centroid_ synthons connecting the appropriate fragments of the clusters’ periphery (Figures S23a,c and S27). Besides, crystallization MeOH molecules create a proper medium for the propagation of hydrogen bonds involving the terminal cyanido ligands and protons of phenyl rings (Figure S23b,d). In compounds **1**–**3** (group **I**), clusters are arranged in two-dimensional layers, *vertex* moieties rather loosely oriented within the layer, and *lateral* moieties protruding outside the layer to form tighter interlayer *lateral*–*lateral* contacts oriented perpendicular to the cluster’s layers, along the a* direction (Figure 2b). In close connection with the above, the MeOH molecules in **1** are unequally distributed within two distinguished solvent-accessible spaces. About two-thirds of them are located in rather narrow channels weaving along the crystallographic direction *c* and definitely form tight supramolecular contacts with the interlayer *lateral* parts of the molecular surface of the clusters arranged in the adjacent layers (Figure S25b). The intercluster separation is represented by the Fe···Fe distances of 10.605 Å (Fe2···Fe3), 10.573 Å (Fe3···Fe3), and 14.163 Å (Fe2···Fe2), respectively (Figure S26c). The remaining one-third are trapped in the intralayer cages adjacent to the *vertex* part of the cluster and show more loose interactions (Figure S25a). The shortest Fe1···Fe1 distance is 11.170 Å (Figure S26a). These “tight” and “close” contacts are also present in complexes **2** and **3** (Figures S28 and S29). Contrary to group **I**, the crystal packing of the group **II** networks exhibits rather “isotropic” three-dimensional cluster arrangement and significantly larger solvent-accessible space compared to group **I** (Figure S30). The channels of the ca. 1 nm × 1 nm square-like cross section run along the *b* direction (Figure S31). Again, we observe a similar difference in the exposition of the *vertex* and *lateralcis*-[Co(μ-NC)_2_(*R*/*S*-pabh)_2_]^2+^ and [W(CN)_8_]^3–^ moieties: the *vertex* moieties are involved in relatively less tight contacts compared to those of the *lateral* ones. The shortest intercluster separations in the vertex moieties are 16.519 and 16.591 Å for **4*S*** and **4*R***, respectively, and are slightly longer than the lateral intercluster separations, 16.306 and 16.170 Å, respectively (Figure S32).

### SC–SC Transformation **1** → **1^de^**

A single crystal of **1** was dried for 20 min in a stream of dry N_2_ at 300 K, which led to a SCSC transformation assigned as **1** → **1**^**de**^. During this process, the *C*2/*c* space group and the topology of the cluster remain intact; however, the crystal shows a significant 10% anisotropic contraction along the a* direction, perpendicular to the cluster layer, and 10% cell volume reduction (see the PXRD patterns in Figure S9). Elemental analysis and TGA indicated that 13 out of 18 MeOH molecules per one cluster in **1** (close to ^2^/_3_) were removed to leave the five MeOH molecules in **1**^**de**^ (close to ^1^/_3_) (Figure S14). This leads to modifications of the packing structure (Figures S23–S26 and Tables S5 and S6), hydrogen-bonding networks (Figure 2b), and specific bond lengths and angles (see below). The great majority of removed molecules were those located in the tight channels weaving along the *c* direction, while those located in the cages within the cluster layer underwent only minor rearrangement (Figure S25). Thus, the **1** → **1**^**de**^ process imposes more drastic changes at the *lateral* interlayer regions of the clusters compared to the *vertex* intralayer regions, leading to much closer interlayer contacts (Figure S24) compared to the intralayer contacts.

### Site-Selective Spin States and SCO

Structural data collected for HT phases (at 250 or 300 K) and for LT phases (at 100 K) revealed significant structural differences, which allowed us to differentiate the spin states along the 6-coordinated Fe^II^ and Co^II^ moieties and observe the selective thermal SCO effect at some of these sites (Tables 1 and S9–S12). On cooling of **1** from 250 to 100 K, the decrease of the average Fe1–N bond length in the *vertex* site from 2.169 to 2.025 Å is detected (Table 1; see details in Table S9), which suggests that thermal SCO behavior occurs at this site. In contrast, the average Fe–N bond lengths for the *lateral* Fe2 and Fe3 sites are slightly below 2.0 Å at both 250 K and 100 K, suggesting the LS state of these Fe^II^ centers. A similar image of the Fe–N bond lengths was noted for the desolvated form **1**^**de**^ (Table S12), for which the average Fe1–N bond length decrease from 2.144 to 2.001 Å was detected only at the Fe1 sites, on cooling from 300 to 100 K. The distinction of spin states was also confirmed by octahedral distortion bond-angle parameters Σ for both phases, ca. 50° for LS complexes and ca. 75° for HS complexes. Thus, despite the identical coordination spheres composed of two pyridines, two amines, and two isocyanides, the *vertex* Fe^II^ moieties and *lateral* Fe^II^ moieties exhibit different spin-state behavior. This indicates site selectivity at our new undecanuclear topology with respect to the spin states and SCO behavior. Finally, the Fe4–N bond lengths in the range of 2.01–2.04 Å and the angles N–Fe4–N in the general range of 98.0–129.0° (av. 109.8° in all cases) indicate unequivocally the HS tetrahedral [Fe^II^(μ-NC)_4_] moiety in the central site.^69^ The representative overlays of the high-temperature (HT) and low-temperature (LT) forms of **1** and **1**^**de**^ are shown in Figure S33.

**Table 1:** Comparison of M–N_CN_, M–N_py_, and M–N_ami_ or M–N_imi_ Bond Lengths, Average M–N Bond Lengths, and Octahedral Distortion Bond Angle Parameters Σ in the HT and LT Phases of Complexes **1**, **2**, **4*S***, and **4*R***, together with the Fe or Co Position in the Cluster and Spin-State Assignment^a^

	**1**			**2**	
	250 K	100 K		250 K	100 K
Fe1–N_CN_	2.082 ± 0.005	1.962 ± 0.013	Co1–N_CN_	2.109 ± 0.007	2.105 ± 0.012
Fe1–N_Py_	2.159 ± 0.004	2.007 ± 0.009	Co1–N_Py_	2.159 ± 0.013	2.141 ± 0.008
Fe1–N_ami_	2.262 ± 0.012	2.106 ± 0.002	Co1–N_ami_	2.255 ± 0.003	2.247 ± 0.009
**Fe1–N**_**av**_	**2.169**	**2.025**	**Co1–N**_**av**_	**2.175**	**2.164**
**Σ (deg)**	**73.08**	**53.49**	**Σ (deg)**	**77.38**	**76.43**
***vertex***	**HS**	**LS**	***vertex***	**HS**	**HS**
Fe2–N_CN_	1.912 ± 0.003	1.909 ± 0.007	Co2–N_CN_	1.973 ± 0.004	1.964 ± 0.003
Fe2–N_Py_	1.981 ± 0.006	1.978 ± 0.009	Co2–N_Py_	2.013 ± 0.007	2.012 ± 0.001
Fe2–N_ami_	2.051 ± 0.003	2.046 ± 0.004	Co2–N_ami_	2.096 ± 0.007	2.076 ± 0.006
**Fe2–N**_**av**_	**1.982**	**1.978**	**Co2–N**_**av**_	**2.027**	**2.017**
**Σ (deg)**	**48.44**	**47.44**	**Σ (deg)**	**50.49**	**49.23**
***lateral***	**LS**	**LS**	***lateral***	**LS**	**LS**
Fe3–N_CN_	1.915 ± 0.010	1.914 ± 0.003	Co3–N_CN_	1.929 ± 0.003	1.917 ± 0.005
Fe3–N_Py_	1.982 ± 0.009	1.978 ± 0.008	Co3–N_Py_	1.968 ± 0.006	1.980 ± 0.002
Fe3–N_ami_	2.053 ± 0.004	2.055 ± 0.007	Co3–N_ami_	2.036 ± 0.002	2.024 ± 0.006
**Fe3–N**_**av**_	**1.984**	**1.983**	**Co3–N**_**av**_	**1.977**	**1.974**
**Σ (deg)**	**52.14**	**49.94**	**Σ (deg)**	**47.22**	**45.34**
***lateral***	**LS**	**LS**	***lateral***	**LS**	**LS**

	**4*S***			**4*R***	
	250 K	100 K		250 K	100 K
Co1–N_CN_	2.127	2.131	Co1–N_CN_	2.121	2.129
Co1–N_Py_	2.111	2.092	Co1–N_Py_	2.090	2.116
Co1–N_imi_	2.225	2.213	Co1–N_imi_	2.244	2.207
**Co1–N**_**av**_	**2.154**	**2.145**	**Co1–N**_**av**_	**2.152**	**2.151**
**Σ (deg)**	**85.32**	**79.64**	**Σ (deg)**	**79.93**	**80.45**
***vertex***	**HS**	**HS**	***vertex***	**HS**	**HS**
Co2–N_CN_	1.974 ± 0.008	1.977 ± 0.002	Co2–N_CN_	1.984 ± 0.002	1.972 ± 0.005
Co2–N_Py_	2.032 ± 0.01	2.044 ± 0.004	Co2–N_Py_	2.046 ± 0.001	2.021 ± 0.001
Co2–N_imi_	2.101 ± 0.001	2.072 ± 0.013	Co2–N_imi_	2.072 ± 0.001	2.046 ± 0.004
**Co2–N**_**av**_	**2.036**	**2.031**	**Co2–N**_**av**_	**2.034**	**2.013**
**Σ**^b^**(deg)**	**57.94**	**56.33**	**Σ (deg)**	**58.76**	**62.55**
***lateral***	**LS**	**LS**	***lateral***	**LS**	**LS**

a The assignment for **1** is also representative for **1**^**de**^ (Table S12).

b Σ is the sum of the deviations of the 12 cis angles of the MN_6_ octahedron from 90°.

**Figure 3:**
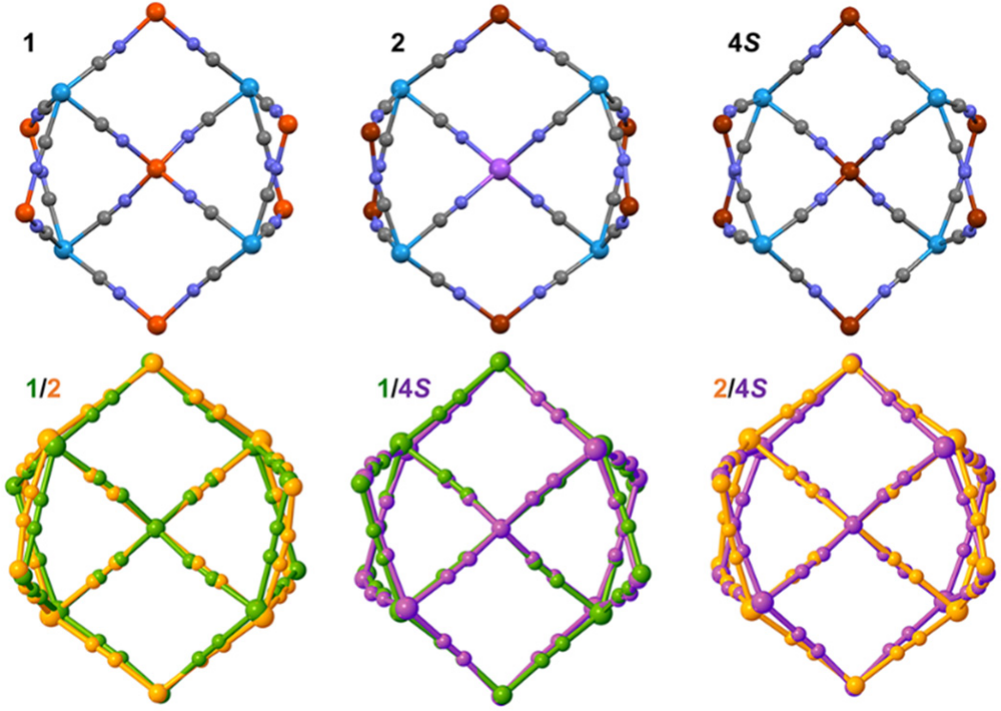
Superimposed structures of **1**/**2**, **1**/**4**, and **2**/**4** illustrating spatial convergence between the related cyanido-bridged skeletons along the same undecanuclear topology. The chelating organic ligands, terminal cyanido ligands, solvent molecules, and H atoms were omitted for clarity.

The above coordination site speciation is also reflected by the relevant structural parameters of the Co analogues, **2** in group **I**, and **4*S*** and **4*R*** in group **II** (Tables 1 and S10 and S11). However, the average Co1–N bond lengths in **2** indicate the presence of HS Co^II^ complexes in the *vertex* sites, both at the HT phase (2.175 Å) and at the LT phase (2.164 Å), whereas the average Co–N bond lengths at the *lateral* sites approach 2.02 Å (Co2) or 1.98 Å (Co3), which suggest the occurrence of LS Co^II^ complexes. A very similar scenario was observed for the pair **4*R*** and **4*S***, despite the alternative coordination sphere of two imines, two pyridines, and two isocyanides: the average Co–N bond lengths at the *vertex* Co1 site are close to 2.15 Å, while at the *lateral* Co2 site, they are close to 2.02 or 2.03 Å at both examined temperatures. The values of Σ confirm the above spin-state distribution. At the central M′ sites, **4*R*** and **4*S*** reveal the average Co–N_isocyanide_ bond lengths of 1.93–1.95 Å, in agreement with the values observed previously for tetrahedral HS [Co(μ-NC)_4_] moieties,^70^ whereas **2** accommodates Na^+^ cations with reasonable Na–N_cyanide_ distances of ca. 2.27 and 2.30 Å,^71^ which leads to a slight expansion of the cluster core along the W–CN–Na direction compared to complexes **1** and **4*R***/**4*S*** (Figure 3). The absence of SCO in the temperature range 100–250 K in **2** and **4** might be understood in terms of the hard-to-overcome steric demands associated with the significant Jahn–Teller distortion expected for the LS state. This is in line with the fact that a pure Co^II^-centered SCO was not realized in the bimetallic cyanido-bridged Co^II^–W^V/IV^ systems to date, although some systems revealed switchable CT-induced spin transition phenomena involving the ^HS^Co^II^W^V^ ⇄ ^LS^Co^III^W^IV^ equilibrium in the solid state.^72−76^

The spin-state site selectivity disclosed above is independent of the metal ions (Fe^II^ or Co^II^) and, to a reasonable extent, of the ligand field. Therefore, SCO transitions in the case of Fe^II^ compounds most likely stem from geometric constraints inherent in the undecanuclear topology and from the bulk ligand steric effect. The *vertex* 3d metal-ion moieties in rhombus W_2_M_2_ fragments possess a relatively large degree of freedom, which is conducive to stabilization of the HS state. Moreover, the organic ligands coordinated at those sites as well as the terminal cyanido ligands located nearby are definitely exposed toward the less crowded and less rigid solvent-accessible space, which provides the appropriate molecular arrangement supportive for the expanded HS complexes and for the rearrangements accompanying the transition. On the contrary, metal ions located in the *lateral* sites are more confined and thus conditioned to remain at the LS state, not only by the rigidity of the trigonal-bipyramidal geometry but also by bulk ligand hindrance due to more intense intercluster interactions. The impact of the geometric constraints on the HS and LS states in **1**, **2**, and **4** can be further supported by the structural data for Ni analogue **3** within group **I**. All peripheral octahedral Ni^II^ ions possess the t_2g_^6^e_g_^2^ configuration, to show slightly longer Ni–N bond lengths at the *vertex* sites, 2.12–2.13 Å, compared to those located at the *lateral* sites, 2.09–2.10 Å, both at 250 K (Table S13).

### Magnetic Properties

**Figure 4:**
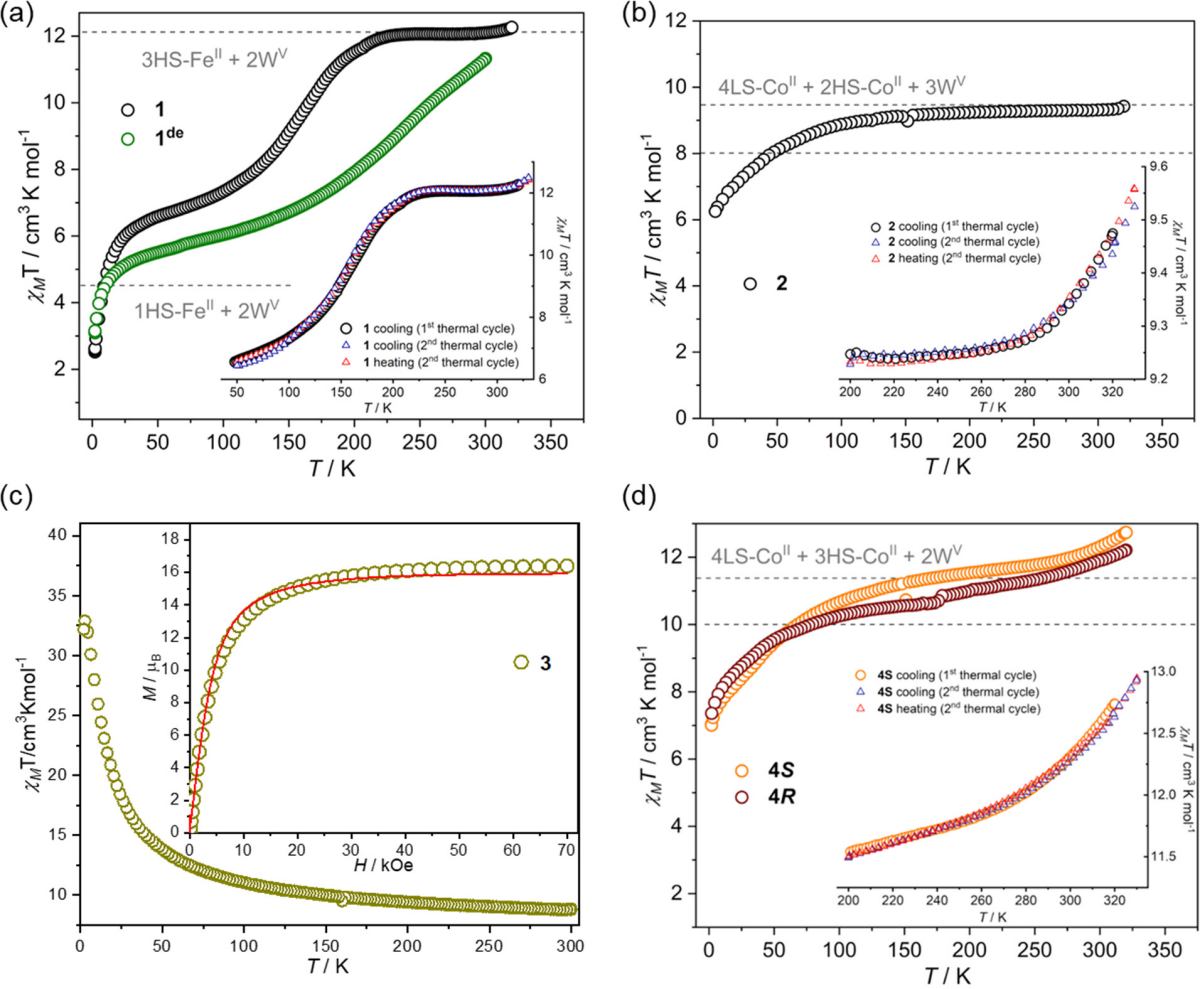
Magnetic χ_M_*T*(*T*) curves for **1** and **1**^**de**^ (a), **2** (b), **3** (c), **4*R*** and **4*S*** (d). The gray dashed lines show estimated χ_M_*T* values or their ranges for different spin-state populations of the undecanuclear clusters. Insets: (a, b, and d) χ_M_*T*(*T*) data in the extended range up to 330 K collected in the second cycle; (c) *M*(*H*) plot at 2.0 K (dark-yellow circles) compared with the Brillouin functions for *S* = ^15^/_2_ and *g*_av_ = 1.99 (red line).

The χ_M_*T*(*T*) products for compounds **1**–**4** in the range 330–2 K suggest that **1**, **1**^**de**^, **2**, and **4** are SCO complexes, whereas the Ni compound **3** is an SCO-inactive HS molecule with *S*_gr_ = ^15^/_2_ (Figures 4 and S34 and Table S14). For **1**, the χ_M_*T* value at 330 K is 12.44 cm^3^ K mol^–1^ and gradually decreases to 12.09 cm^3^ K mol^–1^ at 300 K, in good agreement with the value of 12.14 cm^3^ K mol^–1^ predicted for magnetically uncorrelated three HS Fe^II^ ions of *S* = 2 and *g*_Fe_ = 2.25 and two paramagnetic [W(CN)_8_]^3–^ units of *S* = ^1^/_2_ and *g*_W_ = 2.0, accompanied by diamagnetic moieties provided by four LS Fe^II^ complexes and two [W(CN)_8_]^4–^ complexes, in agreement with the structural data. The decrease of χ_M_*T* in the 330–300 K temperature range is attributed to the onset of spin transition of the *lateralcis*-[Fe(μ-NC)_2_(bzbpen)] moieties occurring most probably in the higher temperatures (Figures 4a and S34a). Upon further cooling, the χ_M_*T* value remains constant until 250 K and then notably decreases, tending toward a shallow *plateau* represented by values 7.34 cm^3^ K mol^–1^ at 100 K and 6.58 cm^3^ K mol^–1^ in 50 K. The decrease in χ_M_*T* of ca. 4.75–5.51 cm^3^ K mol^–1^ is evidently smaller than 7.25–7.93 cm^3^ K mol^–1^ expected for a complete transition HS → LS of two Fe^II^ complexes, assuming *g*_Fe_ in the range 2.2–2.3. The above decline indicates a partial transition occurring on ca. 60–75% SCO-active Fe^II^ centers and thus should be assigned to the incomplete SCO process occurring at the *vertexcis*-[Fe(μ-NC)_2_(bzbpen)]^2+^ moieties. While the 100 K temperature point was given as a reference, the onset of the mid-temperature LS → HS transition might be located slightly above this point. The exact indication of this onset is blurred by the contribution from the zero-field-splitting (ZFS) properties expected for the HS Fe^II^ ions.^46−51,58^**1**^**de**^ shows a similar magnetic behavior, but it is a bit less advanced in the range of 50–300 K. The χ_M_*T* value of 11.46 cm^3^ K mol^–1^ at 300 K is very close to the values predicted for three HS Fe^II^ ions and four LS Fe^II^ ions. As the temperature is lowered, the χ_M_*T* curve shows a gradual decrease to 6.03 cm^3^ K mol^–1^ at 100 K and 5.53 cm^3^ K mol^–1^ at 50 K. The decrease in the amplitude of χ_M_*T* of ca. 5.03–5.93 cm^3^ K mol^–1^ indicates at least 64–82% SCO completion. The overall course of the χ_M_*T*(*T*) curves below 100 K indicates the expected essential contribution of the ZFS from the remaining Fe^II^ HS centers and weak antiferromagnetic (AF) interactions along the W^V^–CN–Fe^II^ linkages.^46,48,55,59,64^ In addition, the *M*(*H*) curves at 2 K for both complexes show relatively large magnetization values of 5.39 and 4.79 μ_B_ for **1** and **1**^**de**^, respectively, further supporting the presence of the remaining HS Fe^II^ complexes at low temperatures (see details in Figure S34). Because the preparation means for sample **1**^**de**^ (foil bag) preclude reliable examination of its magnetic properties above 300 K, we are not able to judge the development of the SCO properties at these conditions.

For structure–property relationship for **1** and **1**^**de**^, careful inspection of the structures provides some clues. (i) The spin-transition temperature (*T*_1/2_ values) for **1** (*T*_1/2_ = 157 K) is significantly lower than that for **1**^**de**^ (*T*_1/2_ = 224 K), which indicates stronger stabilization of the LS states in the latter phase. This might be correlated with the increased intercluster steric crowding because of the loss of crystallization solvents in the channel space favoring LS states, thus raising *T*_1/2_. (ii) No hysteretic effect was observed in any of the crystals; however. **1** exhibited slightly better cooperativity than **1**^**de**^. It is reasonable to attribute weak cooperativity to the domination of C_phenyl_–H···N_CN_ and perpendicular C_phenyl_–H···ring_centroid_ contacts, while the presence of crystallization MeOH molecules might just tune that property to some extent; actually, in our case, the increased contribution of intercluster noncovalent interactions involving MeOH as a reasonable mediator of hydrogen bonds visibly enhances the cooperativity.

The χ_M_*T*(*T*) curve for **2** at the HT region presents a behavior similar to that of **1**. The χ_M_T values gradually decreased from 9.56 cm^3^ K mol^–1^ at 330 K to 9.31 cm^3^ K mol^–1^ at 300 K, which approaches reasonably the range of 8.03–9.46 cm^3^ K mol^–1^, predicted for the presence of two HS *cis*-[Co(μ-NC)_2_(bzbpen)_2_] with *S*_HS-Co_ = ^3^/_2_ and *g*_HS-Co_ in the range 2.4–2.7, four LS *cis*-[Co(μ-NC)_2_(bzbpen)_2_] with *S*_LS-Co_ = ^1^/_2_ and *g*_LS-Co_ = 2.0, and three [W(CN)_8_]^3–^ moieties with *S*_W_ = ^1^/_2_ and *g*_W_ = 2.0, together with one diamagnetic [W(CN)_8_]^4–^ moiety and one Na^+^ cation. Below 300 K, χ_M_*T*(*T*) does not significantly decrease until 50 K and then diminishes more rapidly to reach a value of 6.25 at 2.0 K. For **4*S*** and **4*R***, the χ_M_*T* values are 12.93 cm^3^ K mol^–1^ at 330 K and 12.22 cm^3^ K mol^–1^ at 300 K, respectively. Then, the curves gradually decrease to 11.78 and 11.34 cm^3^ K mol^–1^ at 250 K, respectively, approaching reasonably the range of 9.92–11.35 cm^3^ K mol^–1^, predicted for the presence of one tetrahedral [Co(μ-NC)_4_] moiety with *S*_Td-Co_ = ^3^/_2_ and *g*_Td-Co_ = 2.2, two HS *cis*-[Co(μ-NC)_2_(*R*/*S*-pabh)_2_] with *S*_HS-Co_ = ^3^/_2_ and *g*_HS-Co_ in the range 2.4–2.7, four LS *cis*-[Co(μ-NC)_2_(*R*/*S*-pabh)_2_] with *S*_LS-Co_ = ^1^/_2_ and *g*_LS-Co_ = 2.0, two [W(CN)_8_]^3–^ ions with *S*_W_ = ^1^/_2_ and *g*_W_ = 2.0, and two diamagnetic [W(CN)_8_]^4–^ moieties. As the temperature is lowered, the χ_M_*T* values for both complexes decrease smoothly in the HT regime and then decrease rapidly below 50 K to reach the values of 7.01 and 7.36 cm^3^ K mol^–1^ for **4*S*** and **4*R***, respectively. In both **2** and **4**, the HT behavior may indicate the onset of a SCO transition (Figures 4b,d and S34b,c) most probably centered on the *lateral* LS *cis*-[Co(μ-NC)_2_(bzbpen)_2_] and *cis*-[Co(μ-NC)_2_(*R*/*S*-pabh)_2_] moieties, respectively. The smooth decrease of the χ_M_*T* curves at the HT and mid-temperature regions should be interpreted in terms of the SOC/ZFS properties of octahedral Co^II^ complexes,^77^ whereas at the LT region, the component of magnetic exchange coupling within W^V^–CN–Co^II^ linkages might be important.^72,78−82^ However, the single-ion properties dominate the magnetic behavior of the clusters. All presented magnetic data for **2** and **4** are in good agreement with the relevant structural data.

The χ_M_*T* value of **3** at 300 K is 8.70 cm^3^ K mol^–1^, which is close to the predicted value of 8.39 cm^3^ K mol^–1^ calculated for the uncoupled six HS Ni^II^ ions of *S* = 1 and *g*_Ni_ = 2.2 and three paramagnetic [W(CN)_8_]^3–^ units of *S* = ^1^/_2_ and *g*_W_ = 2.0 (Figure 4c). Upon cooling, χ_M_*T* gradually increases up to a maximum of 33.21 cm^3^ K mol^–1^ at 4.0 K. This can be attributed to the ferromagnetic coupling along the W^V^–CN–Ni^II^ linkages, in line with the cyanido-bridged W^V^Ni^II^ complexes reported previously.^53,62,83−85^ The drop of the signal below 4.0 K is due to the combined effects of the single-ion anisotropy on Ni^II^ ions and possible intercluster AF interactions. The maximal value of χ_M_*T* may be compared with the 31.56 cm^3^ K mol^–1^ expected for an exchange-coupled cluster with a *S* = ^15^/_2_ ground state spin value and *g*_av_ = 1.99. The ferromagnetic character is further supported by the field dependence of magnetization gathered at *T* = 2.0 K. It shows a saturation value of 16.38 μ_B_ at 70 kOe, which is slightly higher than the expected values of 16.2 μ_B_ for the parallel alignment of all magnetic moments. No alternating-current signal was detected. Based on the Ni–N≡C angle range of 161–173° (av. value 167.4°; see details in Table S18) within the coordination skeleton of **3**, a rough estimation of *J* ∼ +10 cm^–1^ (2*J* formalism) might be inferred.^53,62,83,84^

We definitely exclude the loss of solvent and sample degradation in **1**–**4** expected and frequently observed while approaching the boiling point of the solvent used, in case the sample is not protected properly. This is supported by perfect reversibility of the curves during applied thermal cycles, thanks to the effective protection in sealed glass tubes.

### ^57^Fe Mössbauer Spectroscopy

**Figure 5:**
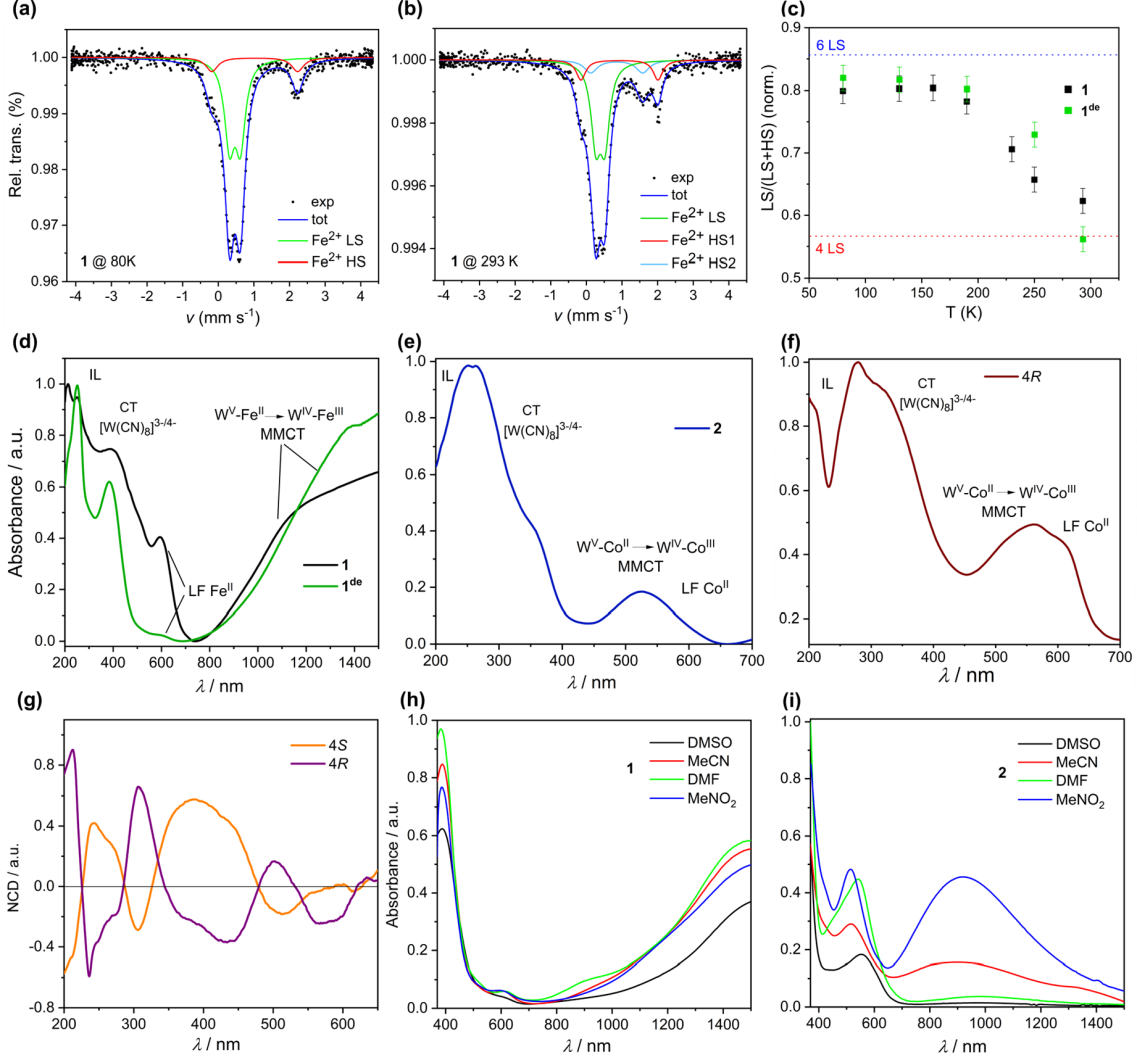
Representative ^57^Fe Mössbauer spectra of **1** in *T* = 80 K (a) and *T* = 293 K (b), together with the best fits. (c) Course of the SCO transitions illustrated by the amount of LS complexes for **1** (black-filled squares) and **1**^**de**^ (green-filled squares) derived from the fits in various temperatures. The dashed lines indicate the limiting amount of LS complexes, blue is for the LT phase, and red is for the HT phase. UV–vis–NIR spectroscopic studies on undecanuclear clusters: diffuse-reflectance UV–vis–NIR spectra of **1** and **1**^**de**^ at room temperature (d); diffuse-reflectance UV–vis–NIR spectra of **2** (e) and **4*S*** and **4*R*** (f) in the 200–700 nm range (see details in the SI); (g) NCD spectra for **4*R*** and **4*S***; UV–vis–NIR spectra of **1** (h) and **2** (i) (0.1 mmol/L) in different solvents at room temperature.

The above overall observations for **1** and **1**^**de**^ were further confirmed by ^57^Fe Mössbauer spectroscopy studies (Figure 5a–c; see details in the Supporting Information, SI). The spectra measured at 80 K (Figure 5a) were in both cases reasonably reproduced as a sum of a weakly split doublet assignable to the LS state (green fit component) and a strongly split doublet assigned to the HS state (red fit component). With increasing temperature, first no significant changes are noted; then above ca. 150 K, the LS contributions begin to decrease (Figure 5b), which is followed by the visible systematic emergence of the third moderately split component HS2 (pale-blue component; Figures 5b and S35 and S36 and Tables S19 and S20). Such behavior beyond a doubt indicates the occurrence of spin transition, in line with the structural and magnetic data. The percentage contributions of LS components in the LT phase are 80% for **1** and 82% for **1**^**de**^, which might be recalculated into 5.6 (**1**) and 5.74 (**1**^**de**^), representing the number of LS complexes per seven Fe^II^ centers in one cluster in these conditions. The resulting HS/LS ratios in the LT phase, 1.4/5.6 (**1**) and 1.26/5.74 (**1**^**de**^), indicate some excess over the ideal ratio 1/6 expected for one tetrahedral HS [Fe^II^(μ-NC)_4_] moiety and six LS [Fe(bzbpen)(μ-NC)_2_] moieties; however, it indeed conforms with some excess of HS complexes (over one per cluster) deduced from the LT magnetic data. As the result of spin transition, at room temperature, the percentage contributions of LS are decreased to 63% (**1**) and 56% (**1**^**de**^), which might be recalculated as 4.4 (**1**) and 3.92 (**1**^**de**^). These values result in the HS/LS ratios being very close to the ideal 3/4 (per one cluster) expected for one tetrahedral HS [Fe^II^(μ-NC)_4_] moiety, two HS [Fe(bzbpen)(μ-NC)_2_] moieties assigned to the *vertex* sites of the cluster, and four LS [Fe(bzbpen)(μ-NC)_2_] moieties assigned to the *lateral* sites of the cluster, based on the structural data. It is important to note that the Mössbauer data decently reproduce the difference in the χ_M_*T*(*T*) curves for **1** and **1**^**de**^. For **1**, the transition occurs in a more pronounced manner and visibly attains well-resolved saturation at room temperature. In contrast, for **1**^**de**^, the decrease of the LS contribution occurs more slowly, and saturation is definitely not achieved in these conditions, which clearly confirms better stabilization of the *vertex* HS complexes in **1**, illustrated by *T*_1/2_(**1**) definitely being lower than *T*_1/2_(**1**^**de**^) in the Mössbauer data. It must be, however, stressed that in our case the Mössbauer data cannot be used for an exact determination of the onset of the LS → HS transition temperature (like the SQUID data can) in the mid-temperature range. This is due to the relatively small “active space” for spin-state change, 1.5–2 per seven Fe centers per whole molecule, keeping in mind the limited accuracy of this technique in tandem with sample preparation: the limited sample mass and the requirement to use protecting means. Nevertheless, we definitely excluded sample degradation during both measurements.

### Optical Spectra and Stability of Clusters

The solid samples of complexes **1**–**4** were further examined by UV–vis–NIR spectroscopy, and the expected spectral features were identified (Figures 5d–f and S37–S40). The strong absorption in the 200–400 nm range is assigned to the sum of the CT bands of the [W(CN)_8_]^*n*−^ moieties,^86^ CT absorption of the 3d metal-ion complex moiety, and intraligand transitions of the involved ligands.^62,87^ In the lower-energy range, the ligand-field bands^62,78^ of the complex moieties and metal-to-metal CT transitions M^II^–W^V^ → M^III^–W^IV^ (**1**, **2**, and **4**)^55,72,78,87^ along the M–NC–W linkages were recognized. The natural circular dichroism (NCD) spectra of **4*R*** and **4*S*** are complementary to each other in the examined 200–700 nm range, which confirms the enantiopurity of both phases and indicates nonzero optical activity of the clusters within all related absorption bands (Figure 5g). The solution spectra in acetonitrile (MeCN), MeNO_2_ (**1**–**3**), or *N*,*N*-dimethylformamide (DMF; **4**) present compositions of bands similar to those of the solid-state spectra (Figures 5h,i and S41 and S42), which suggests that the described polynuclear complexes retain their identity in the solution state, despite **4*R*** and **4*S*** showing a gradual decomposition in DMF (Figure S42). The stability of the clusters in solution is also supported by ESI MS (Figures S43 and S44). In particular, the MS spectrum of **1** in MeCN shows the peak-set patterns assigned to the singly reduced {Fe_7_(bzbpen)_6_[W(CN)_8_]_4_]^−^ and doubly reduced {Fe_7_(bzbpen)_6_[W(CN)_8_]_4_]^2–^ complete undecanuclear clusters, although the strongest peak-set pattern is definitely assignable to the {Fe_2_(bzbpen)_2_[W(CN)_8_]_2_]}^2–^ motif.

## Concluding Remarks

**Figure 6:**
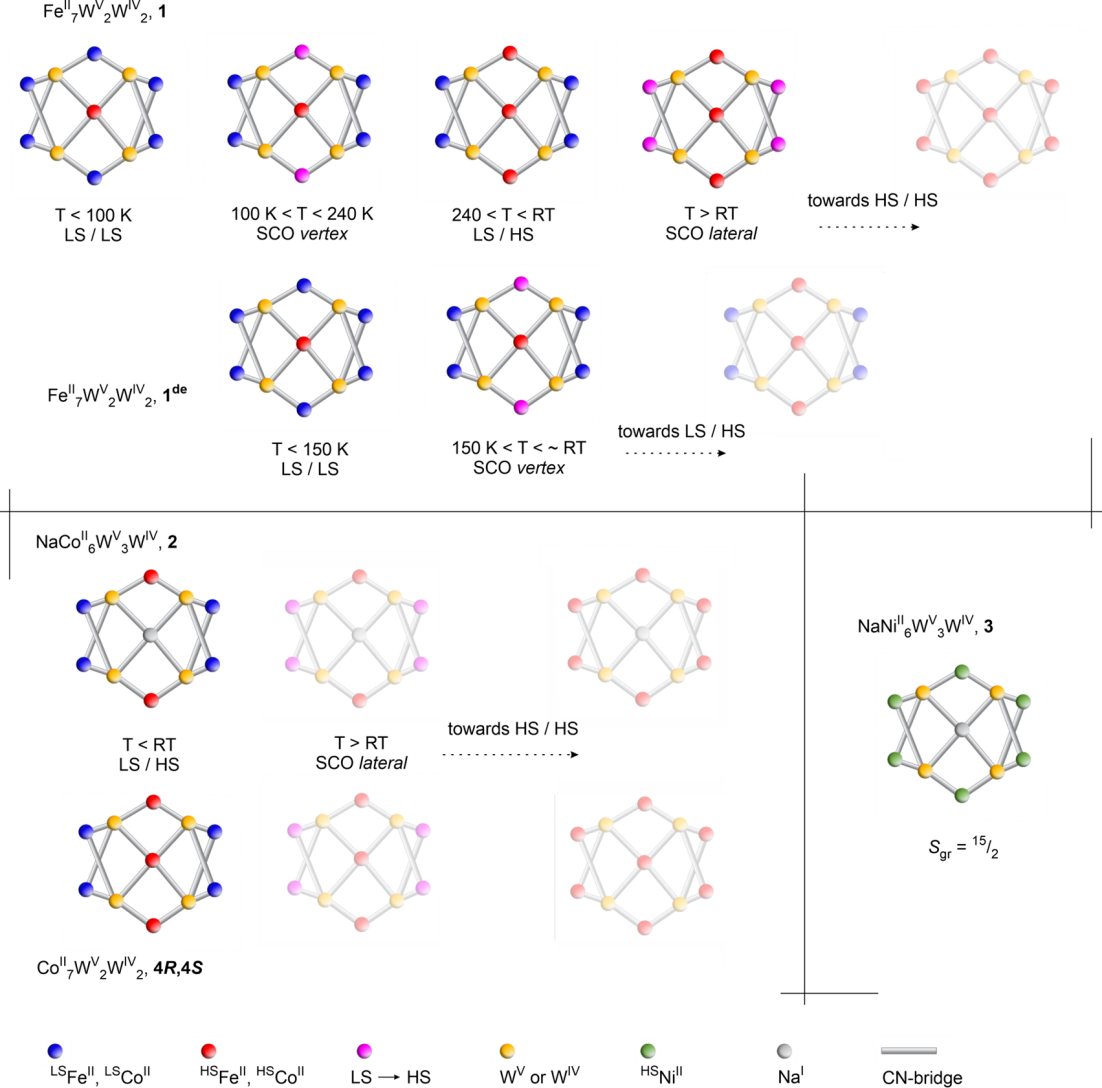
Summary of the properties in the **1**–**4** clusters family. (Top) Fe^II^_7_W^V^_2_W^IV^_2_ (**1**) is a double-step SCO compound with distinct spin transition occurring at the *vertex* [Fe(bzbpen)(μ-NC)_2_] moieties in the temperature range 100–240 K and the onset of another spin transition at the *lateral* [Fe(bzbpen)(μ-NC)_2_] positions occurring just above room temperature. Its partially desolvated form **1**^**de**^ shows only one SCO transition step within the accessible *T* range occurring at the *vertex* [Fe(bzbpen)(μ-NC)_2_] moieties, starting from 150 K and expected to be finished just above room temperature. (Bottom, left panel) NaCo^II^_6_W^V^_3_W^IV^ (**2**) and Co^II^_7_W^V^_2_W^IV^_2_ (**4*R*** and **4*S***) exhibit distinct HS forms at the *vertex* [Co(bzbpen)(μ-NC)_2_] or [Co(pabh)_2_(μ-NC)_2_] moieties and LS forms at the *lateral* moieties; the onset of spin transition at the *lateral* [Fe(bzbpen)(μ-NC)_2_] is ongoing close to room temperature. (Bottom, right panel) NaNi^II^_6_W^V^_3_W^IV^ (**3**) is a paramagnet composed of HS clusters of the spin in the ground state *S*_GS_ = ^15^/_2_. However, even this compound reveals slightly shorter Ni–N bond lengths at the *lateral* sites than at the *vertex* sites, in line with the trends observed in **1**, **2**, **4*R***, and **4*S***. The forms expected above room temperature are covered with a white semitranslucent mask.

The presented series of undecanuclear M_7_W_4_ (M = Fe, Co) and NaM_6_W_4_ (M = Co, Ni) clusters provides novel, versatile modules for the generation of diverse magnetic properties. The NaNi_6_W_4_ complex **3** exhibits HS in the ground state, whereas the Fe and Co complexes **1**, **1**^**de**^, **2**, **4*R***, and **4*S*** reveal various scenarios of site-selective SCO and spin state at specific metal sites, which are summarized in Figure 6. Our results provide an important contribution toward knowledge on multistable molecular materials and the development of their acquisition. In particular, the speciation of sites important from the viewpoint of switchable behavior was achieved, exploiting *the same* type of coordination environment. As the crucial factors determining the spin-state distribution and SCO properties, we indicated the geometric constraints imposed on various regions of the cluster core (as the endogenous, inner factor) and intermolecular noncovalent interaction schemes in the related areas of the crystal architectures (as the exogenous, outer factor). As the novelty to the field, **2** and **4** represent the emergence of Co^II^-centered SCO compounds within the Co-[M(CN)_8_] family, not observed in previous bimetallic cyanido-bridged Co^II^–W^V/IV^ systems, whereas **1** exhibits the first well-resolved two-step SCO system among the Fe-[W(CN)_8_] clusters. Moreover, these clusters unquestionably belong to the largest molecules known to exhibit site-selective spin state and SCO, with the dimensions of ca. 2.0 × 2.2 × 2.5 nm (**1**–**3**) and ca. 1.4 × 2.5 × 2.5 nm (**4*R***/**4*S***), considering the distance between the most remote atoms along three cluster axes. Various metallic core compositions embedded within the same topological scaffold and the related SCO scenarios open a promising route for molecular design toward advanced cocrystal salts involving switchable polynuclear clusters^88,89^ and molecular hierarchical systems such as heterotrimetallic solid solutions^55,56,59,60^ or core–shell composites;^90^ the solubility and stability of these forms in various organic solvents might be one of the prerequisites in further research. Moreover, the research on SCO transitions in multisite clusters might provide better characteristics (e.g., larger cooperativity, SCO site speciation, multistep transitions, etc.).

## Supporting Information Available

Detailed description of the used materials, synthetic procedures, physical techniques, crystal structure determination, and structural data presentation and additional figures and tables (PDF)
