# Supplementary material for: Site Selectivity for the Spin States and Spin Crossover in Undecanuclear Heterometallic Cyanido-Bridged Clusters
Source: Inorg Chem. 2023 Apr 25;62(18):7032–44. doi: 10.1021/acs.inorgchem.3c00325 (PMC10170501; doi:10.1021/acs.inorgchem.3c00325)
Supplement: Supplementary file 1 — ic3c00325_si_001.pdf [file ic3c00325_si_001.pdf]

Site-Selectivity for the Spin States and Spin Crossover in Undecanuclear Heterometallic Cyanido-Bridged Clusters

Le Shi[a,b], Jedrzej Kobylarczyk[a,c], Katarzyna Dziedzic-Kocurek[d], Jan J. Stanek[d], Barbara Sieklucka[a] and Robert Podgajny[a]**

[a] Faculty of Chemistry, Jagiellonian University, Gronostajowa 2, 30-387 Krakow, Poland.
[b]Stoddart Institute of Molecular Science, Department of Chemistry, Zhejiang University, Hangzhou 310027, P. R. China
[c] Institute of Nuclear Physics PAN, Radzikowskiego 152, 31-342 Kraków, Poland
[d] Marian Smoluchowski Institute of Physics, Jagiellonian University, Łojasiewicza 11, 30-348 Krakow, Poland
E-mail: leshi2022@zju.edu.cn
robert.podgajny@uj.edu.pl.

1. Experimental details 4

2. Composition and general remarks section 10

Figure S1. Infrared spectra of **1-4** 10
Figure S2. The representative SEM-EDS analysis for $\mathbf{1}^{\mathbf{de}}$ 12
Figure S3. The representative SEM-EDS analysis for $\mathbf{2}^{\mathbf{de}}$. 12
Figure S4. The representative SEM-EDS analysis for $\mathbf{3}^{\mathbf{de}}$ 13
Figure S5. The representative SEM-EDS analysis for $\mathbf{4}\boldsymbol{R}^{\mathbf{de}}$ 13
Figure S6. The representative SEM-EDS analysis for $\mathbf{4}\boldsymbol{S}^{\mathbf{de}}$. 14
Figure S7. Comparison of powder XRD and calculated SC-XRD patterns for **1**. 15
Figure S8. Comparison of powder XRD and calculated SC-XRD patterns for $\mathbf{1}^{\mathbf{de}}$. 15
Figure S9. Powder XRD revealed the reversible transformation process between **1** and $\mathbf{1}^{\mathbf{de}}$. 16
Figure S10. Comparison of powder XRD and calculated SC-XRD patterns for **2**. 16
Figure S11. Comparison of powder XRD and calculated SC-XRD patterns for **3.** 17
Figure S12. Comparison of powder XRD and calculated SC-XRD patterns of **4*S*** 18
Figure S13. Comparison of powder XRD and calculated SC-XRD patterns of **4*R*** 19
Figure S14. TGA curves of $\mathbf{1}^{\mathbf{de}}$ indicating the solvent weight loss 20
Figure S15. TGA curves of $\mathbf{2}^{\mathbf{de}}$ indicating the residue solvent weight loss 20
Figure S16. TGA curves of $\mathbf{3}^{\mathbf{de}}$ indicating the residue solvent weight loss 21
Figure S17. TGA curves of $\mathbf{4}\boldsymbol{R}^{\mathbf{de}}$ and $\mathbf{4}\boldsymbol{S}^{\mathbf{de}}$ indicating the residue solvent weight loss 21

3. Single crystal X-ray diffraction studies 22

Table S1. Crystal data and structure refinement of **1** and $\mathbf{1}^{\mathbf{de}}$ at different temperatures 22
Table S2. Crystal data and structure refinement of **2** and **3** at different temperatures. 23
Table S3. Crystal data and structure refinement of **4*S*** and **4*R*** at different temperatures... 24
Table S4. Results of Continuous Shape Measure Analysis for $[W(CN)_8]^{3-/4-}$ anions in **1-4**. 25
Figure S18 (a) Asymmetric unit of $\mathbf{1}^{\mathbf{LT}}$ 27
Figure S19 Asymmetric unit of $\mathbf{1}^{\mathbf{de\ LT}}$ 27
Figure S20 Asymmetric unit of $\mathbf{2}^{\mathbf{LT}}$. 28
Figure S21. Asymmetric unit of $\mathbf{3}^{\mathbf{LT}}$. 28
Figure S22. Asymmetric units of $\mathbf{4}\boldsymbol{S}^{\mathbf{LT}}$ (a) and $\mathbf{4}\boldsymbol{R}^{\mathbf{LT}}$ (b). 29

4. Supramolecular interactions and contacts. 30

Figure S23. C_{phenyl}-H…N_{CN} contacts between the neighboring clusters in **1** and $\mathbf{1}^{\mathbf{de}}$ 30
Figure S24. Crystal packing in **1** and $\mathbf{1}^{\mathbf{de}}$. 31
Figure S25. The projection of crystal packing of **1** and $\mathbf{1}^{\mathbf{de}}$ 32
Figure S26. The selected intercluster separation distances in **1** and $\mathbf{1}^{\mathbf{de}}$ 33
Table S5. Weak N_{CN}…H-C_{phenyl} supramolecular contacts in **1** and $\mathbf{1}^{\mathbf{de}}$ at 100 K. 34
Table S6. The most important hydrogen bonds and intermolecular contacts involving methanol molecules in the space close to the cluster vertex region and lateral region for **1** and $\mathbf{1}^{\mathbf{de}}$ at 100 K (Å, deg) 35
Figure S27. C_{phenyl}-H…N_{CN} contacts between the neighboring clusters in **2** and **3** 36
Figure S28. Crystal packing in **2** 37

Figure S29. Crystal packing in **3** 38
Table S7. Weak N_{CN}…H-C_{phenyl} supramolecular contacts in **2** and **3** at 100 K. 38
Figure S30. Supramolecular architecture of **4*S*** and **4*R*** within the *bc* plane 39
Figure S31. Crystal packing in **4*S*** and **4*R***.......... 40
Figure S32. The selected intercluster separation distances in **4*S*** and **4*R***.......... 41
Table S8. Hydrogen-bonding and weak intermolecular interactions or contacts in **4*S*** and **4*R*** at 100 K. 41
5. Bond length and angles parameters. 42
Table S9. Detailed structure bond lengths and bond angles of Fe^{II} ions for **1** and **1**^{de}.......... 42
Table S10. Detailed structure bond lengths and bond angles of Co^{II} ions for **2** and Ni ions for **3** at low and high temperatures. 44
Table S11. Detailed structure bond lengths and bond angles of Co^{II} ions for **4*R*** and **4*S***... 46
Table S12. Comparison of Fe-N_{CN}, Fe-N_{py} and Fe-N_{ami} bond lengths, average M-N bond lengths and octahedral distortion bond angle parameters ∑ in HT and LT phase of complexes **1**^{de} together with the Fe position in the cluster and spin state assignment. 48
Table S13. Comparison of Ni-N_{CN}, Ni-N_{py} and Ni-N_{ami} bond lengths, average M-N bond lengths and octahedral distortion bond angle parameters ∑ in HT and LT phase of complex **3**. 49
Table S14. Transition completeness of **1** and **1**^{de} 50
Figure S33. Overlays of the high spin (red) and low spin (blue) structures of **1** and **1**^{de} ... 51
6. Bond valence sum (BVS) calculations 52
Table S15. Bond valence sum (BVS) calculations for **1** and **1**^{de} 52
Table S16. Bond valence sum (BVS) calculations for **2** and **3**.......... 53
Table S17. Bond valence sum (BVS) calculations for **4*S*** and **4*R***.......... 54
7. Magnetic characteristics.......... 55
Figure S34. Expanded $\chi_M T(T)$ plot; *M* versus *H* plot.......... 55
Table S18. Ni-N≡C angles within the cyanido-bridged skeleton of **3**.......... 56
8. Spectroscopic studies. 57
Figure S35. Temperature dependent 57Fe Mössbauer spectra for **1** 57
Table S19. The most important parameters of ^{57}Fe Mössbauer spectra for **1**.......... 58
Figure S36. Temperature dependent ^{57}Fe Mössbauer spectra for **1**^{de}. 59
Table S20. The most important parameters of ^{57}Fe Mössbauer spectra for **1**^{de}. 60
Figure S37. Solid-state UV-Vis-NIR absorption spectra of **1** 61
Figure S38. Solid-state UV-Vis-NIR absorption spectra of **2** 62
Figure S39. Solid-state UV-Vis-NIR absorption spectra of **3** 63
Figure S40. Solid-state UV-Vis-NIR absorption spectra of **4*R*** and **4*S***.......... 64
Figure S41. UV-vis-NIR spectra of **3**. 65
Figure S42. UV-vis-NIR spectra of **4R** and **4S** 65
Figure S43. Electrospray mass spectrum of **1** 66
Figure S44. Electrospray mass spectrum of **2** 66
9. References.......... 67

1. Experimental details

Materials. 2-Pyridinecarboxaldehyde, (R)/(S)-(+)-1-(1-Naphthyl)ethylamine, $FeCl_2 \cdot 4H_2O$, $CoCl_2 \cdot 6H_2O$, $Co(ClO_4)_2 \cdot 6H_2O$, $Ni(ClO_4) \cdot 6H_2O$ and methanol used during the synthesis were purchased from commercial sources (Sigma-Aldrich Idalia, CO) and used without further purification. $TBA_3[W(CN)_8]$ and $(Bu_3N)_3[W(CN)_8]$ were prepared following literature procedure.[1-2] The organic reagents, 2-(Chloromethyl)pyridine hydrochloride, benzaldehyde, ethylenediamine were purchased from Sigma-Aldrich and used without purification. N^1, N^2-dibenzyl-N^1, N^2-bis(pyridin-2-ylmethyl)ethane-1,2-diamine (bzbpen) and (R)/(S)-N-(1-(naphthalen-1-yl)ethyl)-1-(pyridin-2-yl)methanimine was synthesized by modification of the published procedure.[3-4]

***Caution!** Cyanides are highly toxic; they should be handled in small quantities with care.*

Syntheses of $Fe^{II}[Fe(bzbpen)]_6[W^{V}(CN)_8]_2[W^{IV}(CN)_8]_2 \cdot 18MeOH$ (1). 20.0 mg (0.05 mmol) bzbpen and 9.9 mg (0.05 mmol) $FeCl_2 \cdot 4H_2O$ were dissolved in 2.0 ml methanol, and stirred for 2 minutes to give the yellowish solution. Then, the freshly prepared methanolic (2.0 ml) solution of 37.5 mg (0.033 mmol) of $TBA_3[W(CN)_8]$ was slowly added. The resulting dark brown solution was tightly closed and left in dark surrounding. The square shaped brownish green crystals appeared after one week. The crystals were identified as $Fe^{II}[Fe^{II}(bzbpen)]_6[W^{V}(CN)_8]_2[W^{IV}(CN)_8]_2 \cdot 18MeOH$ (M_w = 5071.09 g mol^{-1}) by SC-XRD experiment performed on crystals protected by Apiezon N grease. Phase purity was proved by powder XRD data (*see* Figure S7). IR (cm^{-1}). CN^- stretching vibrations: 2172s, 2145s, 2111s, 2079s. Compound **1** is stable in the mother solution, but tends to lose some of crystallized methanol molecules in air. The formula of the partially desolvated form $Fe^{II}[Fe^{II}(bzbpen)]_6[W^{V}(CN)_8]_2[W^{IV}(CN)_8]_2 \cdot 5MeOH$ (**1de**) was obtained by drying **1** for 20 minutes in the steam of dry N_2 at 300 K. In case of single crystal X-ray diffraction measurement, crystal of **1** was dried for 20 minutes in diffractometer under a dry nitrogen flow at 300 K, and on *in situ* generated single crystal of **1de** at various

temperatures (300 K → 100 K (1de). Phase purity was proved by powder XRD data (*see* Figure S8). IR (cm^{-1}). CN^- stretching vibrations: 2172(s), 2143(s), 2106(s), 2080(s). Elemental analysis for $\mathbf{1^{de}}$ found (calcd for $C_{205}H_{200}Fe_7N_{56}O_5W_4$) (%) C, 52.37 (52.90), N, 16.58 (16.85), H, 4.26 (4.33). The difference between calculated and found amounts of C larger than 0.3% probably due to the absorption of two additional H_2O molecules from air during the measurement, which give $C_{205}H_{204}Fe_7N_{56}O_7W_4$: Cal. (%) C, 52.49; N, 16.72; H, 4.38. Thermal gravity analysis shown the 3.09% weight loss (The expected weight loss is 3.43%), corresponding to remove 5 methanol molecules (*see* Figure S14). **1** and $\mathbf{1^{de}}$ are soluble in MeCN, $MeNO_2$, DMF and DMSO to give a yellowish green solution.

Syntheses of $Na^I[Co^{II}(bzbpen)]_6[W^V(CN)_8]_3[W^{IV}(CN)_8]\cdot 28MeOH$ (2). 20.0 mg (0.05 mmol) bzbpen and 18.0 mg (0.05 mmol) $Co(ClO_4)_2\cdot 6H_2O$ were dissolved in 3.0 ml methanol, and stirred for 2 minutes to give the pink solution. The freshly prepared methanolic (2.0 ml) solution of 30 mg (0.033 mmol) of $(Bu_3N)_3[W(CN)_8]$ was slowly added. The resulting dark brown suspension was stirred for 5 minutes. Then, 1.0 mL of methanol solution containing 6.5 mg of NaN_3 was slowly added to form a clear brownish orange solution. The solution was tightly closed and left to crystallization. The square shaped brownish orange crystals obtained after two days. The crystals were identified as $Na^I[Co^{II}(bzbpen)]_6[W^V(CN)_8]_3[W^{IV}(CN)_8]\cdot 28MeOH$ (M_w = 5377.13 g mol^{-1}) by SC-XRD experiment performed on crystals protected by Apiezon N grease. Reasonable phase purity was confirmed by powder XRD data (Figure S10). IR (cm^{-1}), CN^- stretching vibrations: 2187(s), 2162(s), 2132(s), 2121(s). The crystals of **2** are stable in the mother solution but exposure to the air leads to breakage of the crystal. The air-stable composition of $Na[Co(bzbpen)]_6[W(CN)_8]_4\cdot 17H_2O$ ($\mathbf{2^{de}}$) was derived from the CHN elemental analysis. Thermal gravity analysis shown the 5.83% weight loss (expected weight loss is 6.39%, corresponding to remove 17 water molecules, Figure S15, SI). Anal. Calcd. for $W_4Co_6NaC_{200}H_{214}N_{56}O_{17}$ (M_w = 4786.1554 g mol^{-1}), (%) C, 50.18; N, 16.38; H, 4.51. Found: C, 50.41; N, 16.09; H, 4.324. Metallic composition ratio, Na : Co : W = 1.24 : 6.04 : 4 found from SEM/EDS; expected 1 : 6 : 4. Na : Co = 1.34 : 6 found from FAAS; expected 1 : 6. **2** is soluble in MeCN, $MeNO_2$, DMF and DMSO, expected to give a light violet solution.

Syntheses of $Na^{I}[Ni^{II}(bzbpen)]_6[W^{V}(CN)_8]_3[W^{IV}(CN)_8]\cdot 27MeOH$ (3). 20.0 mg (0.05 mmol) bzbpen and 18.0 mg (0.05 mmol) $Ni(ClO_4)_2\cdot 6H_2O$ were dissolved in 3.0 ml methanol, and stirred for 2 minutes to give the yellowish green solution. The freshly prepared methanolic (3.0 ml) solution of 37.5 mg (0.033 mmol) of $TBA_3[W(CN)_8]$ $[W(CN)_8]$ was slowly added and produced a large amount of yellow precipitate. Then, 1.0 ml of methanol containing 13.0 mg of NaN_3 was slowly added to the above solution and the yellow precipitate gradually dissolved to form a clear brownish yellow solution. The solution was tightly closed and left crystallization in the vial. The well-shaped but very fragile yellow crystals appeared at the bottom of the vial after three days. The crystals were identified as $Na^{I}[Ni^{II}(bzbpen)]_6[W^{V}(CN)_8]_3[W^{IV}(CN)_8]\cdot 27MeOH$ (M_w = 5344.26 g mol^{-1}) by SC-XRD experiment performed on crystals protected by Apiezon N grease. Reasonable phase purity was confirmed by powder XRD data (Figure S11). IR (cm^{-1}). CN^- stretching vibrations: 2187(s), 2162(s), 2132(s), 2121(s). The crystals of **3** are stable in the mother solution but exposure to the air leads to breakage of the crystal. The air-stable composition of $\{Na[Ni(bzbpen)]_6[W(CN)_8]_4\}\cdot 8H_2O$ (**3**$^{\mathbf{de}}$) was derived from the CHN elemental analysis. Thermal gravity analysis shown the 3.23% weight loss (The expected weight loss is 3.78%, corresponding to remove 1 methanol and 8 water molecules, Figure S16, SI). Anal. Calcd. for $W_4Ni_6NaC_{201}H_{200}N_{56}O_9$ (M_w = 4654.62 g mol^{-1}), (%) C, 51.86; N, 16.85; H, 4.33. Found: C, 51.73; N, 16.90; H, 4.494. Metallic composition ratio, Na : Ni : W, 1.18 : 5.92 : 4 found from SEM/EDS; expected 1 : 6 : 4. Na : Ni, 1.37 : 6 found form FAAS; expected 1 : 6. **3** is soluble in MeCN, $MeNO_2$, DMF and DMSO to give a pale-yellow solution.

$[Co^{II}(S/R\text{-}pabh)_2]_6[W^{V}(CN)_8]_2[W^{IV}(CN)_8]_2\cdot 26MeOH$ (4*S* and 4*R*). The 25.6 mg (0.15 mmol) portion of (*R*)/(*S*)-(+)-1-(1-Naphthyl)ethylamine was dissolved together with excess 2-pydinealdehyde (25 mg, 22.7 mmol) in MeOH (2.0 mL). The resulting pale-yellow solution was treated with ultrasonic for *ca*. 3 minutes. Then, the methanolic (2.0 mL) solution of $CoCl_2\cdot 6H_2O$ (11.8 mg, 0.05 mmol) was slowly added to obtain an orange solution. The freshly prepared methanolic (2.0 mL) solution of $TBA_3[W(CN)_8]$ (37.5 mg, 0.033 mmol) was added. The resulting dark violet solution was gently stirred for a few minutes and left closed for crystallization in the dark. The well-shaped dark-violet crystals appeared after a few days. The crystals were identified as $Co^{II}[Co^{II}($*S/R*-

pabh)$_2$]$_6$[$W^V(CN)_8$]$_2$[$W^{IV}(CN)_8$]$_2$·26MeOH (M_w = 5937.56 g mol^{-1}, **4*S*** and **4*R***) by crystal structure solution and refinement using the SC-XRD data. Phase purity was proved by powder XRD data (Figure S12-S13). IR (cm^{-1}), CN^- stretching vibrations: 2189(s), 2172(s), 2150(s), 2118(s) for **4*S*** and 2191(s), 2173(s), 2151(s), 2120(s) for **4*R***, respectively.

The crystals are stable in the mother solution but exposure to the air leads to breakage of the crystal. The air-stable composition of {Co^{II}[Co^{II}(*S/R*-pabh)$_2$]$_6$[$W(CN)_8$]$_4$}·2MeOH·17H_2O (**4*S*^{de}** and **4*R*^{de}**) was derived from CHN elemental analysis. Thermal gravity analysis shown the 6.12% weight loss (The expected weight loss is 6.75%, corresponding to remove 2 methanol molecules and 17 water molecules, Figure S17, SI). Elemental analysis found: C: 54.92%; N: 14.35%; H: 4.27%. for **4*S*^{de}** and C: 54.49%; N: 14.34%; H: 4.09% for **4*R*^{de}**.respectively. Calcd for $C_{250}H_{234}Co_7N_{56}O_{19}W_4$ (M_w = 5474.79 g mol^{-1}): C, 54.85%; N, 14.33%; H, 4.31%. **4*R*** and **4*S*** are soluble in DMF and DMSO and slightly soluble in MeCN and $MeNO_2$, to give a dark violet solution.

Physical Techniques. CHN elemental analyses for the desolvated samples of **1de-4de** were performed on an Elemental Vario Micro Cube CHNS analyzer. TGA studies for **1de-4de** were carried out by using a Mettler Toledo TGA1 instrument. The SEM-EDS measurements were performed using a JEOL-5410 SEM microscope equipped with an EDS NORAN Voyager 3100 add-on. The contents of cobalt, sodium, nickel were determined by flame atomic absorption spectrometer (PinAAcle, Perkin Elmer) in air-acetylene flame, at a wavelength of 240.73 nm, 589.00 nm and 232.00 nm, respectively. Samples of an approximate weight of 7 mg were digested with 5 mL of nitric acid (suprapure, Merck), using high-pressure microwave digestion sytem (Magnum II, ERTEC, Poland). After digestion, the samples were diluted to 25 ml with deionized water.

To prevent the decomposition of the investigated material in the solid state by spectroscopic techniques the most of further physical measurements were further performed on the crystals placed in the mother liquid (**1-4@MeOH**), or dispersed in paraffin oil and Apiezon N grease. Powder X-ray diffraction patterns for **1-4@MeOH** sealed in glass capillary (0.5 mm) were collected on a PANalytical X'Pert PRO MPD

diffractometer with **Debye**-Scherrer geometry using CuKα radiation (λ = 1.54187 Å; 2*θ* range: 3-50° for RT). The IR spectra were collected using a Nicolet iN10 MX FT-IR microscope in transmission. Measurements were performed on small single crystals covered by paraffin oil in order to avoid desolvation. The UV-vis-NIR spectra were collected on a JASCO V-670 spectrometer for samples **1-4** suspended in paraffin oil, and spread between two glass rollers. **1de** was measured using $BaSO_4$ pellets as the reference background. Natural circular dichroism (NCD) spectra for **4*R*** and **4*S*** (dispersed in Nujol with mother solution, 5-10 mg per several drops, and introduced between CaF_2 plates) were collected using a Jasco J-810 spectropolarimeter. The recorded signals were corrected using the blank Nujol signal. The transmission ^{57}Fe Mössbauer spectra were collected in 1024 channels, with a 10 mCi ^{57}Co source in an Rh matrix, using a Wissel spectrometer with a bath liquid nitrogen cryostat for selected temperatures in the range between 250 and 80 K. The temperature stabilization was better than 0.5 K. The *RT* measurements were performed also with the use of the Wissel spectrometer, but without cryostat, with the temperature stabilization +/–2K. The velocity scale was calibrated using the α-Fe foil standard. The ground sample of **1** with a tiny amount of the mother liquor was mixed with Apiezon N grease, whereas **1de** was prepared as a powder pellet. Both samples were sealed by Kapton foils in copper rings. Due to the small resonant effect in the studied complexes, a background contribution to the spectra from iron impurities of the beryllium windows of the cryostat system was precisely measured in a dummy run and was included as a fixed parameter in the fitting procedure. The background spectra of the sample holders did not reveal any significant contribution to the main spectra. Mössbauer spectra were fitted with the use of the WinNormos-for-Igor software package, assuming the Lorentzian shape of the resonance lines, *i.e.* the saturation effects were not included. Two types of quadrupole doublets were considered in the fits, assigned to the $^{LS}Fe^{II}$ and $^{HS}Fe^{II}$, respectively. The contribution of the HS states may consist of more components, indistinguishable within measured spectra due to their low intensities. The relative fractions of the HS versus LS states were determined from the ratio of the areas of the corresponding doublets, which means that the equal recoil-free fractions of iron in both spin states were assumed. Magnetic susceptibility measurements were probed with Quantum Design MPMS 3

SQUID magnetometer. Freshly prepared **1-4** microcrystalline samples were sealed with a small amount of mother liquor in a glass tube to prevent lost solvent, whereas sample of **1**^{de} placed in a foil bag, which was immediately sealed after desolvation. The dc magnetic susceptibilities were measured using the thermal cyclic course 300 K → LT (50 K for Fe analogue **1**, and 200 K for Co analogues **2** and **4*S***) → 320 K → LT → 330 K → 300 K for **1**, **2** and **4*S***, in the range 2.0 – 320 K for **3** and **4*R***, and in the range 2.0-300 K for **1**^{de}, with an applied field of 1000 Oe. Isothermal magnetizations were collected at 2.0 K in 0 – 70 kOe applied dc field range. The magnetic data were corrected for diamagnetic contribution of the container and those of the sample, by empirical values and by Pascal's constants, [5] respectively.

Crystal Structure Determination. Single crystal data of **1**–**4** was collected using a Bruker D8 Quest Eco diffractometer equipped with a curved TRIUMPH focusing monochromator of Mo Kα (λ = 0.71073 Å) radiation, and a highly sensitive CPAD Photon II detector, coupled with a CryoStream 800 Plus (Oxford Cryosystems) temperature controller. Crystals of **1-4** for measurement in 100 K and 250 K were taken from mother solution and covered by NVH immersion oil in order to prevent the loss of crystallization methanol molecules. Data reduction and cell parameter refinement were performed using Apex software with included SAINT and SADABS programs. Intensities of reflections for the sample absorption were corrected using multi-scan method. Structures were solved by intrinsic phasing method and refined anisotropically with weighted full-matrix least squares on F^2 using SHELXT [6] and SHELXL [7] programs with Olex 2 graphic interface.[8]

For **1** and **1**^{de}, all non-hydrogen atoms were refined anisotropically with restraints (DFIX, ISOR, SIMU and DELU) on methanol molecules and partially phenyl groups. Hydrogen atoms within structures were placed in idealized positions and refined using riding coordinate model. While methanol molecules in **4*R*** and **4*S*** were refined isotropically. A solvent mask procedure was further performed on **2** and **3** dues to highly disordered interstitial solvent molecules. Crystal data and structure refinement parameters are summarized in Table S1-S3. The structural figures in article were prepared using Mercury [9], Olex 2 [8] and Diamond 3.2 software. Continuous shape measure analysis of coordination spheres was performed using SHAPE 2.1. [10] The

crystal structures are deposited in CCDC data base. The deposition number are 2190783-2190794, respectively.

2. Composition and general remarks section

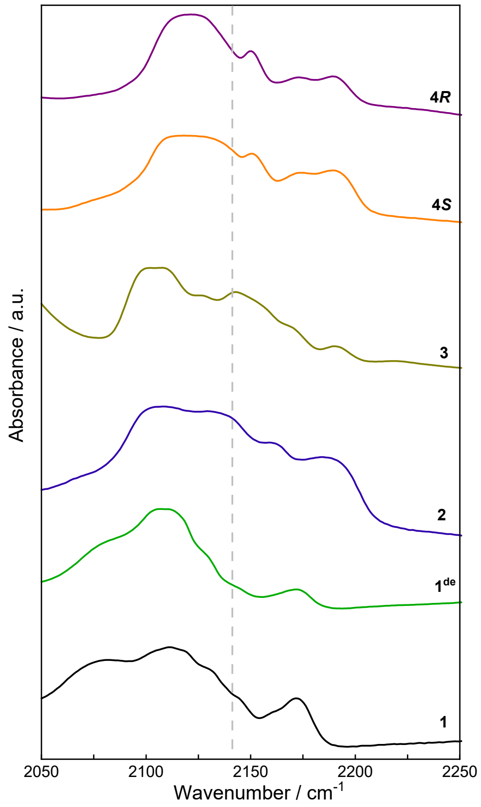

Figure S1. Infrared spectra of **1-4** at room temperature in the *v*(CN) vibration region.

All compounds exhibit significant absorption in the range of 2050-2250 cm^{-1} characteristic to the stretching v(CN) vibrations. All patterns cover the broad range of the wavelength scale, from *ca.* 2070 to *ca.* 2180 cm^{-1} in the case of **1** and **1**$^{\mathbf{des}}$, from *ca.* 2090 to *ca.* 2200 cm^{-1} in the case of **2** and **3**, and from *ca.* 2100 to *ca.* 2210 cm^{-1} in the case of **4*R*** and **4*S***. The peaks located in the range of 2140-2200 cm^{-1} are assigned to the $[W(CN)_8]^{3-}$ anions and W^{V}-CN-M^{II} linkages, whereas those located in the range of 2050-2140 cm^{-1} are assignable to $[W(CN)_8]^{4-}$ anions and W^{IV}-CN-M^{II} linkages. Thus, the observed patterns adequately illustrate the complex character of our systems, considering in particular the mixed valence character of the electronic ground state and possible MMCT charge transfer through the cyanido-bridges suggested by the NIR

absorption in the electronic absorption spectra (compare Figure 5d).

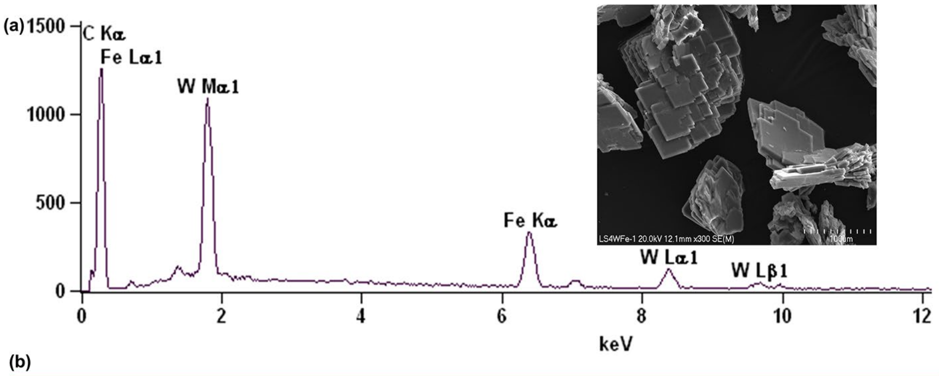

(b)

Element Line	Net Counts	Net Counts Error	Weight %	Weight % Error	Atom %	Atom % Error	Normalized Composition	Expected Composition
Fe K	4965	+/- 160	35.95	+/- 1.16	64.88	+/- 2.09	**7.39**	7
W L	2871	+/- 196	64.05	+/- 4.37	35.12	+/- 2.40	**4**	4
Total			100.00		100.00			

Figure S2. The representative SEM-EDS analysis for **1**$^{\mathbf{de}}$. (a) EDS spectra collected from the **1**$^{\mathbf{de}}$. Inset: SEM image (300-fold magnification); (b) Results of SEM-EDS analysis of metal ions composition. The found metallic composition Fe : W of 7.4 : 4 conforms within the experimental error the expected composition 7 : 4 deduced from the structural data.

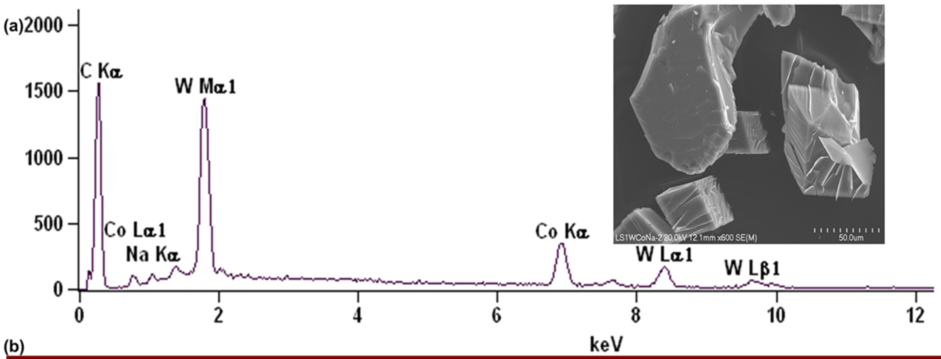

(b)

Element Line	Net Counts	Net Counts Error	Weight %	Weight % Error	Atom %	Atom % Error	Normalized Composition	Expected Composition
Na K	361	+/- 59	2.52	+/- 0.41	10.85	+/- 1.77	**1.23**	1
Co K	5765	+/- 182	32.10	+/- 1.01	53.94	+/- 1.70	**6.13**	6
W L	4316	+/- 235	65.38	+/- 3.56	35.21	+/- 1.92	**4**	4
Total			100.00		100.00			

Figure S3. The representative SEM-EDS analysis for **2**$^{\mathbf{de}}$. (a) EDS spectra collected from the **2**$^{\mathbf{de}}$. Inset: SEM image (600-fold magnification); (b) Results of SEM-EDS analysis of metal ions composition. The found metallic composition Na : Co : W of 1.24 : 6.04 : 4 conforms within the experimental error the expected composition 1 : 6 : 4. This composition was confirmed by the FAAS examination of the Na : Ni atomic ratio: found 0.288 : 1.29 (1.34 : 6) (based on the contents, in mg/g, 6.62 ± 0.29 of Na, and 76.42 ± 0.64 of Co), expected 1 : 6.

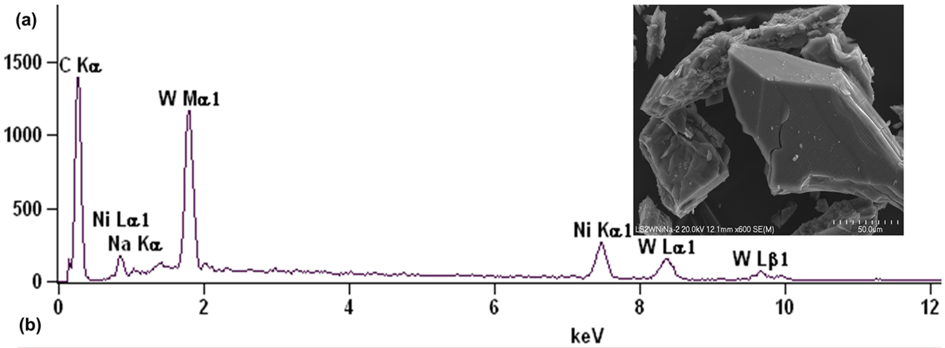

(b)

Element Line	Net Counts	Net Counts Error	Weight %	Weight % Error	Atom %	Atom % Error	Normalized Composition	Expected Composition
Na K	269	+/- 42	2.45	+/- 0.38	10.67	+/- 1.67	**1.18**	1
Ni K	4158	+/- 162	31.29	+/- 1.22	53.30	+/- 2.08	**5.92**	6
W L	3516	+/- 229	66.25	+/- 4.32	36.03	+/- 2.35	**4**	4
Total			100.00		100.00			

Figure S4. The representative SEM-EDS analysis for **3**$^{\mathbf{de}}$. (a) EDS spectra collected from the **3**$^{\mathbf{de}}$. Inset: SEM image (600-fold magnification); (b) Results of SEM-EDS analysis of metal ions composition. The found metallic composition Na : Ni : W of 1.18 : 5.92 : 4 conforms within the experimental error the expected composition 1 : 6 : 4. This composition was confirmed by the FAAS examination of the Na : Ni atomic ratio: found 0.289 : 1.26 (1.37 : 6) (based on the contents, in mg/g, 6.59 ± 0.28 of Na, and 74.30 ± 0.19 of Ni), expected 1 : 6.

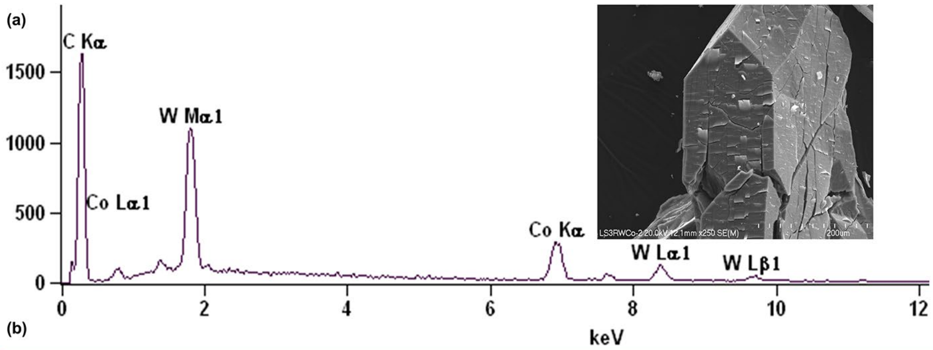

(b)

Element Line	Net Counts	Net Counts Error	Weight %	Weight % Error	Atom %	Atom % Error	Normalized Composition	Expected Composition
Co K	4741	+/- 161	37.24	+/- 1.26	64.93	+/- 2.20	**7.41**	7
W L	2924	+/- 198	62.76	+/- 4.25	35.07	+/- 2.37	**4**	4
Total			100.00		100.00			

Figure S5. The representative SEM-EDS analysis for **4*R***$^{\mathbf{de}}$. (a) EDS spectra collected from the **4*R***$^{\mathbf{de}}$. Inset: SEM image (250-fold magnification); (b) Results of SEM-EDS analysis of metal ions composition. The found metallic composition Co : W of 7.4 : 4 conform within the experimental error the expected composition 7 : 4.

(a)

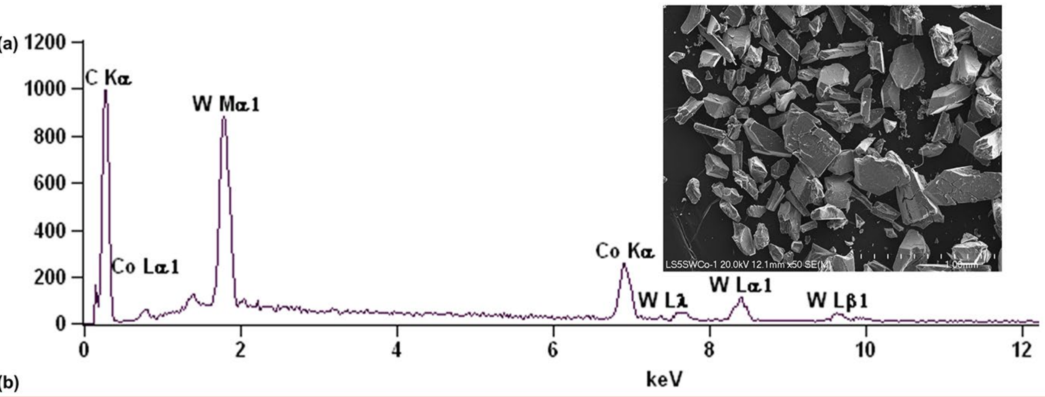

(b)

Element Line	*Net Counts*	*Net Counts Error*	*Weight %*	*Weight % Error*	*Atom %*	*Atom % Error*	*Normalized Composition*	*Expected Composition*
Co K	3969	+/- 148	36.57	+/- 1.36	64.27	+/- 2.40	**7.19**	7
W L	2522	+/- 182	63.43	+/- 4.58	35.73	+/- 2.58	**4**	4
Total			100.00		100.00			

Figure S6. The representative SEM-EDS analysis for **4*S*^{de}**. (a) EDS spectra collected from the **4*S*^{de}**. Inset: SEM image (50-fold magnification); (b) Results of SEM-EDS analysis of metal ions composition. The found metallic composition Co : W of 7.2 : 4 conform within the experimental error the expected composition 7 : 4.

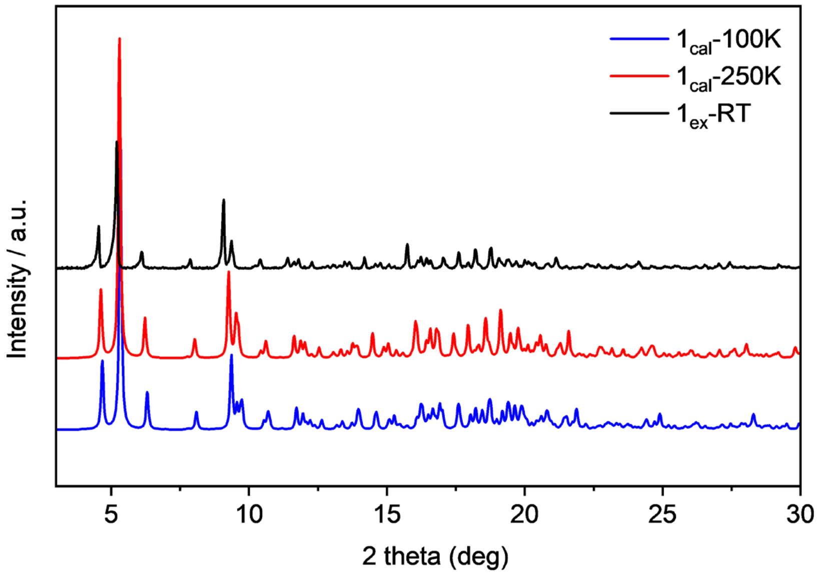

Figure S7. Comparison of powder XRD and calculated SC-XRD patterns for **1**.

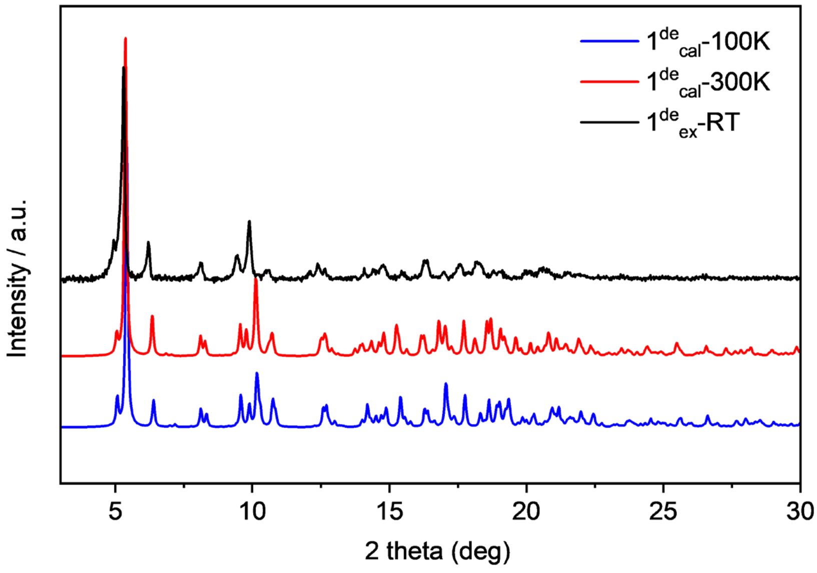

Figure S8. Comparison of powder XRD and calculated SC-XRD patterns for $\mathbf{1}^{de}$.

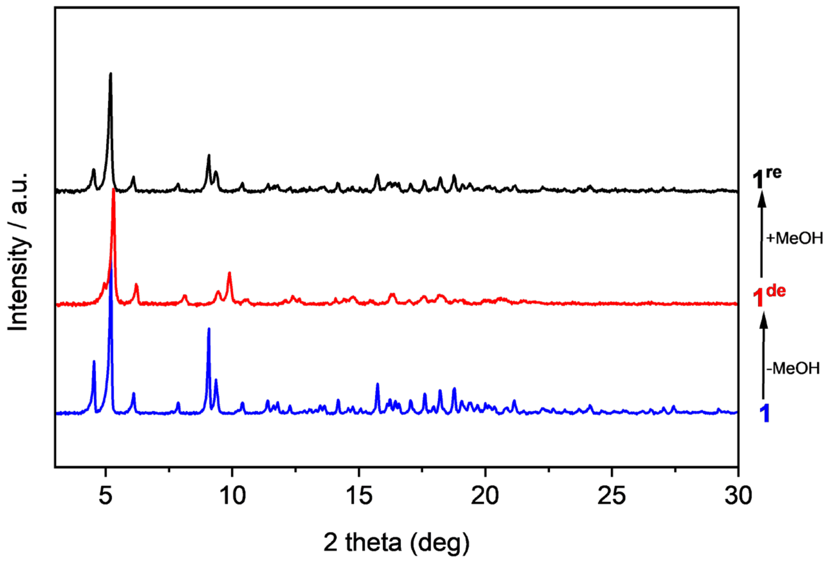

Figure S9. Powder XRD revealed the reversible transformation process between **1** and **1**^{de}.

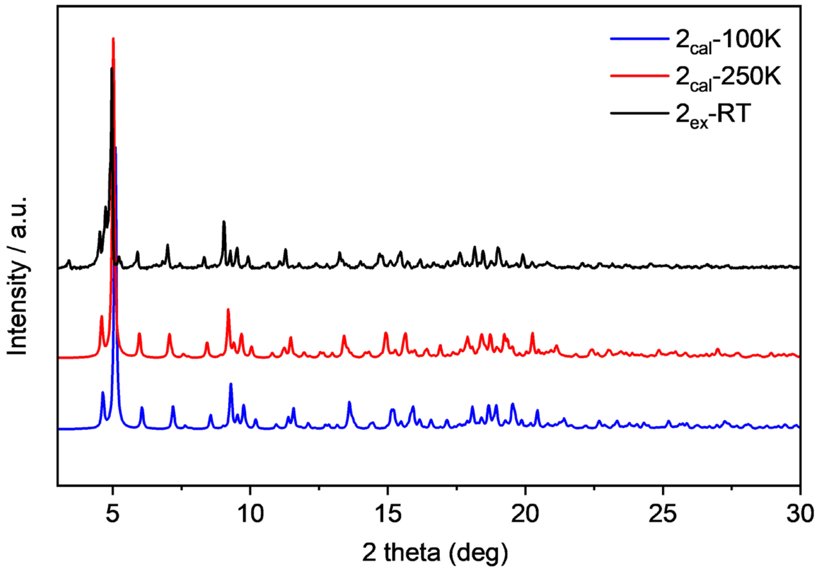

Figure S10. Comparison of powder XRD and calculated SC-XRD patterns for **2**. The additional peaks in experimental results are most probably indicative for the SCO transition, the onset of which is observed in magnetic data (compare magnetic data in Figure 4).

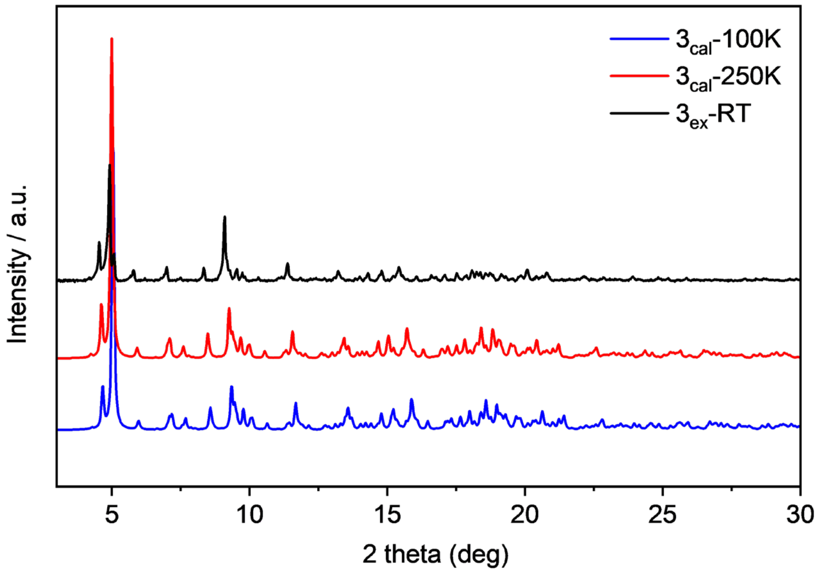

Figure S11. Comparison of powder XRD and calculated SC-XRD patterns for **3.** The additional peaks (*ca.* 5.1 deg) in experimental results might be due to small amounts of impurities.

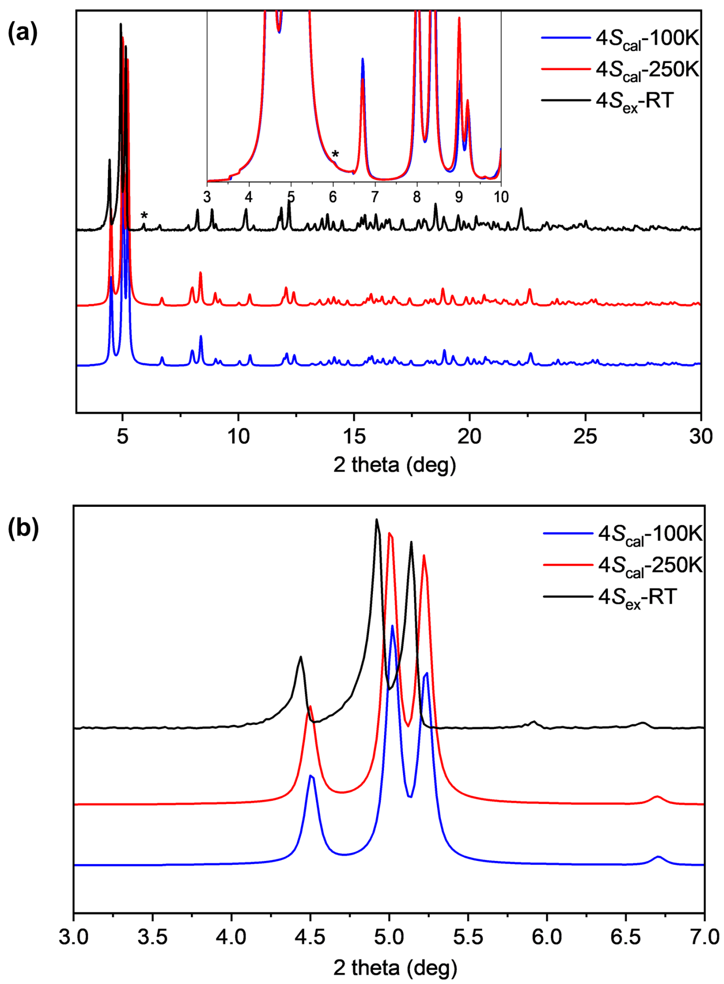

Figure S12. Comparison of powder XRD and calculated SC-XRD patterns of **4*S*** presented in the broad 2θ range of 3° - 30° (a) and in the limited low angle 2θ range of 3° - 7° (b). Inset: expanded plot exhibits a small peak at 6.0 (deg), in agreement with the experimental results.

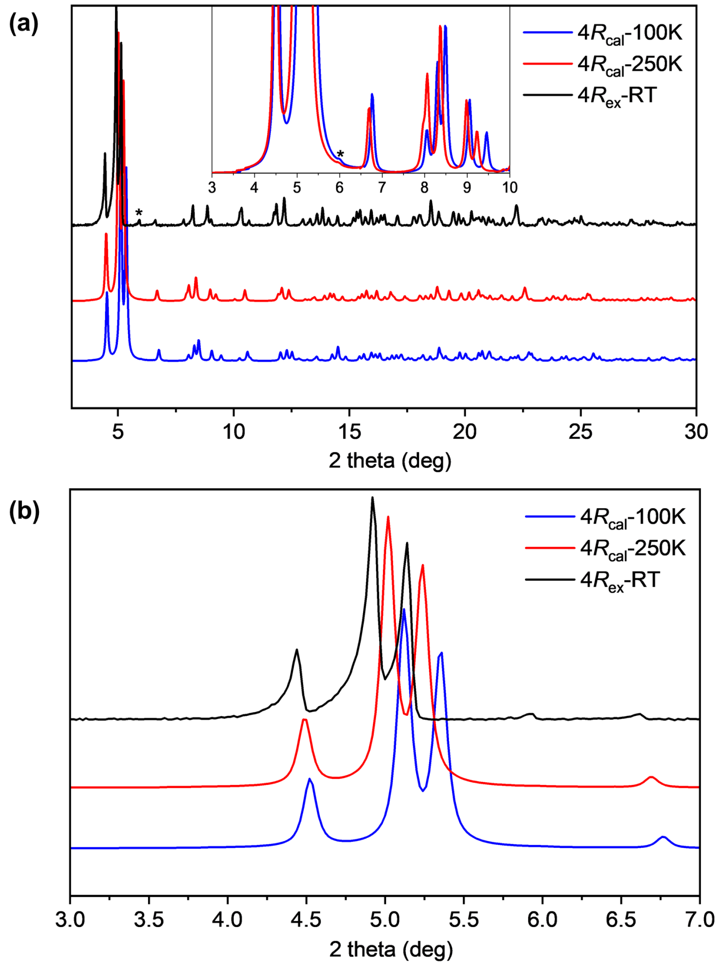

Figure S13. Comparison of powder XRD and calculated SC-XRD patterns of ***4R*** presented in the broad 2θ range of 3° - 30° (a) and in the limited low angle 2θ range of 3° - 7° (b). Inset: expanded plot exhibits a small peak at 6.0 (deg), in agreement with the experimental results.

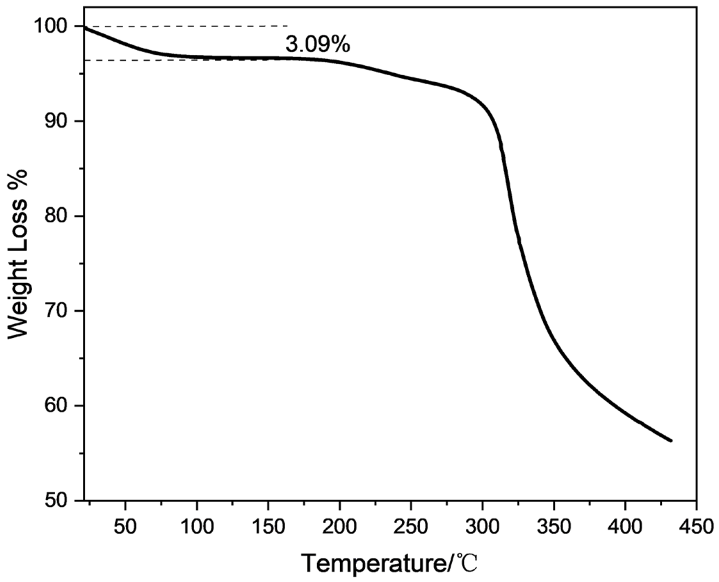

Figure S14. TGA curves of **1**^{de} indicating the solvent weight loss. The observed weight loss of 3.09% is slightly smaller than 3.43% expected for 5 MeOH molecules in dry crystals of **1**^{de}. This difference is probably due to some weight loss preceding the measurements in RT.

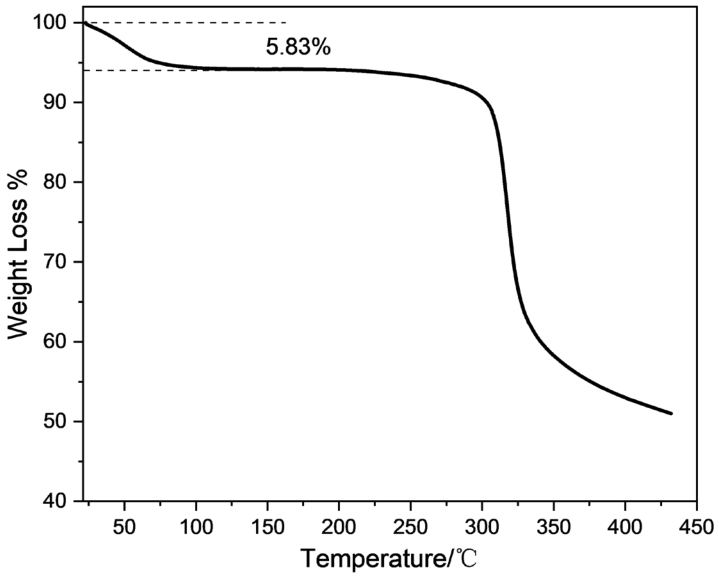

Figure S15. TGA curves of **2**^{de} indicating the residue solvent weight loss. The observed weight loss of 5.83% is slightly smaller than 6.39% expected for 17 H_2O molecules in dry residue of **2**^{de}. This difference is probably due to some weight loss preceding the measurements in RT.

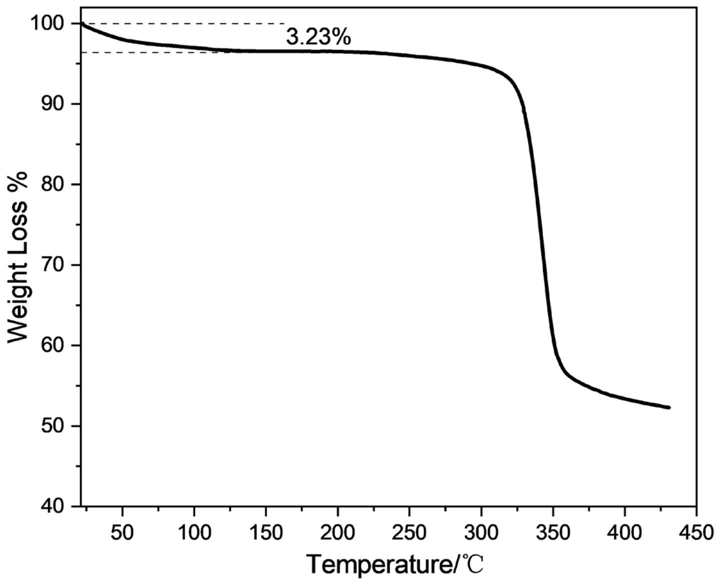

Figure S16. TGA curves of **3**^{de} indicating the residue solvent weight loss. The observed weight loss of 3.23% is slightly smaller than 3.78% expected for 1 MeOH molecule and 8 H_2O in dry residue of **3**^{de}. This difference is probably due to some weight loss preceding the measurements in RT.

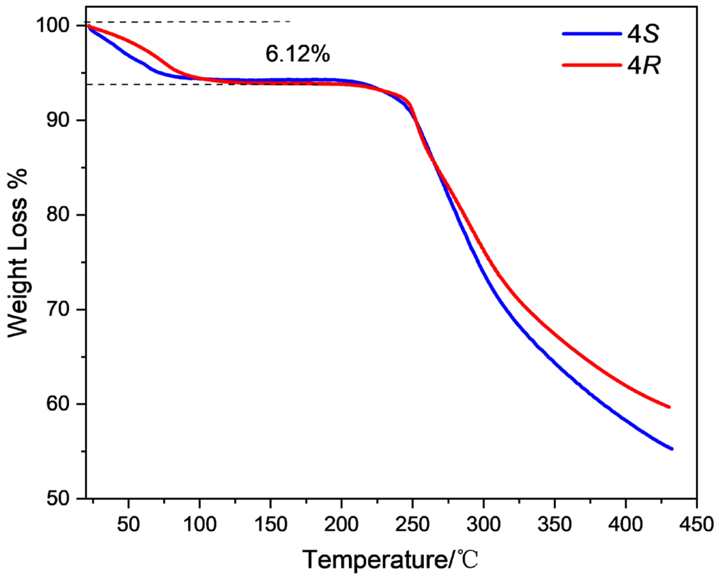

Figure S17. TGA curves of **4*R***^{de} and **4*S***^{de} indicating the residue solvent weight loss. The observed weight loss of 6.12% is slightly smaller than 6.75% expected for 2 MeOH molecule and 17 H_2O in dry residues. This difference is probably due to some weight loss preceding the measurements in RT.

3. Single crystal X-ray diffraction studies

Table S1. Crystal data and structure refinement of **1** and **1de** at different temperatures. The significant *ca.* 10% shortening of the period a is indicated in bold style.

Compound	1		1^{de}	
CCDC	2190783	2190784	2190785	2190786
T/K	100	250	100	300
Empirical formula	$C_{218}H_{252}Fe_7N_{56}O_{18}W_4$	$C_{218}H_{252}Fe_7N_{56}O_{18}W_4$	$C_{205}H_{200}Fe_7N_{56}O_5W_4$	$C_{205}H_{200}Fe_7N_{56}O_5W_4$
Formula weight	5071.09	5071.09	4654.55	4654.55
Crystal system	monoclinic	monoclinic	monoclinic	monoclinic
Space group	*C*2/*c*	*C*2/*c*	*C*2/*c*	*C*2/*c*
**a/Å**	**40.2528(10)**	**40.5157(11)**	**36.8073(8)**	**37.042(3)**
b/Å	21.8425(6)	22.0105(7)	21.7499(5)	21.7842(18)
c/Å	26.8457(6)	27.1018(7)	26.7178(7)	27.2718(19)
α/°	90	90	90	90
β/°	110.2370(10)	109.5250(10)	108.9810(10)	109.302(2)
γ/°	90	90	90	90
Volume/Å^3	22146.3(10)	22778.8(11)	20226.1(8)	20769(3)
Z	4	4	4	4
ρ_{calc}, g/cm^3	1.521	1.479	1.529	1.489
μ/mm^{-1}	2.586	2.514	2.820	2.746
F(000)	10296.0	10296.0	9360.0	9360.0
Radiation	MoKα (λ = 0.71073)	MoKα (λ = 0.71073)	MoKα (λ = 0.71073)	MoKα (λ = 0.71073)
2θ range/°	4.184 to 50	4.886 to 50	4.432 to 50	4.414 to 50
Index ranges	-47≤h≤47<br>-25≤k≤25<br>-30≤l≤31	-48≤h≤47<br>-18≤k≤26<br>-32≤l≤31	-26≤h≤26<br>-30≤k≤30<br>-34≤l≤29	-43≤h≤43<br>-21≤k≤25<br>-30≤l≤32
Reflections collected	89059	67306	40516	63221
Independent reflections	19463 [R_{int} = 0.0510, R_{sigma} = 0.0448]	20046 [R_{int} = 0.0460, R_{sigma} = 0.0548]	17752 [R_{int} = 0.0370, R_{sigma} = 0.0587]	18242 [R_{int} = 0.0666, R_{sigma} = 0.0778]
Data/restraints/ parameters	19463/212/1390	20046/206/1390	17752/186/1246	18242/186/1246
GOF on F^2	1.062	1.035	1.022	1.025
R indexes [I>=2σ(I)]	R_1 = 0.0528, wR_2 = 0.1116	R_1 = 0.0495, wR_2 = 0.1026	R_1 = 0.0496, wR_2 = 0.1083	R_1 = 0.0548, wR_2 = 0.1119
R indexes [all data]	R_1 = 0.0680, wR_2 = 0.1179	R_1 = 0.0733, wR_2 = 0.1116	R_1 = 0.0736, wR_2 = 0.1193	R_1 = 0.0891, wR_2 = 0.1251
Largest diff. peak/hole, eA^{-3}	3.16/-1.83	1.91/-1.12	3.65/-1.30	1.54/-1.10

Table S2. Crystal data and structure refinement of **2** and **3** at different temperatures.

Compound	**2**		**3**	
CCDC	2190787	2190788	2190789	2190790
T/K	100	250	100	250
Empirical formula	$C_{228}H_{292}Co_6N_{56}O_{28}W_4Na$	$C_{228}H_{292}Co_6N_{56}O_{28}W_4Na$	$C_{227}H_{288}Ni_6N_{56}O_{27}W_4Na$	$C_{227}H_{288}Ni_6N_{56}O_{27}W_4Na$
Formula weight	5377.13	5377.13	5343.77	5343.77
Crystal system	monoclinic	monoclinic	monoclinic	monoclinic
Space group	*C*2/*c*	*C*2/*c*	$P2_1/c$	$P2_1/c$
a/Å	40.1967(9)	40.506(17)	39.7952(9)	40.194(2)
b/Å	24.5767(7)	25.015(11)	24.5667(6)	24.8404(14)
c/Å	25.8374(8)	26.150(13)	26.5942(6)	26.8077(14)
α/°	90	90	90	90
β/°	108.8890(10)	108.540(15)	108.1590(10)	108.271(2)
γ/°	90	90	90	90
Volume/Å^3	24150.2(12)	25122(20)	24704.6(10)	25416(2)
Z	4	4	4	4
ρ_{calc}, g/cm^3	1.479	1.422	1.437	1.397
μ/mm^{-1}	2.375	2.283	2.375	2.309
F(000)	10980.0	10980.0	10932.0	10932.0
Radiation	MoKα (λ = 0.71073)	MoKα (λ = 0.71073)	MoKα (λ = 0.71076)	MoKα (λ = 0.71076)
2θ range/°	4.696 to 50.126	4.626 to 50.104	4.426 to 50.08	4.38 to 50.106
Index ranges	$-47 \le h \le 47$<br>$-27 \le k \le 29$<br>$-24 \le l \le 30$	$-47 \le h \le 48$<br>$-27 \le k \le 29$<br>$-31 \le l \le 31$	$-47 \le h \le 47$<br>$-29 \le k \le 23$<br>$-28 \le l \le 31$	$-47 \le h \le 47$<br>$-29 \le k \le 29$<br>$-31 \le l \le 31$
Reflections collected	53679	100141	155424	299809
Independent reflections	21257 [R_{int} = 0.0332, R_{sigma} = 0.0491]	21929 [R_{int} = 0.0467, R_{sigma} = 0.0422]	43533 [R_{int} = 0.0526, R_{sigma} = 0.0580]	44898 [R_{int} = 0.0586, R_{sigma} = 0.0390]
Data/restraints/ parameters	21257/6/1191	21929/0/1191	43533/90/2392	44898/96/2392
GOF on F^2	1.044	1.037	1.027	1.022
R indexes [I>=2σ(I)]	R_1 = 0.0424, wR_2 = 0.1053	R_1 = 0.0405, wR_2 = 0.0988	R_1 = 0.0413, wR_2 = 0.0889	R_1 = 0.0411, wR_2 = 0.0922
R indexes [all data]	R_1 = 0.0603, wR_2 = 0.1137	R_1 = 0.0594, wR_2 = 0.1082	R_1 = 0.0626, wR_2 = 0.0953	R_1 = 0.0605, wR_2 = 0.1013
Largest diff. peak/hole, eA^{-3}	1.96/-1.27	2.32/-1.46	1.60/-1.41	1.32/-0.85

Table S3. Crystal data and structure refinement of **4*S*** and **4*R*** at different temperatures.

Compound	**4*S***		**4*R***	
CCDC	2190793	2190794	2190791	2190792
T/K	100	250	100	250
Empirical fromula	$C_{274}H_{296}Co_7N_{56}O_{26}W_4$	$C_{274}H_{296}Co_7N_{56}O_{26}W_4$	$C_{274}H_{296}Co_7N_{56}O_{26}W_4$	$C_{274}H_{296}Co_7N_{56}O_{26}W_4$
Formula weight	5937.56	5937.56	5937.56	5937.56
Crystal system	orthorhombic	orthorhombic	orthorhombic	orthorhombic
Space group	*I*222	*I*222	*I*222	*I*222
a/Å	21.9807(17)	22.0238(19)	21.2889(7)	21.9189(8)
b/Å	26.336(2)	26.367(2)	26.1033(9)	26.3920(10)
c/Å	29.298(2)	29.415(3)	29.3975(10)	29.4893(9)
α/°	90	90	90	90
β/°	90	90	90	90
γ/°	90	90	90	90
Volume/Å^3	16960(2)	17082(3)	16336.5(10)	17059.1(10)
Z	2	2	2	2
ρ_{calc}, g/cm^3	1.163	1.154	1.207	1.156
μ/mm^{-1}	1.742	1.730	1.809	1.732
F(000)	6050.0	6050.0	6050.0	6050.0
Radiation	MoKα (λ = 0.71073)	MoKα (λ = 0.71073)	MoKα (λ = 0.71073)	MoKα (λ = 0.71073)
2θ range/°	4.564 to 49.996	4.838 to 50	5.058 to 50.148	4.63 to 50.156
Index ranges	-26≤h≤26 -31≤k≤31 -34≤l≤34	-26≤h≤22 -31≤k≤31 -34≤l≤34	-21≤h≤25 -31≤k≤21 -35≤l≤31	-22≤h≤26 -31≤k≤23 -35≤l≤31
Reflections collected	67248	53397	44349	47757
Independent reflections	14908 [R_{int} = 0.0652, R_{sigma} = 0.0627]	14874 [R_{int} = 0.0337, R_{sigma} = 0.0397]	14393 [R_{int} = 0.0339, R_{sigma} = 0.0529]	15004 [R_{int} = 0.0350, R_{sigma} = 0.0530]
Data/restraints/parameters	14908/68/755	14874/591/825	14393/673/790	15004/625/803
Goodness-of-fit on F^2	1.045	1.026	1.037	1.044
R indexes [I>=2σ(I)]	R_1 = 0.0489, wR_2 = 0.1331	R_1 = 0.0434, wR_2 = 0.1079	R_1 = 0.0496, wR_2 = 0.1323	R_1 = 0.0474, wR_2 = 0.1258
R indexes [all data]	R_1 = 0.0576, wR_2 = 0.1380	R_1 = 0.0504, wR_2 = 0.1146	R_1 = 0.0580, wR_2 = 0.1383	R_1 = 0.0569, wR_2 = 0.1322
Largest diff. peak/hole, eA^{-3}	0.98/-0.8	1.19/-0.58	1.52/-0.60	1.76/-0.62
Flack parameter	0.039(4)	0.110(4)	0.070(5)	0.079(5)

Table S4. Results of Continuous Shape Measure Analysis for $[W(CN)_8]^{3-/4-}$ anions in **1-4**.

1

W	CShM values	100K		250K		Fe	100K	OC-6	Fe1	0.691
									Fe2	0.570
		W1	W2	W1	W2				Fe3	0.583
	BTPR-8	1.295	1.681	1.277	1.877			T-4	Fe4	1.635
	TDD-8	**1.009**	**0.595**	**1.063**	**0.589**		250K	OC-6	Fe1	1.151
	SAPR-8	1.329	1.558	1.231	1.415				Fe2	0.604
	JBTPR-8	2.002	2.382	1.999	2.440				Fe3	0.605
								T-4	Fe4	1.934

1^{de}

W	CShM values	100K		300K		Fe	100K	OC-6	Fe1	0.627
									Fe2	0.674
		W1	W2	W1	W2				Fe3	0.547
	BTPR-8	2.174	1.197	2.321	1.236			T-4	Fe4	1.679
	TDD-8	**0.486**	**1.033**	**0.454**	**0.589**		300K	OC-6	Fe1	1.010
	SAPR-8	2.315	1.333	2.412	1.174				Fe2	0.662
	JBTPR-8	2.811	1.921	2.882	1.947				Fe3	0.574
								T-4	Fe4	1.770

2

W	CShM	100K		250K		Co	100K	OC-6	Co1	1.488
									Co2	0.500
		W1	W2	W1	W2				Co3	0.513
	BTPR-8	1.852	1.335	1.865	1.388			T-4	Co4	2.994
	TDD-8	**0.654**	1.161	**0.642**	1.122		250K		Co1	1.514
	SAPR-8	1.747	**1.004**	1.725	**0.980**			OC-6	Co2	0.538
	JBTPR-8	2.424	1.935	2.451	2.031				Co3	0.528

3

W	CShM	100K				250K			
		W1	W2	W1A	W2A	W1	W2	W1A	W2A
	BTPR-8	2.024	1.403	1.771	1.304	2.057	1.416	1.815	1.320
	TDD-8	**0.385**	1.078	**0.652**	1.314	**0.387**	1.030	**0.638**	1.267
	SAPR-8	1.975	**0.960**	1.526	**0.939**	2.223	**0.980**	1.625	**0.919**
	JBTPR-8	2.608	2.028	2.382	1.953	2.624	2.010	2.395	1.980

			100K	250K	
Ni	OC-6	Ni1	1.207	1.148	
		Ni2	0.653	0.749	
		Ni3	0.795	0.797	
		Ni1A	1.100	1.128	
		Ni2A	0.741	0.743	
		Ni3A	0.810	0.796	

**4*S***								
W	CShM	100K	250K	Co	100K	OC-6	Co1	**1.344**
		W1	W1				Co2	**0.724**
	BTPR-8	1.267	1.188			T-4	Co3	**0.322**
	TDD-8	1.592	1.612		250K	OC-6	Co1	**1.495**
	SAPR-8	**0.729**	**0.788**				Co2	**0.744**
	JBTPR-8	1.977	1.869			T-4	Co3	**0.511**

**4*R***								
W	CShM	100K	250K	Co	100K	OC-6	Co1	**1.419**
		W1	W1				Co2	**0.866**
	BTPR-8	1.209	1.133			T-4	Co3	**0.435**
	TDD-8	1.737	1.611		250K	OC-6	Co1	**1.416**
	SAPR-8	**0.781**	**0.858**				Co2	**0.743**
	JBTPR-8	1.856	1.721			T-4	Co3	**0.498**

BTPR-8: Biaugmented trigonal prism
TDD-8: Triangular dodecahedron
SAPR-8: Square antiprism
JBTPR-8: Johnson- Biaugmented trigonal prism
OC-8: Octahedron
T-4: Tetrahedron

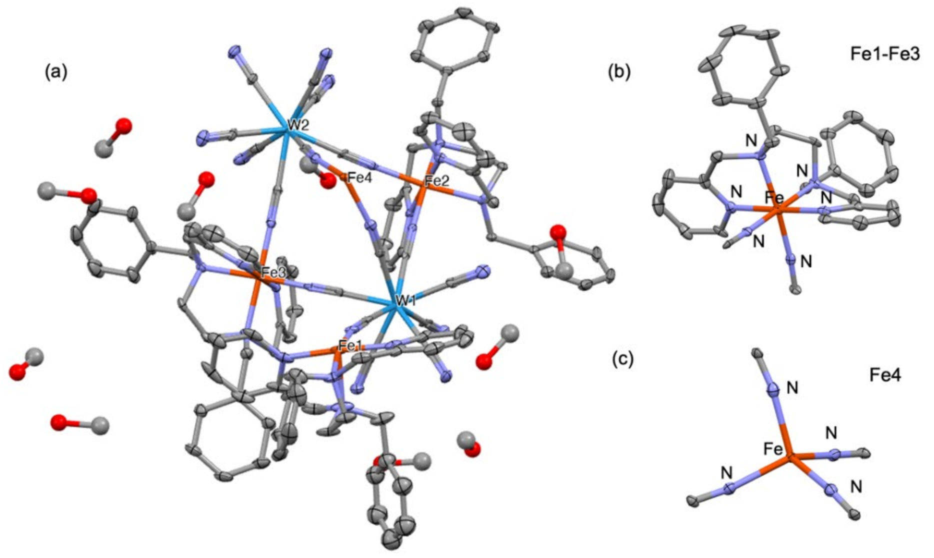

Figure S18 (a) Asymmetric unit of **1**$^{\mathbf{LT}}$. The representative coordination spheres for Fe1-Fe3 (b) and Fe4 (c) sites are given; these images are also representative for compounds **2** and **3**. Atom spheres are shown with 50% probability ellipsoids. Hydrogen atoms were omitted for clarity. Methanol molecules are shown in balls and sticks mode.

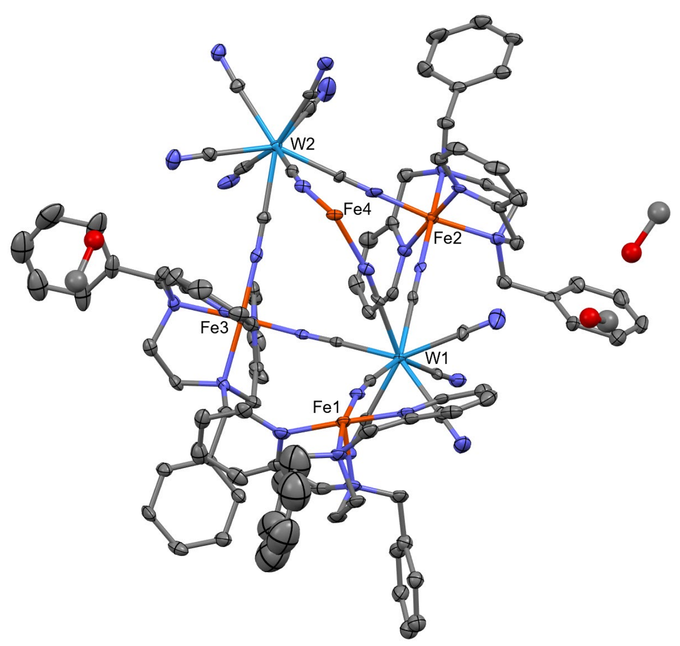

Figure S19 Asymmetric unit of **1**$^{\mathbf{de\ LT}}$. Atom spheres are shown with 50% probability ellipsoids. Hydrogen atoms were omitted for clarity. Methanol molecules are shown in balls and sticks mode.

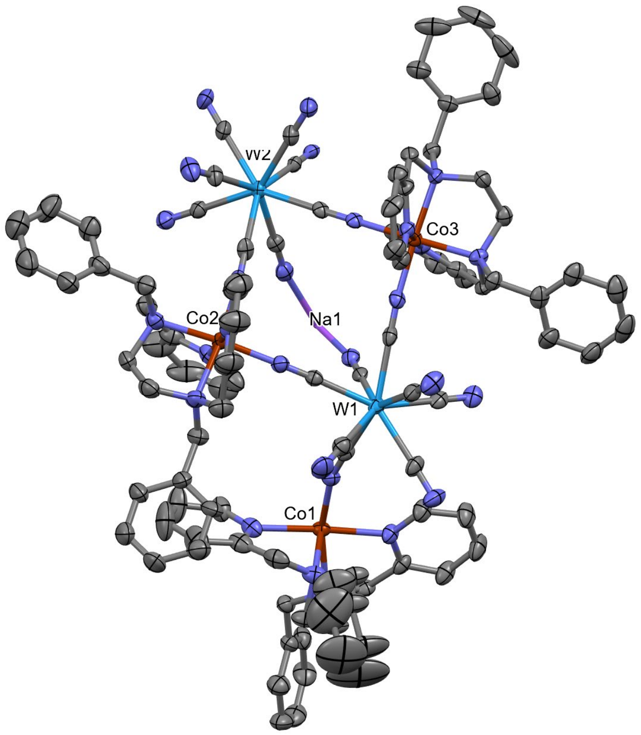

Figure S20 Asymmetric unit of 2^{LT}. Atom spheres are shown with 50% probability ellipsoids. Hydrogen atoms were omitted for clarity.

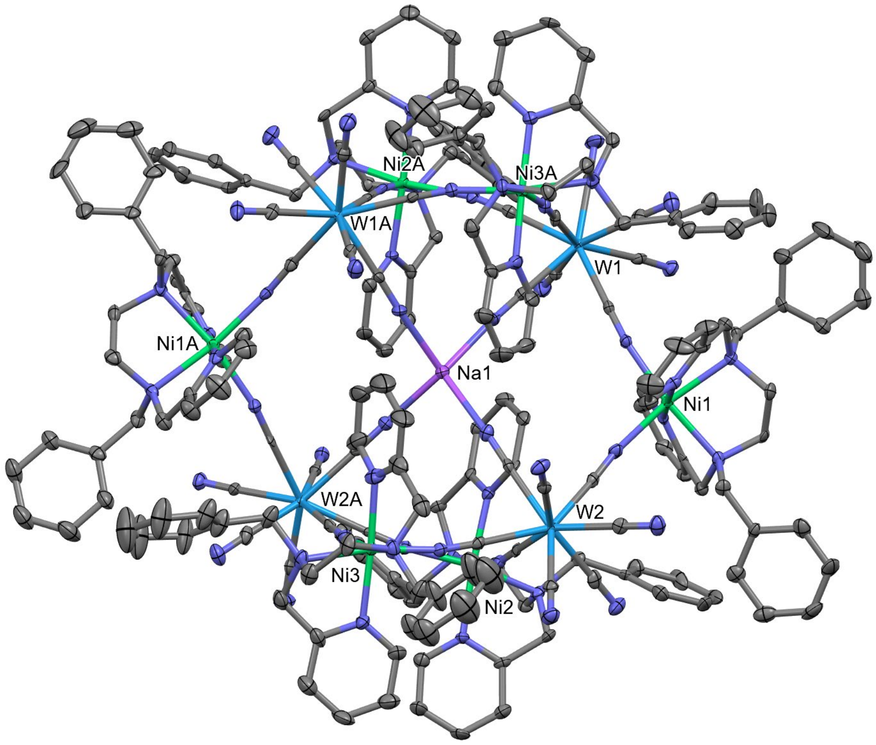

Figure S21. Asymmetric unit of 3^{LT}. Atom spheres are shown with 50% probability ellipsoids. Hydrogen atoms were omitted for clarity.

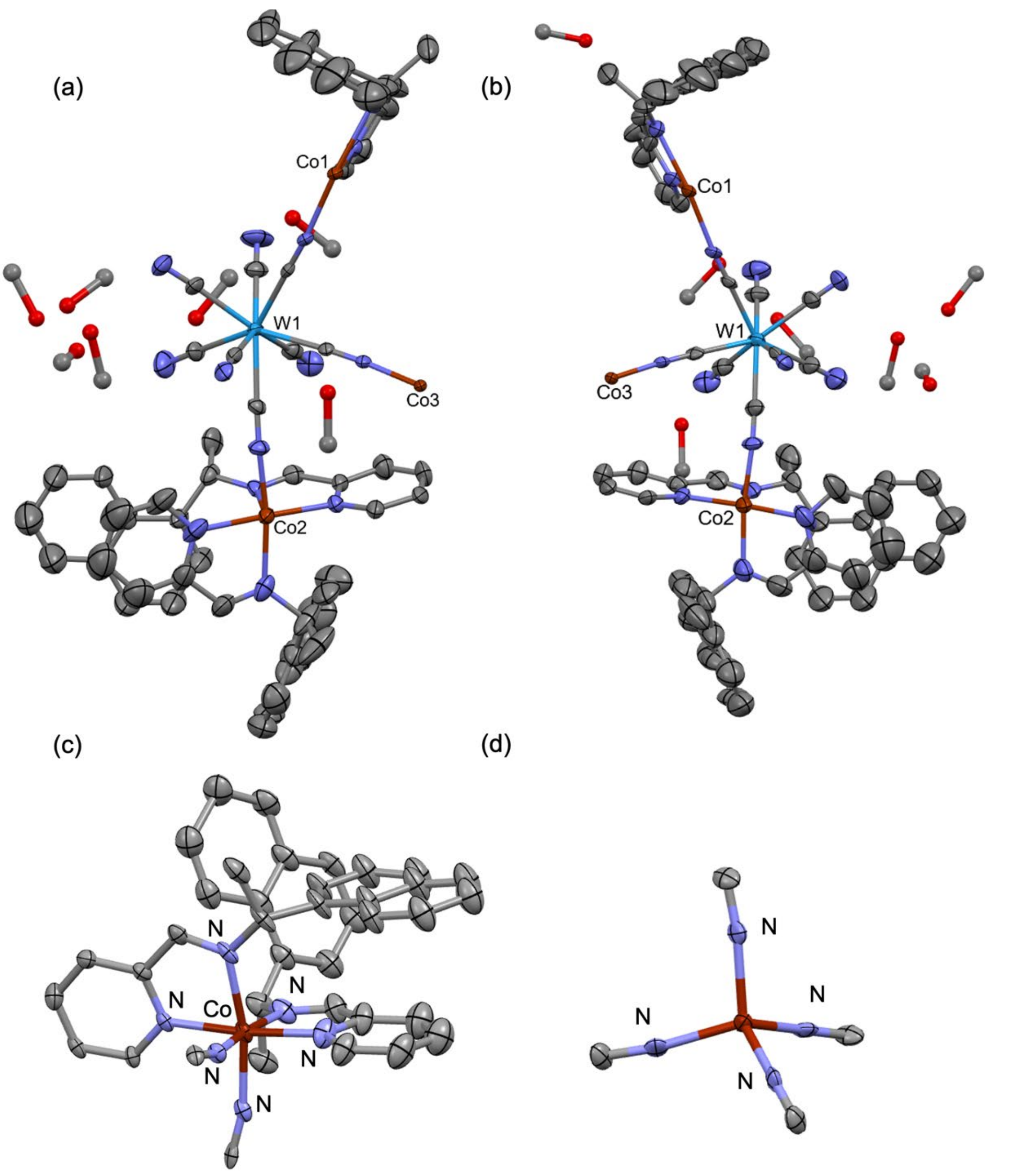

Figure S22. Asymmetric units of **4*S*^LT^** (a) and **4*R*^LT^** (b). The representative images of coordination spheres for Co1, Co2 (c) and Co3 (d) sites are given. Atom spheres are shown with 50% probability ellipsoids. Hydrogen atoms were omitted for clarity. Methanol molecules are shown in balls and sticks mode.

4. Supramolecular interactions and contacts.

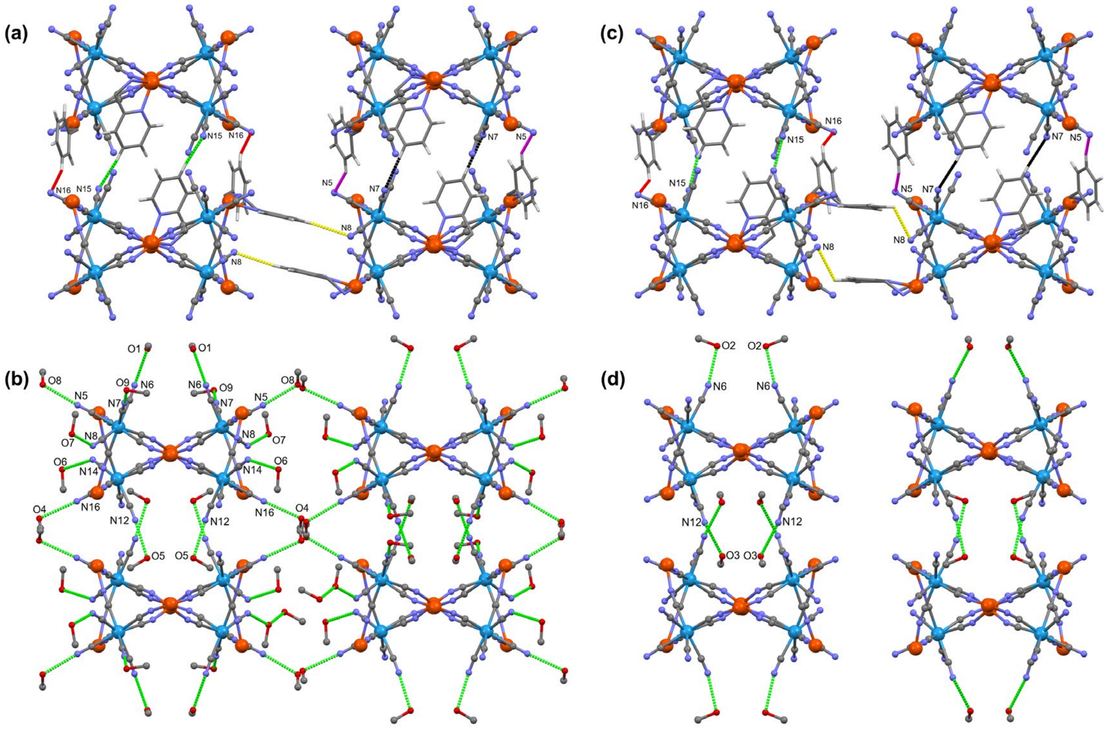

Figure S23. C_{phenyl}-H…N_{CN} contacts between the neighboring clusters in **1** (a) and $\mathbf{1^{de}}$ (c), and N_{CN}…H-O hydrogen bonds in **1** (b) and $\mathbf{1^{de}}$ (d). Projection along the crystallographic *c* directions. These contacts parameters are summarized in **Table S5** and **Table S6** (*see below*).

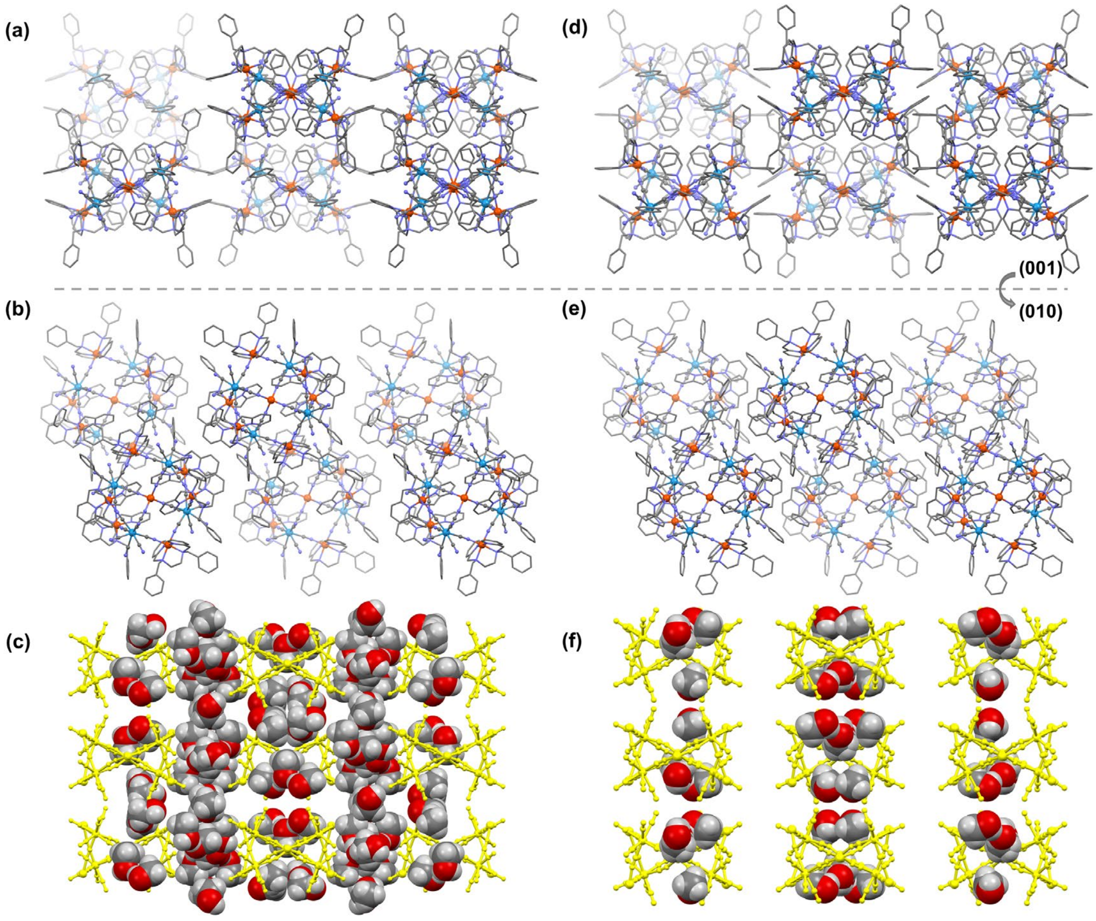

Figure S24. Crystal packing in **1** (a-c) and $\mathbf{1^{de}}$ (d-f). Projections along the crystallographic directions *c* (a, d) and *b* (b, e). Methanol molecules and H atoms are omitted for clarity. (c) Space-filling views of the methanol molecules are shown in the solvent accessible space (projection along the *c* direction).

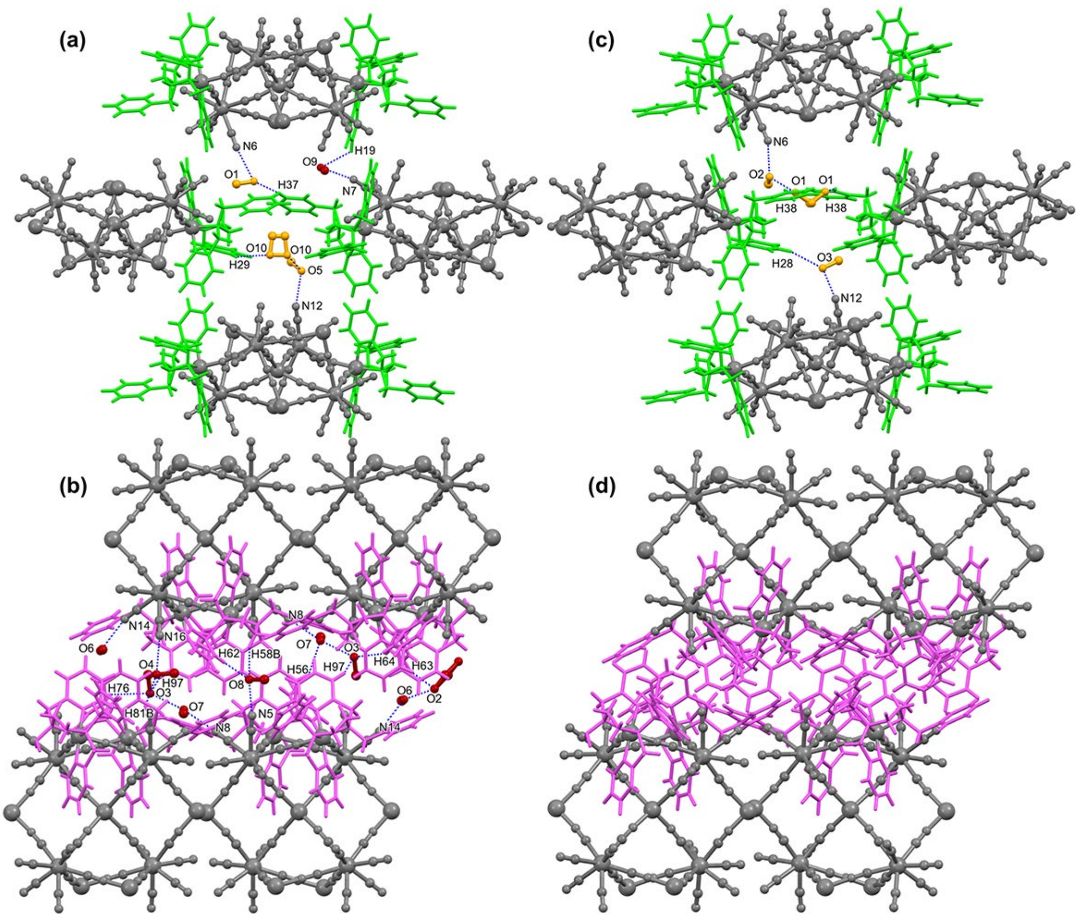

Figure S25. The projection of crystal packing of **1** and **1de** along the crystallographic directions *a*, (a) and (c), respectively, and *b*, (b) and (d), respectively. The MeOH molecules are distributed within two distinguished solvent accessible spaces in **1** and **1de** at 100 K. (a) One third of MeOH molecules in **1** trapped in the intralayer cages adjacent to the *vertex* parts of the cluster; (b) Two thirds of MeOH molecules in **1** located in the one-dimensional channels weaving along the *c* direction; (c) MeOH molecules in **1de** trapped in the intralayer cages adjacent to the *vertex* parts of the cluster; (d) Solvent free one-dimensional channels in **1de**. Color code: grey – cluster core, green – organic ligands attached to the vertex metal ions, violet – organic ligands attached to the lateral metal ions, red – MeOH molecules removed during the **1** → **1de** process, yellow – MeOH molecules that are retained during the **1** → **1de** process. Hydrogen bond parameters are summarized in **Table S6** (*see below*).

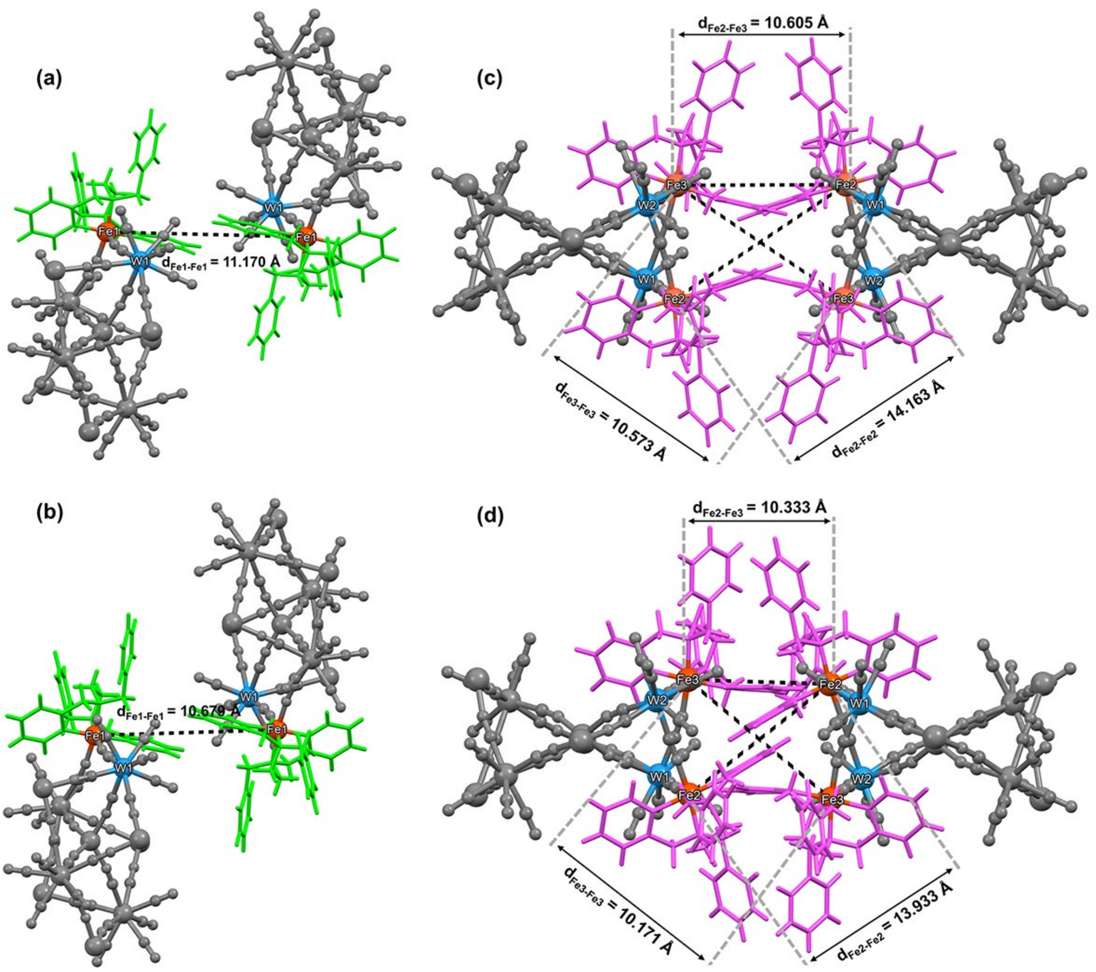

Figure S26. The selected intercluster separation distances in **1** (a, c) and $\mathbf{1^{de}}$ (b, d). Projection along the crystallographic directions *a* (a, b) and *c* (c, d). During desolvation noteworthy modification of the mutual phenyl ring orientation occurs in the space between the clusters (c, d). Color code: grey – cluster core, green – organic ligands attached to the vertex metal ions, violet – organic ligands attached to the lateral metal ions.

Table S5. Weak N_{CN}…H-C_{phenyl} supramolecular contacts in **1** and $\mathbf{1^{de}}$ at 100 K.

**1**			
N_{CN}…H-C_{phenyl}	D…A (Å)	H…A (Å)	D-H…A (deg)
N8…H55-C55	3.739	2.812	169.31
N15…H42-C42	3.352	2.460	158.25
N16…H48-C48	3.471	2.678	142.48
N7…H19-C19	3.373	2.441	172.15
N5…H85-C85	3.434	2.673	138.49
$\mathbf{1^{de}}$			
N_{CN}…H-C_{phenyl}	D…A (Å)	D…A (Å)	D…A (Å)
N8…H55-C55	3.315	2.882	108.93
N15…H42-C42	3.278	2.350	165.87
N16…H46-C46	3.363	2.772	121.25
N7…H19-C19	3.465	2.566	158.00
N5…H85-C85	3.516	2.754	137.79

Table S6. The most important hydrogen bonds and intermolecular contacts involving methanol molecules in the space close to the cluster vertex region and lateral region for **1** and **1**^{de} at 100 K (Å, deg)

**1**				
		D…A (Å)	H…A (Å)	D-H…A (deg)
Vertex	O1-H1…N6	2.969	2.209	153.86
	O5-H5…O10	2.828	2.006	179.29
	O9-H9…N7	2.885	2.067	170.16
	O9…H19-C19	3.221	2.659	119.04
	O1…H37-C37	3.554	2.618	172.47
	O10…H29-C29	3.471	2.730	136.05
Lateral	O2-H2…O6	2.665 Å	2.054	130.24
	O3-H3…O7	2.749 Å	1.923	172.60
	O6-H6…N14	2.853 Å	2.171	139.48
	O4-H4…N16	2.968 Å	2.209	152.51
	O7-H7…N8	2.798 Å	1.798	168.84
	O8-H8…N5	2.905 Å	2.087	167.01
	O3…H76-C76	3.690	2.989	132.51
	O3…H97-C97	3.143	2.538	122.23
	O3…H64-C64	3.361	2.616	136.68
	O4…H81B-C81	3.422	2.766	124.89
	O8…H58B-C58	3.188	2.290	151.95
	O8…H62	3.713	2.790	167.19
	O7…H56	3.253	2.523	134.74
	O2…H63	3.647	2.764	157.03
**1**^{de}				
		D…A (Å)	H…A (Å)	D-H…A (°)
Vertex	O1…H38-C38	3.187	2.346	147.65
	O2-H2…O1	2.523	1.726	159.09
	O2…N6	2.827	-	-
	O3…N12	2.997	-	-
	O3…H28-C28	3.712	2.789	163.65

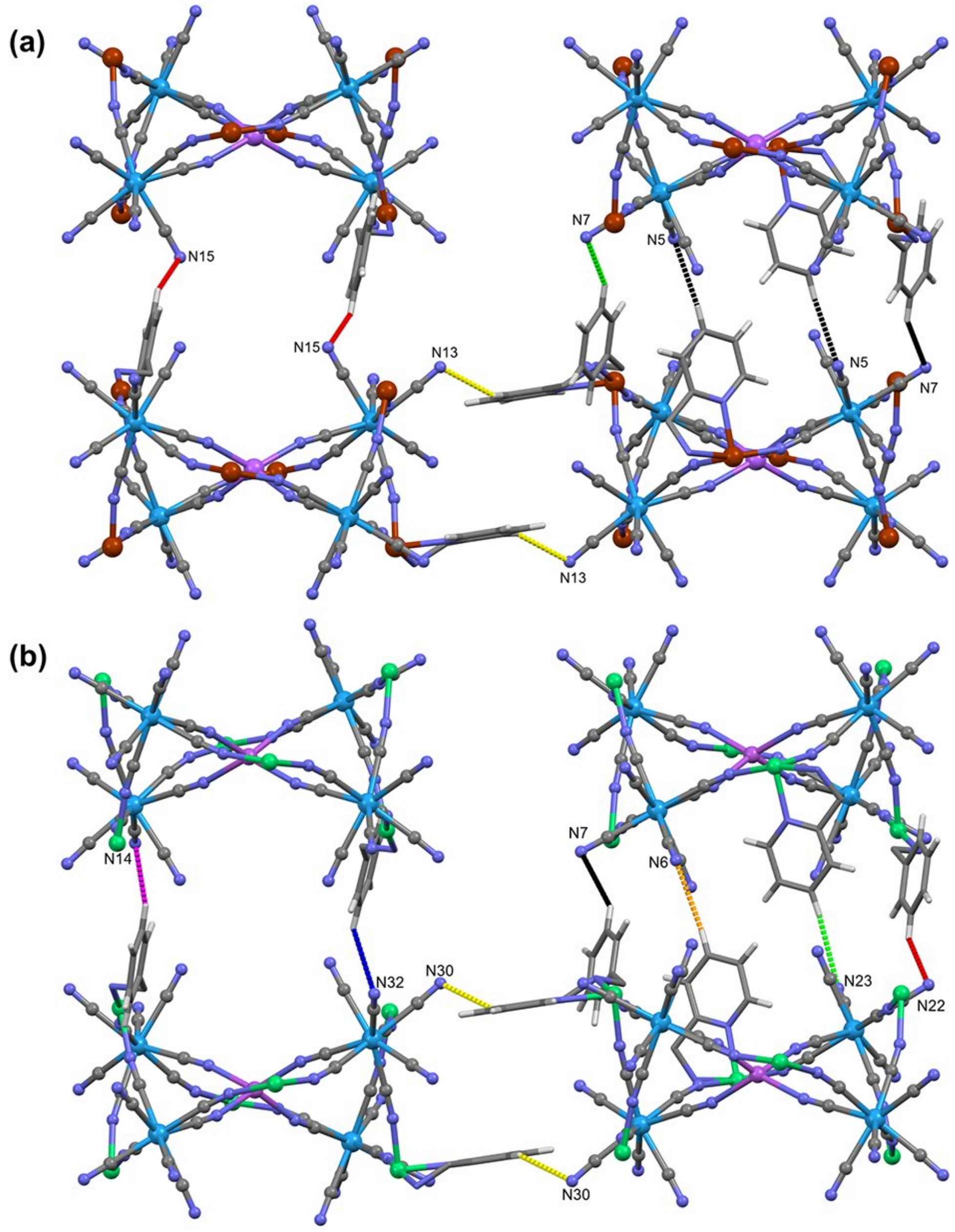

Figure S27. C_{phenyl}-H…N_{CN} contacts between the neighboring clusters in **2** (a) and **3** (b). Projection along the crystallographic *c* directions. These contacts parameters are summarized in **Table S7** (*see below*).

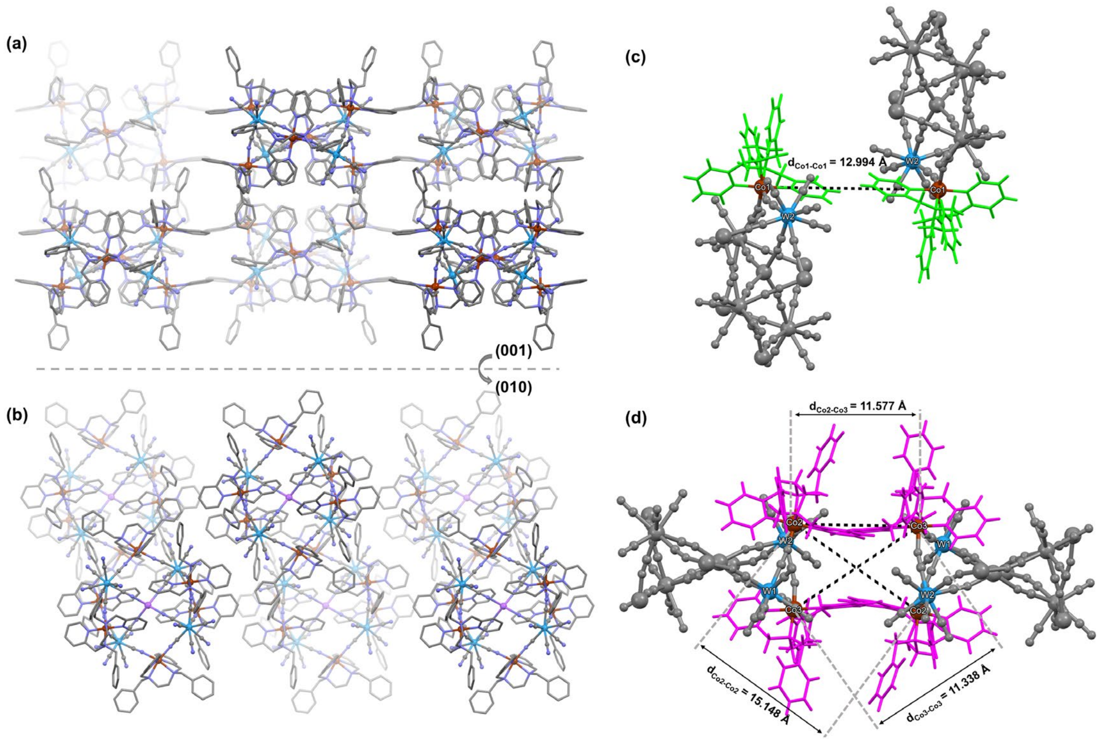

Figure S28. Crystal packing in **2** (a-b). Projections along the crystallographic directions *c* (a) and *b* (b); The selected intercluster separation distances in **2**(c-d). Projection along the crystallographic directions *a* (c) and *c* (d). Color code in (c) and (d): grey – cluster core, green – organic ligands attached to the vertex metal ions, violet – organic ligands attached to the lateral metal ions.

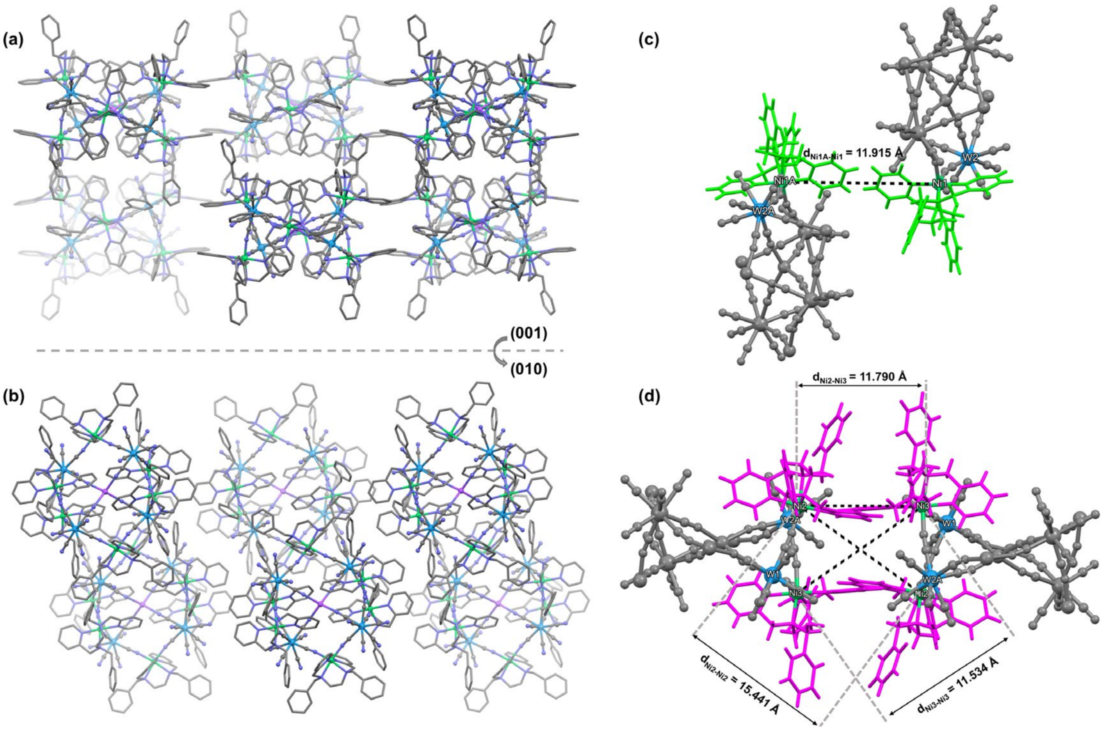

Figure S29. Crystal packing in **3** (a-b). Projections along the crystallographic directions *c* (a) and *b* (b); The selected intercluster separation distances in **3**(c-d). Projection along the crystallographic directions *a* (c) and *c* (d).

Table S7. Weak N_{CN}…H-C_{phenyl} supramolecular contacts in **2** and **3** at 100 K.

**2**			
N_{CN}…H-C_{phenyl}	D…A (Å)	H…A (Å)	D-H…A (˚)
N15…H63-C63	3.455	2.737	132.93
N7…H82-C82	3.452	2.652	142.34
N5…H19-C19	3.691	2.759	167.13
N13…H76-C76	3.526	2.693	146.74
**3**			
N_{CN}…H-C_{phenyl}	D…A (Å)	H…A (Å)	D-H…A (Å)
N14…H184-C184	3.366	2.567	141.79
N32…H114-C114	3.500	2.777	133.40
N7…H95-C95	3.572	2.744	145.86
N6…H65-C65	3.780	2.858	164.15
N23…H52-C52	3.838	2.892	174.90
N22…H148-C148	3.508	2.756	136.71
N30…H101-C101	3.489	2.664	145.664

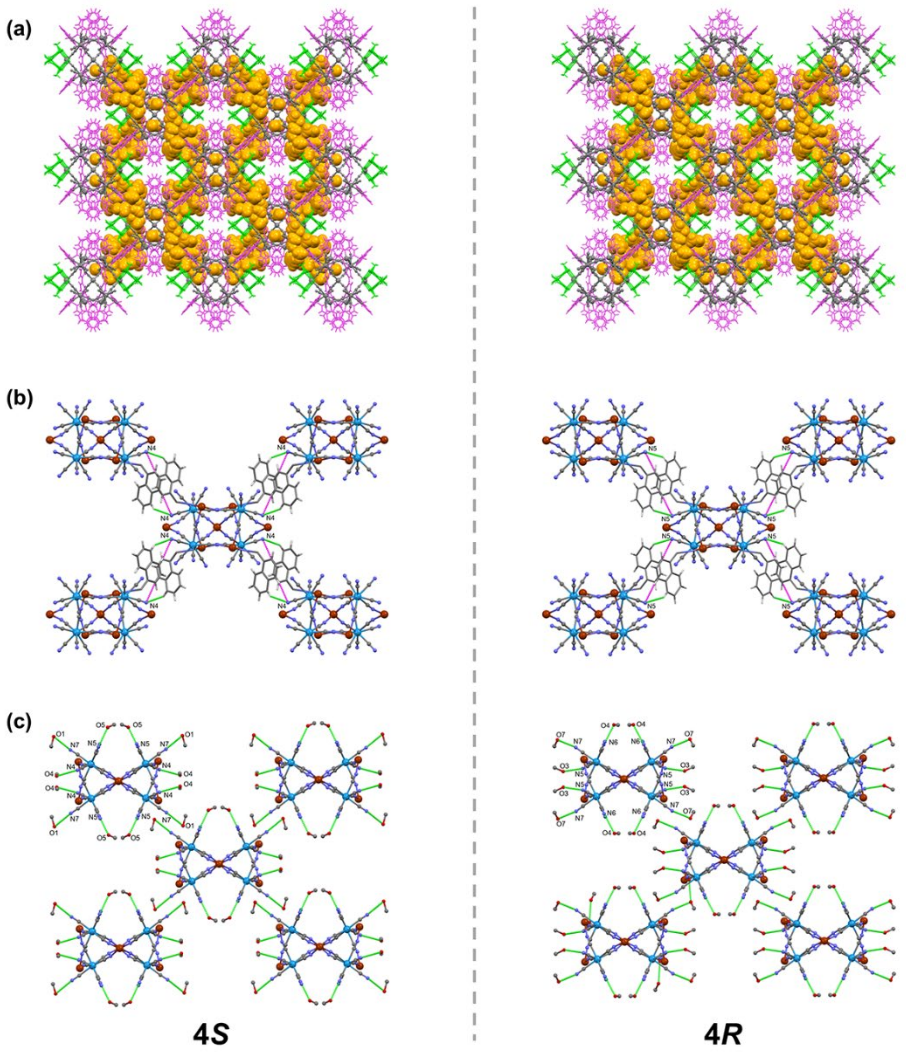

Figure S30. (a) Supramolecular architecture of **4*S*** and **4*R*** within the *bc* plane; (b) Views of C_{phenyl}-H…N_{CN} contacts of **4*S*** and **4*R*** shown along the crystallographic *b* directions; (c) N_{CN}…H-O hydrogen bonds along the crystallographic *c* directions. These contacts parameters are summarized in **Table S8**.

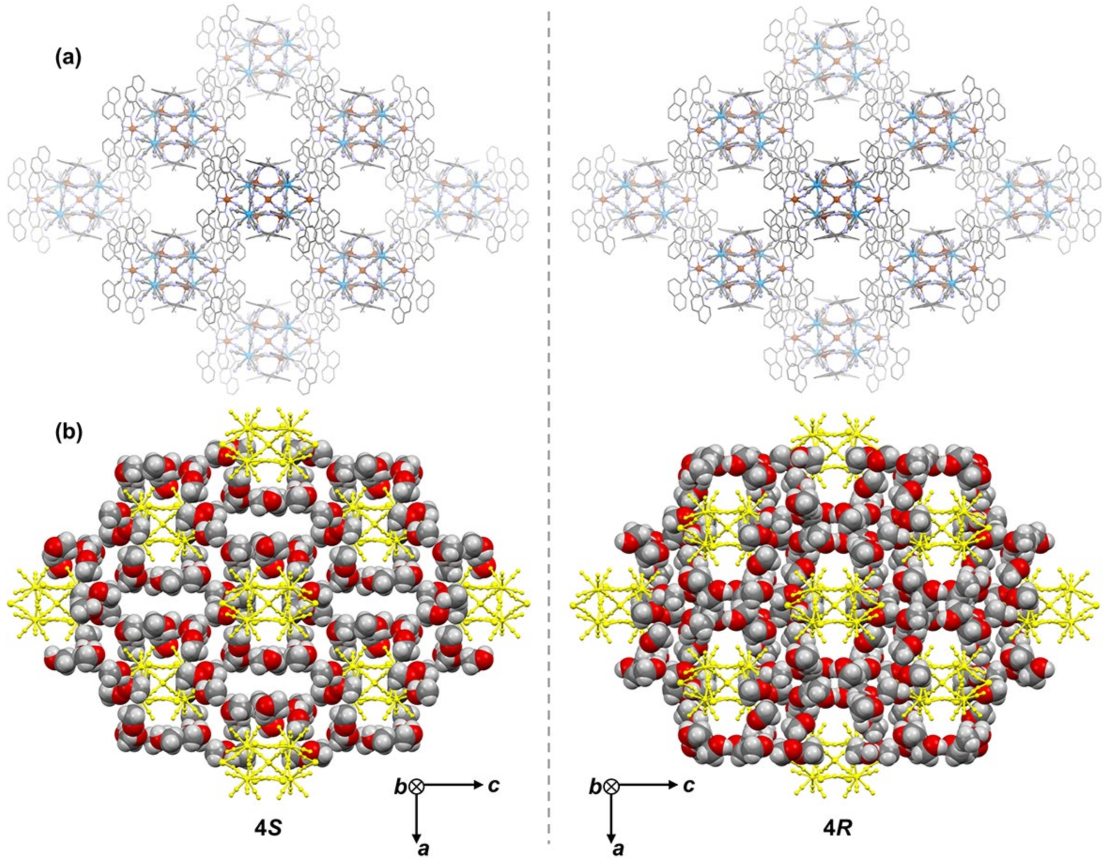

Figure S31. (a) Crystal packing in **4*S*** and **4*R***. Projections along the crystallographic cell directions *b*. Methanol molecules and H atoms are omitted for clarity. (b) Space-filling views of the methanol molecules are shown in the intercluster voids.

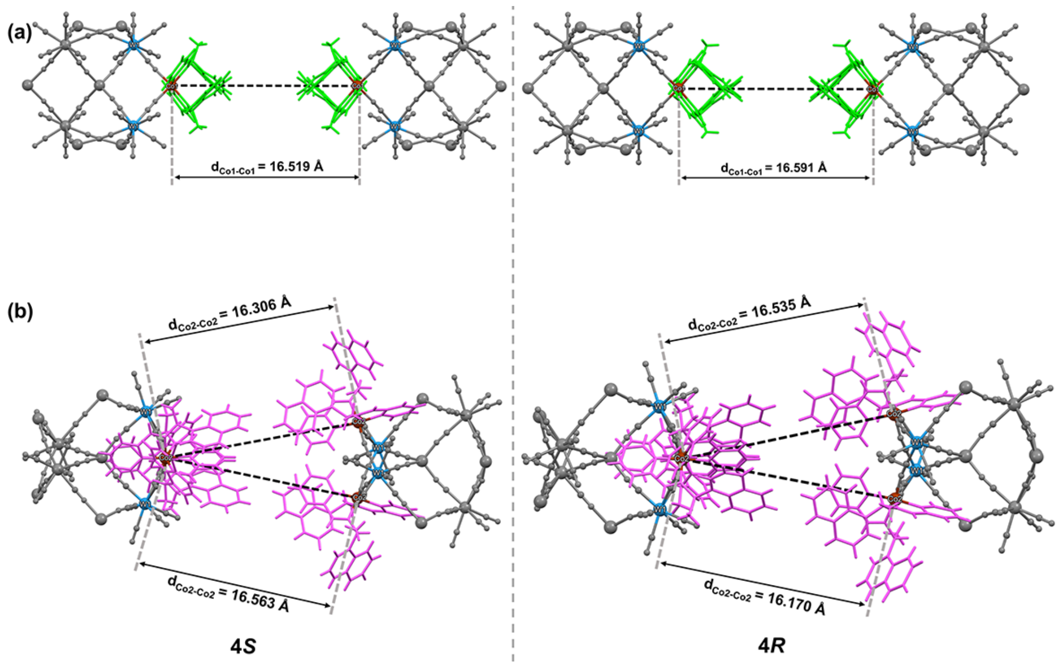

Figure S32. The selected intercluster separation distances in **4*S*** (a, c) and **4*R*** (b, d). Projection along the crystallographic directions *a* (a, b) and *c* (c, d).

Table S8. Hydrogen-bonding and weak intermolecular interactions or contacts in **4*S*** and **4*R*** at 100 K.

**4*S***			
N_{CN}…H-C_{phenyl}	D…A (Å)	H…A (Å)	D-H…A (˚)
N4…H56-C56	3.590	2.700	148.96
N4…H58-C58	3.626	2.779	156.38
N7…H1-O1	3.010	2.460	123.99
N4…H4-O4	2.975	2.347	134.04
N5…H5-O5	2.829	1.994	171.14
**4*R***			
	D…A (Å)	H…A (Å)	D-H…A (Å)
N5…H44-C44	3.622	2.778	148.56
N5…H38-C38	3.422	2.516	159.20
N7…H7-O7	2.783	1.998	162.39
N6…H4-O4	2.902	2.117	165.96
N5…H3-O3	3.260	2.627	136.65

5. Bond length and angles parameters.

Table S9. Detailed structure bond lengths and bond angles of Fe^{II} ions for **1** and $\mathbf{1^{de}}$ at low and high temperature phase.

	**1**			$\mathbf{1^{de}}$	
	**100K**	**250K**		**100K**	**300K**
Fe1-N1	1.974(6)	2.077(6)	Fe1-N1	1.931(6)	2.048(7)
Fe1-N19	2.016(7)	2.155(7)	Fe1-N19	1.995(6)	2.155(8)
Fe1-N20	1.998(7)	2.164(7)	Fe1-N20	1.986(6)	2.125(7)
Fe1-N18	2.109(6)	2.275(6)	Fe1-N18	2.069(6)	2.222(7)
Fe1-N17	2.104(6)	2.250(5)	Fe1-N17	2.085(6)	2.249(6)
Fe1-$N13^1$	1.949(6)	2.087(6)	Fe1-$N13^1$	1.935(6)	2.062(7)
Fe2-N2	1.916(5)	1.915(5)	Fe2-N2	1.904(6)	1.905(6)
Fe2-N24	1.969(6)	1.975(5)	Fe2-N10	1.915(6)	1.907(7)
Fe2-N10	1.902(6)	1.909(6)	Fe2-N25	1.992(6)	1.994(6)
Fe2-N22	2.050(5)	2.054(5)	Fe2-N26	2.060(6)	2.053(6)
Fe2-N23	2.042(5)	2.048(5)	Fe2-N28	1.972(6)	1.965(6)
Fe2-N21	1.986(5)	1.986(5)	Fe2-N27	2.056(6)	2.052(6)
Fe3-N27	1.970(5)	1.973(5)	Fe3-N24	1.959(6)	1.966(6)
Fe3-N3	1.916(6)	1.925(6)	Fe3-N3	1.921(7)	1.941(7)
Fe3-N11	1.911(6)	1.905(5)	Fe3-N11	1.920(6)	1.898(6)
Fe3-N25	1.985(6)	1.992(5)	Fe3-N21	1.984(6)	1.991(6)
Fe3-N26	2.048(5)	2.049(5)	Fe3-N23	2.047(6)	2.056(6)
Fe3-N28	2.062(6)	2.058(5)	Fe3-N22	2.070(6)	2.084(6)
Fe4-N4	2.012(6)	2.028(5)	Fe4-N4	2.028(6)	2.024(6)
Fe4-$N4^1$	2.012(6)	2.028(5)	Fe4-$N4^1$	2.028(6)	2.024(6)
Fe4-N9	2.034(6)	2.047(6)	Fe4-N9	2.014(6)	2.022(7)
Fe4-$N9^1$	2.034(6)	2.047(6)	Fe4-$N9^1$	2.014(6)	2.022(7)
N1-Fe1-N19	92.0(2)	93.9(3)	N1-Fe1-N19	92.1(2)	94.2(3)
N1-Fe1-N20	89.2(2)	91.8(2)	N1-Fe1-N20	88.7(2)	90.8(3)
N1-Fe1-N18	169.6(2)	166.5(2)	N1-Fe1-N18	171.8(3)	167.9(3)
N1-Fe1-N17	89.7(2)	91.6(2)	N1-Fe1-N17	90.9(2)	91.7(2)
N19-Fe1-N18	80.1(3)	76.3(3)	N1-Fe1-$N13^1$	94.2(2)	97.1(3)
N19-Fe1-N17	97.6(3)	99.3(2)	N19-Fe1-N18	80.8(2)	76.4(3)
N20-Fe1-N19	177.6(2)	173.0(2)	N19-Fe1-N17	97.8(2)	97.3(3)
N20-Fe1-N18	98.4(2)	97.4(2)	N20-Fe1-N19	178.1(2)	172.0(3)
N20-Fe1-N17	80.3(3)	76.5(2)	N20-Fe1-N18	98.3(2)	97.8(3)
N17-Fe1-N18	84.7(2)	80.9(2)	N20-Fe1-N17	80.5(2)	76.3(3)
$N13^1$-Fe1-N1	96.2(2)	98.8(2)	N18-Fe1-N17	86.0(2)	82.1(2)
$N13^1$-Fe1-N19	89.6(2)	89.9(2)	$N13^1$-Fe1-N19	89.5(2)	90.9(3)
$N13^1$-Fe1-N20	92.3(2)	93.2(2)	$N13^1$-Fe1-N20	92.2(3)	94.6(3)
$N13^1$-Fe1-N18	90.6(2)	90.6(2)	$N13^1$-Fe1-N18	89.9(2)	90.7(3)

N13[1]-Fe1-N17	170.6(3)	165.6(2)	N13[1]-Fe1-N17	170.9(2)	167.5(3)
N2-Fe2-N24	92.2(2)	92.3(2)	N2-Fe2-N10	91.2(2)	90.7(2)
N2-Fe2-N22	90.8(2)	90.6(2)	N2-Fe2-N25	85.6(2)	85.6(2)
N2-Fe2-N23	173.0(2)	172.7(2)	N2-Fe2-N26	91.6(2)	91.3(2)
N2-Fe2-N21	87.4(2)	87.5(2)	N2-Fe2-N28	91.2(2)	91.5(2)
N24-Fe2-N22	97.6(2)	97.8(2)	N2-Fe2-N27	173.0(2)	173.1(2)
N24-Fe2-N23	81.6(2)	81.3(2)	N10-Fe2-N25	92.0(2)	92.7(3)
N24-Fe2-N21	178.6(2)	178.6(2)	N10-Fe2-N26	172.9(2)	173.7(2)
N10-Fe2-N2	91.1(2)	90.8(2)	N10-Fe2-N28	88.6(2)	87.9(2)
N10-Fe2-N24	89.7(2)	89.7(2)	N10-Fe2-N27	90.6(2)	91.6(2)
N10-Fe2-N22	172.4(2)	172.3(2)	N25-Fe2-N26	81.8(2)	81.5(2)
N10-Fe2-N23	92.2(2)	92.5(2)	N25-Fe2-N27	101.1(2)	100.8(2)
N10-Fe2-N21	91.7(2)	91.7(2)	N28-Fe2-N25	176.8(2)	177.0(2)
N23-Fe2-N22	86.8(2)	86.9(2)	N28-Fe2-N26	97.8(2)	98.0(2)
N21-Fe2-N22	81.1(2)	80.8(2)	N28-Fe2-N27	82.0(2)	82.1(3)
N21-Fe2-N23	98.7(2)	98.9(2)	N27-Fe2-N26	87.3(2)	87.1(2)
N27-Fe3-N25	177.9(2)	178.1(2)	N24-Fe3-N21	176.5(2)	176.9(3)
N27-Fe3-N26	81.7(2)	81.5(2)	N24-Fe3-N23	81.7(2)	80.8(3)
N27-Fe3-N28	97.1(2)	97.2(2)	N24-Fe3-N22	95.7(2)	95.6(2)
N3-Fe3-N27	89.8(2)	89.7(2)	N3-Fe3-N24	92.5(2)	92.0(3)
N3-Fe3-N25	92.0(2)	91.9(2)	N3-Fe3-N21	91.0(2)	91.0(3)
N3-Fe3-N26	91.3(2)	92.2(2)	N3-Fe3-N23	92.6(2)	93.1(2)
N3-Fe3-N28	172.2(2)	172.5(2)	N3-Fe3-N22	171.5(2)	172.1(3)
N11-Fe3-N27	92.7(2)	93.2(2)	N11-Fe3-N24	91.6(2)	92.4(3)
N11-Fe3-N3	91.1(2)	90.6(2)	N11-Fe3-N3	91.4(2)	90.5(2)
N11-Fe3-N25	86.4(2)	85.8(2)	N11-Fe3-N21	88.0(2)	88.2(3)
N11-Fe3-N26	173.9(2)	174.0(2)	N11-Fe3-N23	172.3(2)	172.4(3)
N11-Fe3-N28	91.9(2)	91.9(2)	N11-Fe3-N22	90.7(2)	91.3(2)
N25-Fe3-N26	99.1(2)	99.4(2)	N21-Fe3-N23	98.4(2)	98.4(3)
N25-Fe3-N28	81.1(2)	81.3(2)	N21-Fe3-N22	80.8(2)	81.3(3)
N26-Fe3-N28	86.3(2)	86.0(2)	N23-Fe3-N22	86.3(2)	86.0(2)
N4-Fe4-N4[1]	128.0(3)	128.5(3)	N4-Fe4-N4[1]	124.8(3)	126.3(4)
N4[1]-Fe4-N9[1]	101.4(2)	98.6(2)	N9-Fe4-N4[1]	101.0(2)	103.5(2)
N4-Fe4-N9	101.4(2)	98.6(2)	N9[1]-Fe4-N4	101.0(2)	103.6(2)
N4-Fe4-N9[1]	104.0(2)	106.1(2)	N9[1]-Fe4-N4[1]	104.2(2)	101.3(2)
N4[1]-Fe4-N9	104.0(2)	106.1(2)	N9-Fe4-N4	104.2(2)	101.3(2)
N9-Fe4-N9[1]	119.9(3)	121.1(3)	N9[1]-Fe4-N9	123.6(3)	123.0(4)

[1]1-X, +Y, 1/2-Z

Table S10. Detailed structure bond lengths and bond angles of Co^{II} ions for **2** and Ni ions for **3** at low and high temperatures.

	**2**			**3**	
	**100K**	**250K**		**100K**	**250K**
Co1-N1	2.093(4)	2.102(4)	Ni1-N56	2.096(4)	2.095(4)
Co1-N17	2.149(5)	2.173(4)	Ni1-N1	2.090(4)	2.098(4)
Co1-N18	2.133(4)	2.146(4)	Ni1-N9	2.065(4)	2.089(4)
Co1-N19	2.238(4)	2.258(4)	Ni1-N54	2.166(4)	2.176(4)
Co1-N20	2.256(5)	2.253(5)	Ni1-N53	2.090(4)	2.102(4)
Co1-N16^1	2.116(4)	2.115(4)	Ni1-N55	2.176(4)	2.168(4)
Co2-N9	1.967(5)	1.968(5)	Ni1A-N27	2.105(4)	2.105(4)
Co2-N2	1.960(5)	1.977(5)	Ni1A-N19	2.115(5)	2.112(4)
Co2-N22	2.012(4)	2.006(5)	Ni1A-N44	2.097(4)	2.108(4)
Co2-N21	2.013(5)	2.020(5)	Ni1A-N42	2.182(4)	2.178(4)
Co2-N24	2.070(5)	2.089(4)	Ni1A-N41	2.165(4)	2.176(4)
Co2-N23	2.081(5)	2.102(5)	Ni1A-N43	2.083(4)	2.085(4)
Co3-N10	1.922(4)	1.932(4)	Ni2-N10	2.043(4)	2.042(4)
Co3-N25	1.982(4)	1.974(4)	Ni2-N17	2.054(4)	2.055(4)
Co3-N28	1.978(4)	1.961(4)	Ni2-N50	2.083(4)	2.088(4)
Co3-N3	1.912(5)	1.925(5)	Ni2-N49	2.091(4)	2.094(4)
Co3-N26	2.030(4)	2.038(4)	Ni2-N52	2.152(4)	2.158(4)
Co3-N27	2.018(4)	2.033(4)	Ni2-N51	2.148(4)	2.144(4)
N1-Co1-N17	90.16(17)	90.74(17)	Ni2A-N34	2.084(4)	2.077(4)
N1-Co1-N18	91.07(17)	90.94(17)	Ni2A-N33	2.086(4)	2.099(4)
N1-Co1-N19	163.04(17)	162.68(16)	Ni2A-N26	2.041(5)	2.050(4)
N1-Co1-N20	88.18(17)	88.75(17)	Ni2A-N2	2.062(4)	2.058(4)
N1-Co1-N16^1	105.28(16)	105.21(17)	Ni2A-N36	2.164(4)	2.171(4)
N17-Co1-N19	100.18(17)	99.98(17)	Ni2A-N35	2.147(4)	2.149(4)
N17-Co1-N20	77.29(19)	76.90(18)	Ni3-N18	2.047(5)	2.054(5)
N18-Co1-N17	176.72(18)	176.06(17)	Ni3-N11	2.035(4)	2.045(4)
N18-Co1-N19	77.93(17)	77.55(16)	Ni3-N46	2.087(4)	2.077(4)
N18-Co1-N20	99.71(18)	99.58(17)	Ni3-N45	2.151(4)	2.162(4)
N19-Co1-N20	81.12(16)	80.58(17)	Ni3-N47	2.086(5)	2.090(4)
N16^1-Co1-N17	91.09(17)	91.09(16)	Ni3-N48	2.158(5)	2.158(4)
N16^1-Co1-N18	91.52(16)	91.90(16)	Ni3A-N25	2.050(4)	2.056(4)
N16^1-Co1-N19	88.06(15)	88.28(16)	Ni3A-N4	2.043(5)	2.043(4)
N16^1-Co1-N20	162.37(17)	161.83(16)	Ni3A-N39	2.079(4)	2.086(4)
N9-Co2-N22	88.31(19)	88.68(19)	Ni3A-N38	2.142(4)	2.131(5)
N9-Co2-N21	94.42(19)	94.52(18)	Ni3A-N40	2.079(4)	2.067(4)
N9-Co2-N24	172.4(2)	171.59(19)	Ni3A-N37	2.161(4)	2.167(4)
N9-Co2-N23	89.47(18)	89.53(17)	N56-Ni1-N54	101.64(16)	101.03(16)
N2-Co2-N9	92.69(19)	92.71(18)	N56-Ni1-N55	78.25(15)	78.55(17)

N2-Co2-N22	91.30(19)	90.95(19)	N1-Ni1-N56	87.91(16)	89.10(17)
N2-Co2-N21	90.14(18)	90.20(19)	N1-Ni1-N54	166.37(15)	166.11(16)
N2-Co2-N24	93.01(18)	93.72(18)	N1-Ni1-N53	90.52(16)	90.27(17)
N2-Co2-N23	172.66(18)	172.53(19)	N1-Ni1-N55	89.63(15)	90.02(16)
N22-Co2-N21	176.85(19)	176.54(19)	N9-Ni1-N56	91.62(16)	91.64(17)
N22-Co2-N24	96.57(19)	96.64(19)	N9-Ni1-N1	100.40(15)	100.17(16)
N22-Co2-N23	81.75(18)	81.96(18)	N9-Ni1-N54	89.15(15)	89.12(16)
N21-Co2-N24	80.56(19)	80.03(19)	N9-Ni1-N53	91.04(16)	91.28(16)
N21-Co2-N23	96.69(18)	96.73(18)	N9-Ni1-N55	165.53(16)	165.79(16)
N24-Co2-N23	85.50(18)	84.78(17)	N54-Ni1-N55	82.92(15)	82.79(15)
N10-Co3-N25	90.19(17)	90.68(17)	N53-Ni1-N56	177.12(15)	177.08(16)
N10-Co3-N28	88.75(17)	88.57(17)	N53-Ni1-N54	79.50(16)	79.14(16)
N10-Co3-N26	172.44(17)	172.65(17)	N53-Ni1-N55	99.33(15)	98.61(16)
N10-Co3-N27	92.01(17)	92.09(17)	N27-Ni1A-N19	100.58(15)	100.49(16)
N25-Co3-N26	82.49(17)	82.22(16)	N27-Ni1A-N42	165.95(15)	165.59(16)
N25-Co3-N27	97.15(17)	97.40(17)	N27-Ni1A-N41	89.13(15)	89.03(16)
N28-Co3-N25	178.37(18)	178.36(18)	N19-Ni1A-N42	88.75(15)	89.10(16)
N28-Co3-N26	98.54(17)	98.49(16)	N19-Ni1A-N41	166.28(15)	166.12(16)
N28-Co3-N27	81.66(17)	81.18(17)	N44-Ni1A-N27	90.86(15)	90.54(17)
N3-Co3-N10	91.54(18)	91.47(17)	N44-Ni1A-N19	89.91(16)	90.12(17)
N3-Co3-N25	87.70(17)	87.78(17)	N44-Ni1A-N42	78.57(15)	78.61(17)
N3-Co3-N28	93.56(17)	93.69(18)	N44-Ni1A-N41	99.68(16)	99.96(17)
N3-Co3-N26	90.03(18)	90.14(17)	N41-Ni1A-N42	83.63(15)	83.61(16)
N3-Co3-N27	173.98(17)	173.67(17)	N43-Ni1A-N27	91.30(15)	91.71(16)
N27-Co3-N26	87.08(17)	86.99(17)	N43-Ni1A-N19	90.36(17)	90.30(17)
[1]1-X, +Y, 1/2-Z			N43-Ni1A-N44	177.73(15)	177.60(17)
N17-Ni2-N49	90.07(16)	90.03(16)	N43-Ni1A-N42	99.19(15)	99.03(16)
N17-Ni2-N52	89.90(16)	89.91(16)	N43-Ni1A-N41	79.66(16)	79.24(16)
N17-Ni2-N51	170.08(16)	169.82(16)	N10-Ni2-N17	94.34(16)	94.35(16)
N50-Ni2-N49	175.31(16)	175.49(16)	N10-Ni2-N50	90.09(16)	90.61(17)
N50-Ni2-N52	99.64(16)	99.24(16)	N10-Ni2-N49	90.77(16)	90.34(16)
N50-Ni2-N51	78.54(15)	78.69(16)	N10-Ni2-N52	169.07(16)	168.95(16)
N49-Ni2-N52	79.13 (15)	79.45(16)	N10-Ni2-N51	92.71(16)	93.09(16)
N49-Ni2-N51	96.81(15)	96.86(15)	N17-Ni2-N50	94.46(16)	94.28(16)
N51-Ni2-N52	84.41(15)	84.04(15)	N34-Ni2A-N33	175.94(16)	176.29(17)
N26-Ni2A-N2	93.74(16)	93.35(17)	N34-Ni2A-N36	98.31(16)	98.57(16)
N26-Ni2A-N36	168.13(15)	168.56(17)	N34-Ni2A-N35	78.95(15)	78.96(16)
N26-Ni2A-N35	91.97(16)	92.56(16)	N33-Ni2A-N36	78.77(15)	78.94(16)
N2-Ni2A-N34	93.72(16)	93.68(16)	N33-Ni2A-N35	97.87(15)	97.98(16)
N2-Ni2A-N33	89.19(16)	89.14(16)	N26-Ni2A-N34	92.07(16)	91.55(17)
N2-Ni2A-N36	91.31(15)	91.34(16)	N26-Ni2A-N33	90.56(16)	90.69(17)
N2-Ni2A-N35	170.87(16)	170.69(16)	N25-Ni3A-N39	93.77(16)	94.03(17)
N35-Ni2A-N36	84.45(15)	84.19(16)	N25-Ni3A-N38	90.39(16)	90.34(17)
N18-Ni3-N46	90.53(17)	90.92(18)	N25-Ni3A-N40	88.80(16)	89.33(17)

N18-Ni3-N45	92.76(16)	92.89(16)	N25-Ni3A-N37	170.54(16)	169.87(17)
N18-Ni3-N47	90.84(18)	90.43(19)	N4-Ni3A-N25	93.49(16)	93.46(17)
N18-Ni3-N48	169.68(18)	169.30(18)	N4-Ni3A-N39	91.17(16)	90.61(18)
N11-Ni3-N18	94.45(16)	94.41(16)	N4-Ni3A-N38	170.94(17)	170.43(18)
N11-Ni3-N46	93.76(16)	93.97(16)	N4-Ni3A-N40	91.59(18)	91.38(19)
N11-Ni3-N45	169.71(17)	169.92(17)	N4-Ni3A-N37	93.17(16)	93.57(16)
N11-Ni3-N47	90.05(17)	89.73(17)	N39-Ni3A-N38	96.76(17)	97.88(18)
N11-Ni3-N48	90.43(16)	90.28(16)	N39-Ni3A-N40	176.11(17)	175.98(17)
N46-Ni3-N45	78.84(17)	78.96(17)	N39-Ni3A-N37	79.37(16)	78.62(17)
N46-Ni3-N48	98.24(17)	98.35(17)	N38-Ni3A-N37	84.02(16)	83.86(16)
N45-Ni3-N48	83.66(16)	83.72(16)	N40-Ni3A-N38	80.29(17)	79.88(18)
N47-Ni3-N46	175.84(17)	175.95(17)	N40-Ni3A-N37	97.73(16)	97.78(17)
N47-Ni3-N45	97.17(18)	97.16(17)			
N47-Ni3-N48	80.04(18)	79.97(18)			

Table S11. Detailed structure bond lengths and bond angles of Co^{II} ions for **4*R*** and **4*S*** at low and high temperature.

	4***R***			4***S***	
	**100K**	**250K**		**100K**	**250K**
Co1-N9	2.207(10)	2.244(9)	Co1-N2	2.131(8)	2.127(7)
Co1-N9^{3}	2.207(9)	2.244(9)	Co1-N2^{2}	2.131(8)	2.127(7)
Co1-N1^{3}	2.129(8)	2.121(8)	Co1-N9	2.092(9)	2.111(9)
Co1-N1	2.129(8)	2.121(8)	Co1-N9^{2}	2.092(9)	2.110(9)
Co1-N10	2.116(10)	2.090(9)	Co1-N10	2.213(8)	2.225(8)
Co1-N10^{3}	2.116(10)	2.090(9)	Co1-N10^{2}	2.213(8)	2.225(8)
Co2-N13	2.050(10)	2.072(9)	Co2-N1	1.979(7)	1.966(7)
Co2-N2	1.977(7)	1.981(7)	Co2-N8^{1}	1.975(10)	1.982(9)
Co2-N14	2.021(9)	2.045(8)	Co2-N11	2.047(7)	2.041(7)
Co2-N12	2.042(12)	2.073(11)	Co2-N12	2.085(8)	2.102(8)
Co2-N11	2.020(7)	2.047(11)	Co2-N13	2.059(10)	2.100(10)
Co2-N4^{1}	1.966(10)	1.986(11)	Co2-N14	2.040(9)	2.022(9)
Co3-N3^{1}	1.941(7)	1.948(7)	Co3-N3	1.938(7)	1.947(7)
Co3-N3^{2}	1.941(7)	1.948(7)	Co3-N3^{3}	1.938(8)	1.947(7)
Co3-N3	1.941(7)	1.948(7)	Co3-N3^{1}	1.938(8)	1.947(7)
Co3-N3^{3}	1.941(7)	1.948(7)	Co3-N3^{2}	1.938(7)	1.947(7)
N9-Co1-N9^{2}	99.4(5)	100.1(4)	N2^{2}-Co1-N2	94.8(4)	95.2(4)
N1-Co1-N9^{2}	83.9(3)	83.8(3)	N2^{2}-Co1-N10^{2}	170.5(3)	170.5(3)
N1^{2}-Co1-N9^{2}	169.7(4)	170.5(4)	N2-Co1-N10^{2}	83.4(3)	82.9(3)
N1-Co1-N9	169.7(4)	170.5(4)	N2^{2}-Co1-N10	83.4(3)	82.9(3)
N1^{2}-Co1-N9	83.9(3)	83.8(3)	N2-Co1-N10	170.5(3)	170.5(3)
N1^{2}-Co1-N1	94.5(4)	93.7(4)	N9^{2}-Co1-N2	97.3(3)	97.3(3)

N10-Co1-N9	76.7(4)	76.9(4)	N9-Co1-N2^{2}	97.3(3)	97.3(3)
N10^{2}-Co1-N9^{2}	76.7(4)	76.9(4)	N9-Co1-N2	93.7(3)	94.7(3)
N10^{2}-Co1-N9	92.6(4)	91.1(4)	N9^{2}-Co1-N2^{2}	93.7(3)	94.7(3)
N10-Co1-N9^{2}	92.6(4)	91.1(4)	N9^{2}-Co1-N9	163.6(4)	162.1(5)
N10^{2}-Co1-N1	97.7(3)	98.2(3)	N9-Co1-N10	77.3(3)	76.4(4)
N10-Co1-N1^{2}	97.7(3)	98.2(3)	N9^{2}-Co1-N10	92.1(3)	92.1(3)
N10^{2}-Co1-N1^{2}	93.5(3)	94.4(3)	N9-Co1-N10^{2}	92.1(3)	92.1(3)
N10-Co1-N1	93.5(3)	94.4(3)	N9^{2}-Co1-N10^{2}	77.3(3)	76.4(4)
N10^{2}-Co1-N10	163.6(4)	161.4(4)	N10-Co1-N10^{2}	99.9(4)	100.4(4)
N2-Co2-N13	82.6(4)	82.8(3)	N1-Co2-N11	90.7(3)	90.4(3)
N2-Co2-N14	91.9(3)	91.0(3)	N1-Co2-N12	82.6(3)	82.4(3)
N2-Co2-N12	170.9(4)	171.8(4)	N1-Co2-N13	173.5(4)	173.3(3)
N2-Co2-N11	92.1(4)	92.0(3)	N1-Co2-N14	91.8(4)	92.5(4)
N14-Co2-N13	79.5(4)	79.9(3)	N8^{1}-Co2-N1	91.9(3)	92.5(3)
N14-Co2-N12	97.2(4)	97.1(4)	N8^{1}-Co2-N11	92.1(3)	92.2(3)
N12-Co2-N13	98.6(4)	97.6(4)	N8^{1}-Co2-N12	169.1(3)	169.7(3)
N11-Co2-N13	95.7(4)	95.3(4)	N8^{1}-Co2-N13	89.5(4)	89.3(3)
N11-Co2-N14	173.4(4)	174.0(4)	N8^{1}-Co2-N14	94.8(4)	94.0(4)
N11-Co2-N12	78.8(4)	79.8(4)	N11-Co2-N12	78.7(3)	78.9(3)
N4^{3}-Co2-N13	168.1(4)	168.9(3)	N11-Co2-N13	95.6(3)	96.0(3)
N4^{3}-Co2-N2	91.5(4)	92.0(3)	N13-Co2-N12	97.0(3)	96.7(3)
N4^{3}-Co2-N14	90.4(4)	90.5(4)	N14-Co2-N11	172.6(4)	173.0(4)
N4^{3}-Co2-N12	88.9(4)	89.0(4)	N14-Co2-N12	94.8(4)	95.1(4)
N4^{3}-Co2-N11	94.8(4)	94.6(4)	N14-Co2-N13	81.7(4)	81.0(4)
N3-Co3-N3^{1}	116.6(5)	117.5(5)	N3-Co3-N3^{3}	115.7(5)	117.3(5)
N3^{2}-Co3-N3^{1}	104.8(4)	103.6(5)	N3^{1}-Co3-N3^{3}	108.2(4)	107.9(4)
N3-Co3-N3^{2}	107.3(4)	107.6(4)	N3-Co3-N3^{1}	104.7(4)	103.6(4)
N3^{3}-Co3-N3^{2}	116.6(5)	117.5(5)	N3-Co3-N3^{2}	108.2(4)	107.9(4)
N3^{3}-Co3-N3^{1}	107.3(4)	107.6(4)	N3^{1}-Co3-N3^{2}	115.7(5)	117.3(5)
N3-Co3-N3^{3}	104.8(4)	103.6(5)	N3^{2}-Co3-N3^{3}	104.7(4)	103.6(4)
^{1}1-X, +Y, 1-Z; ^{2}+X, 1-Y, 1-Z; ^{3}1-X, 1-Y, +Z			^{1}1-X, +Y, 1-Z; ^{2}1-X, 1-Y, +Z; ^{3}+X, 1-Y, 1-Z		

Table S12. Comparison of Fe-N_{CN}, Fe-N_{py} and Fe-N_{ami} bond lengths, average M-N bond lengths and octahedral distortion bond angle parameters ∑ in HT and LT phase of complexes **1**^{de} together with the Fe position in the cluster and spin state assignment.

	**1**^{de}	
	**300K**	**100K**
Fe1-N_{CN}	2.055	1.933
Fe1-N_{Py}	2.140	1.991
Fe1-N_{ami}	2.236	2.077
**Fe1-N_{av}**	**2.144**	**2.001**
**∑(deg)**	**70.48**	**50.11**
vertex	**HS**	**LS**
Fe2-N_{CN}	1.906	1.909
Fe2-N_{Py}	1.979	1.980
Fe2-N_{ami}	2.053	2.058
**Fe2-N_{av}**	**1.979**	**1.983**
**∑(deg)**	**52.44**	**50.24**
lateral	**LS**	**LS**
Fe3-N_{CN}	1.919	1.921
Fe3-N_{Py}	1.979	1.972
Fe3-N_{ami}	2.070	2.059
**Fe3-N_{av}**	**1.989**	**1.984**
**∑(deg)**	**48.09**	**47.18**
lateral	**LS**	**LS**

Table S13. Comparison of Ni-N_{CN}, Ni-N_{py} and Ni-N_{ami} bond lengths, average M-N bond lengths and octahedral distortion bond angle parameters ∑ in HT and LT phase of complex **3**.

	**3**	
	**250K**	**100K**
Ni1-N_{CN}	2.094	2.078
Ni1-N_{Py}	2.099	2.093
Ni1-N_{ami}	2.172	2.171
**Ni1-N_{av}**	**2.121**	**2.114**
**∑(deg)**	**64.32**	**67.2**
Ni1A-N_{CN}	2.109	2.110
Ni1A-N_{Py}	2.097	2.090
Ni1A-N_{ami}	2.177	2.174
**Ni1A-N_{av}**	**2.127**	**2.125**
**∑(deg)**	**62.56**	**62.33**
Ni2-N_{CN}	2.048	2.049
Ni2-N_{Py}	2.091	2.087
Ni2-N_{ami}	2.151	2.150
**Ni2-N_{av}**	**2.093**	**2.095**
**∑(deg)**	**56.71**	**56.91**
Ni2A-N_{CN}	2.054	2.052
Ni2A-N_{Py}	2.088	2.085
Ni2A-N_{ami}	2.160	2.156
**Ni2A-N_{av}**	**2.101**	**2.097**
**∑(deg)**	**58.49**	**58.19**
Ni3-N_{CN}	2.050	2.052
Ni3-N_{Py}	2.084	2.084
Ni3-N_{ami}	2.160	2.156
**Ni3-N_{av}**	**2.098**	**2.098**
**∑(deg)**	**56.03**	**55.69**
Ni3A-N_{CN}	2.049	2.047
Ni3A-N_{Py}	2.077	2.079
Ni3A-N_{ami}	2.149	2.152
**Ni3A-N_{av}**	**2.092**	**2.092**
**∑(deg)**	**57.36**	**55.59**

Table S14. Transition completeness of **1** and **1de** obtained from SC-XRD, magnetic and Mössbauer spectra measurements.

	**1**			**1de**		
	SC-XRD (Fe1-N, Å)	Magnetic data (cm^3 K mol^{-1})	Mössbauer spectra (HS fraction)	SC-XRD (Fe1-N, Å)	Magnetic data (cm^3 K mol^{-1})	Mössbauer spectra (HS fraction)
HT phase	2.169	12.07	0.37	2.144	11.46	0.44
LT phase	2.025	7.34	0.20	2.001	6.03	0.18
^{a}Transition completeness	76%	60-75%	65%	87%	64-82%	71%

^{a}The calculation results from the assumption that the bond length of the fully low-spin Fe^{II} is 1.98 Å and that the Fe1 is high-spin state in both **1** and **1de** at HT phase.

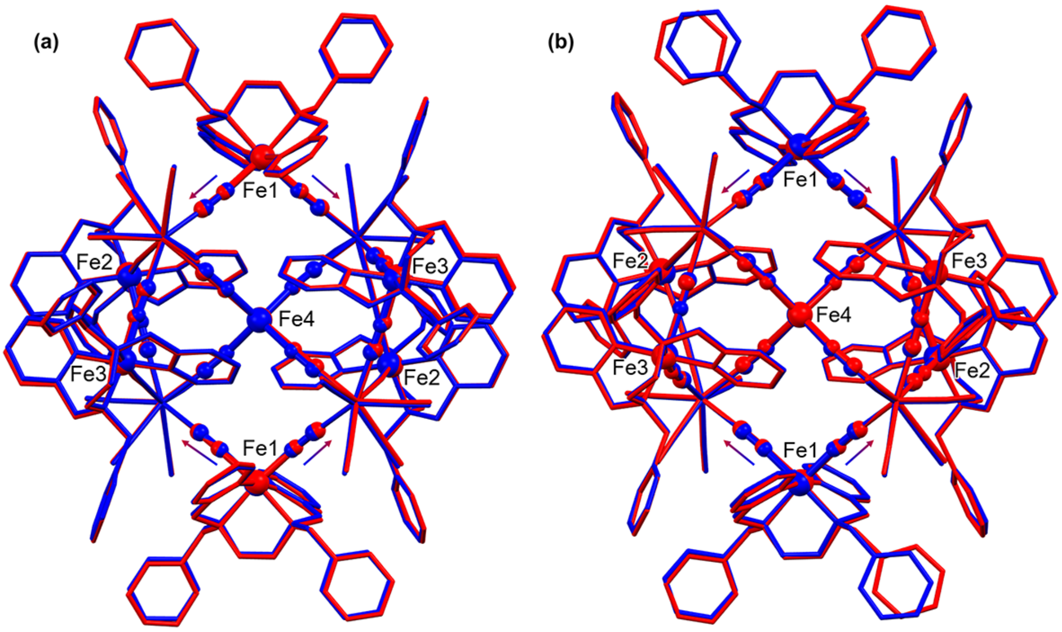

Figure S33. Overlays of the high spin (red) and low spin (blue) structures of **1** (a) and $\mathbf{1^{de}}$ (b). The arrows represent elongated direction of CN-Fe linkages with the temperature, which occurs at the SCO active Fe1 sites.

During SCO transition a moderate compression is noted both in **1** and $\mathbf{1^{de}}$ with temperature decreasing from 250 K (or 300 K) to 100 K. For **1** the unit cell volume compresses in rather an isotropic manner from 22778.8(11) Å^3 to 22146.3(10) Å^3 (*ca.* 2.8 %), whereas for $\mathbf{1^{de}}$ the unit cell compression 20769(3) Å^3 to 20226.1(8) Å^3 (*ca.* 2.6 %) $\mathbf{1^{de}}$ is dominated by the shortening of *c* period of 2%, contrasting with the shortening of 0.2% and 0.6% observed for the periods *b* and *a* respectively (See Table S1). This might be correlated with the increased overall degree of freedom along the *c* direction as a consequence of the removal of MeOH molecules, all located in the channels running along this direction, and one of them present in the space between the vertex regions of clusters (*compare* Figure 2 and Figure S25). The representative overlays of the high spin and low spin structures of **1** and $\mathbf{1^{de}}$ also exhibit the elongated or compressed CN-Fe linkages at the Fe1 sites along this direction (Figure S33).

6. Bond valence sum (BVS) calculations

Table S15. Bond valence sum (BVS) calculations for **1** and **1de** at LT phase and HT phase. These were calculated using parameters for high spin or low spin Fe-N bonds. The bold font is the suggested valence state.

**1**					
Metal center	Spin state	Bond Valence Sum		Expected bond length (Å)	Actual bond length (Å)
		100K	250K		
Fe1	HS	2.967	**2.030**	2.166	2.025(100K)
	LS	**1.728**	1.182	1.966	2.169(250K)
Fe2	HS	3.371	3.337	2.166	1.978(100K)
	LS	**1.963**	**1.943**	1.966	1.982(250K)
Fe3	HS	3.317	3.316	2.166	1.983(100K)
	LS	**1.943**	**1.932**	1.966	1.984(250K)
Fe4	HS	**1.968**	**1.888**	2.016	2.023(100K) 2.037(250K)
**1de**					
Metal center	Spin state	Bond valence sum		Expected bond length (Å)	Actual bond length (Å)
		100K	300K		
Fe1	HS	3.175	**2.166**	2.166	2.001(100K)
	LS	**1.849**	1.262	1.966	2.144(300K)
Fe2	HS	3.330	3.360	2.166	1.983(100K)
	LS	**1.939**	**1.957**	1.966	1.979(300K)
Fe3	HS	3.317	3.274	2.166	1.984(100K)
	LS	**1.932**	**1.907**	1.966	1.989(300K)
Fe4	HS	**1.975**	**1.966**	2.016	2.021(100K) 2.023(300K)

The large deviation shown by Fe1 at 100 K for Fe1 is due to the fact Fe1 is in a mixed spin state population at this temperature.

Table S16. Bond valence sum (BVS) calculations for **2** and **3** at LT phase and HT phase. These were calculated using parameters for high spin or low spin Fe-N bonds. The bold font is the suggested valence state.

**2**					
Metal center	Spin state	Bond Valence Sum		Expected bond length (Å)	Actual bond length (Å)
		100K	250K		
Co1	HS	**1.837**	**1.782**	2.126	2.164(100K) 2.175(250K)
	LS	1.328	1.289	2.006	
Co2	HS	2.706	2.628	2.126	2.017(100K) 2.027(250K)
	LS	**1.957(+2)**	**1.900(+2)**	2.006	
	LS	2.496(+3)	2.424(+2)	1.946	
Co3	HS	3.052	3.018	2.126	1.974(100K) 1.977(250K)
	LS	**2.207(+2)**	**2.182(+2)**	2.006	
	LS	2.814(+3)	2.782(+3)	1.946	
**3**					
Metal center	Spin state	Bond Valence Sum		Expected bond length (Å)	Actual bond length (Å)
		100K	250K		
Ni1	HS	**1.973**	**1.932**	2.106	2.114(100K) 2.122(250K)
Ni2	HS	**2.093**	**2.094**	2.106	2.093(100K) 2.093(250K)
Ni3	HS	**2.064**	**2.045**	2.106	2.098(100K) 2.101(100K)
Ni1A	HS	**1.914**	**1.903**	2.106	2.125(100K) 2.127(250K)
Ni2A	HS	**2.084**	**2.066**	2.106	1.983(100K) 1.979(250K)
Ni3A	HS	**2.075**	**2.065**	2.106	2.096(100K) 2.098(250K)

Table S17. Bond valence sum (BVS) calculations for **4*S*** and **4*R*** at LT phase and HT phase. These were calculated using parameters for high spin or low spin M-N bonds. The bold font is the suggested valence state.

<table>
<tr><th colspan="6">4S</th></tr>
<tr><th rowspan="2">Metal center</th><th rowspan="2">Spin state</th><th colspan="2">Bond valence sum</th><th rowspan="2">Expected bond length (Å)</th><th rowspan="2">Actual bond length (Å)</th></tr>
<tr><th>100K</th><th>250K</th></tr>
<tr><td rowspan="2">Co1</td><td>HS</td><td>1.919</td><td>1.875</td><td>2.126</td><td rowspan="2">2.145(100K)
2.154(250K)</td></tr>
<tr><td>LS</td><td>1.387</td><td>1.355</td><td>2.006</td></tr>
<tr><td rowspan="3">Co2</td><td>HS</td><td>2.606</td><td>2.580</td><td>2.126</td><td rowspan="3">2.031(100K)
2.036(250K)</td></tr>
<tr><td>LS</td><td>1.884 (+2)</td><td>1.865 (+2)</td><td>2.006</td></tr>
<tr><td>LS</td><td>2.403 (+3)</td><td>2.379 (+3)</td><td>1.946</td></tr>
<tr><td>Co3</td><td>HS</td><td>2.223</td><td>2.160</td><td>1.976</td><td>1.938(100K)
1.947(250K)</td></tr>
<tr><th colspan="6">4R</th></tr>
<tr><th rowspan="2">Metal center</th><th rowspan="2">Spin state</th><th colspan="2">Bond valence sum</th><th rowspan="2">Expected bond length (Å)</th><th rowspan="2">Actual bond length (Å)</th></tr>
<tr><th>100K</th><th>250K</th></tr>
<tr><td rowspan="2">Co1</td><td>HS</td><td>1.855</td><td>1.873</td><td>2.126</td><td rowspan="2">2.151(100K)
2.152(250K)</td></tr>
<tr><td>LS</td><td>1.341</td><td>1.354</td><td>2.006</td></tr>
<tr><td rowspan="3">Co2</td><td>HS</td><td>2.694</td><td>2.635</td><td>2.126</td><td rowspan="3">2.013(100K)
2.034(250K)</td></tr>
<tr><td>LS</td><td>1.948 (+2)</td><td>1.905 (+2)</td><td>2.006</td></tr>
<tr><td>LS</td><td>2.485 (+3)</td><td>2.430 (+3)</td><td>1.946</td></tr>
<tr><td>Co3</td><td>HS</td><td>2.220</td><td>2.134</td><td>1.976</td><td>1.941(100K)
1.948(250K)</td></tr>
</table>

7. Magnetic characteristics

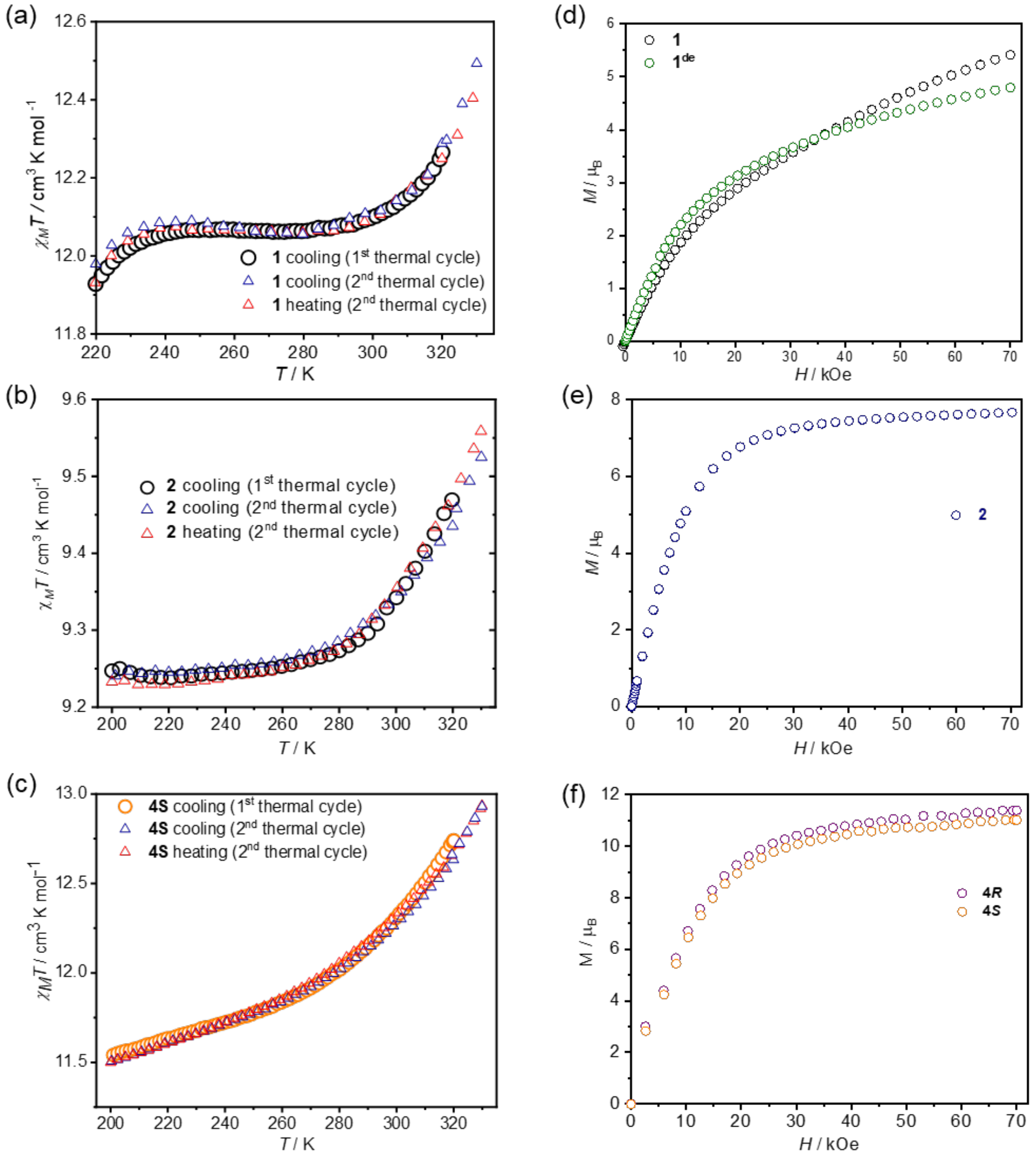

Figure S34. Expanded $\chi_M T(T)$ plot of **1** (a), **2** (b) and **4*S*/4*R*** (c) shows unequivocally the onset SCO behavior above room temperature; *M* versus *H* plot at 2.0 K for **1** and **1**$^{\mathbf{de}}$ (d), **2** (e) and **4*S*** and **4*R*** (f).

Table S18. Ni-N≡C angles within the cyanido-bridged skeleton of **3**.

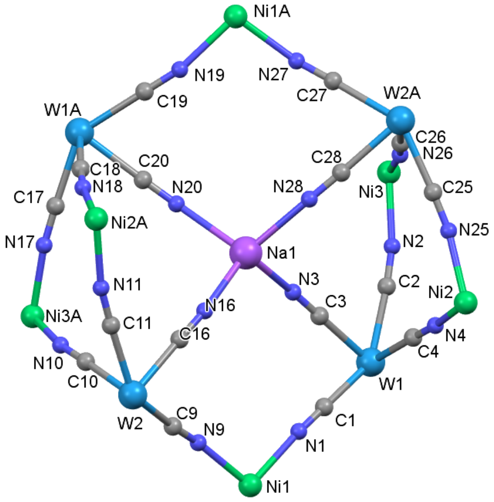

Atoms	C-N-Ni	Angle
Ni1A	C27-N27-Ni1A	161.7
	C19-N19-Ni1A	163.5
Ni2A	C18-N18-Ni2A	172.6
	C11-N11-Ni2A	169.9
Ni3A	C17-N17-Ni3A	171.3
	C10-N10-Ni3A	164.9
Ni1	C1-N1-Ni1	164.5
	C9-N9-Ni1	161.4
Ni2	C4-N4-Ni2	172.1
	C25-N25-Ni2	167.1
Ni3	C2-N2-Ni3	169.4
	C26-N26-Ni3	171.4

In general, the F interactions are due the orthogonality of SOMOs, which is ensured by the σ character of the e_g-type natural magnetic orbitals of Ni moiety and π character of the natural magnetic orbitals of $[W(CN)_8]^{3-}$, d_z^2 in square antiprism geometry or d_{x2-y2} (d_{xy}) in trigonal dodecahedral geometry. The maximum J of up to 15-20 cm^{-1} (2J formalism) might be claimed for the close-to-linear Ni-N≡C angle, and the systematic decrease of J is noted with the decrease of this angle. When the angles are smaller than *ca.* 145-150 deg, antiferromagnetic Ni^{II}-W^{V} interactions and the negative J can be observed.[11] In the case of **3** those angles are between 161 and 173 deg. (*av.* 167.4 deg, see Table and Figure below), which suggest the average J of *ca.* 10 cm^{-1}.[11-14]

8. Spectroscopic studies.

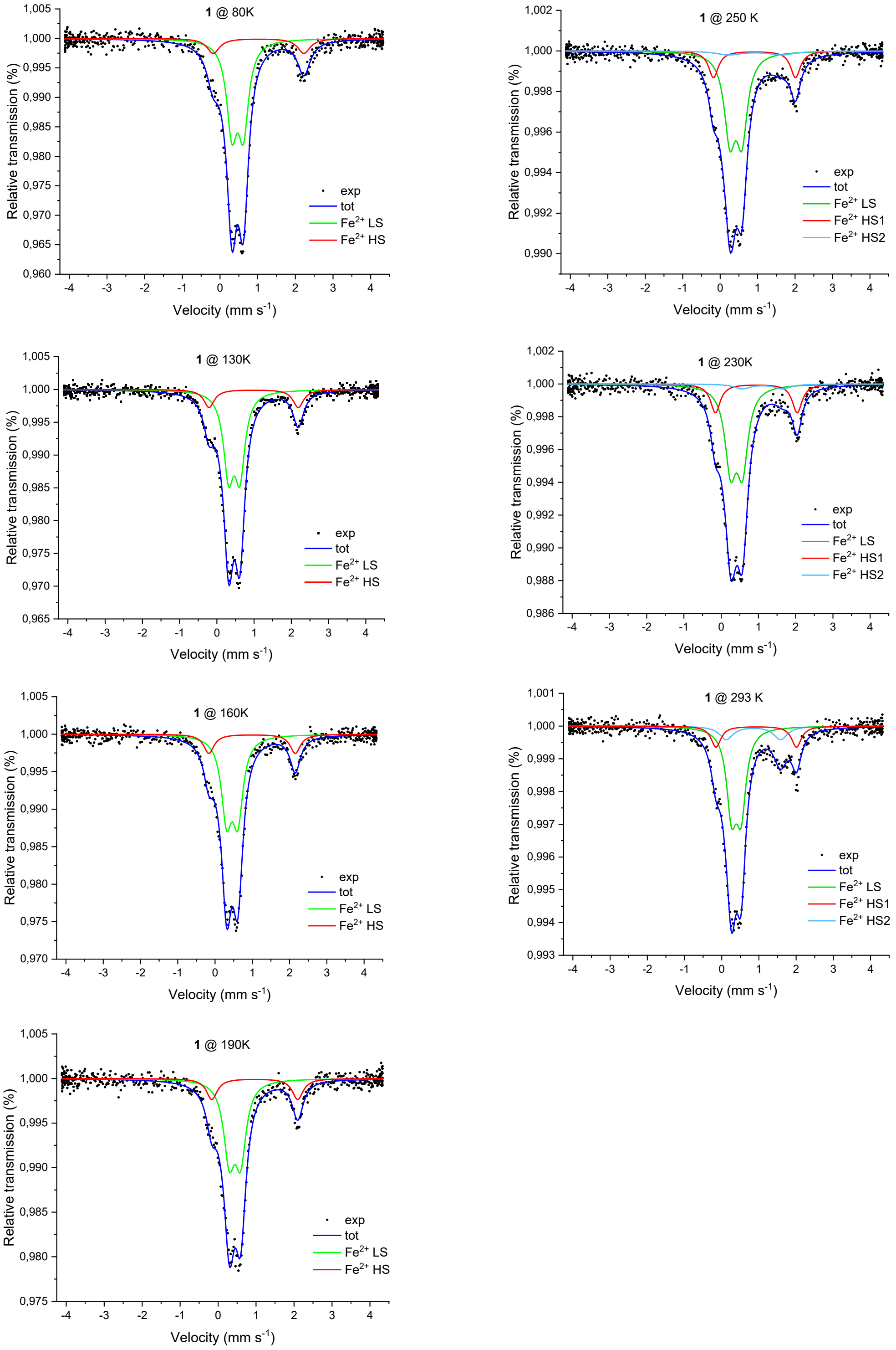

Figure S35. Temperature dependent ^{57}Fe Mössbauer spectra for **1** together with the fits.

Table S19. The most important parameters of ^{57}Fe Mössbauer spectra for **1**.

T (K)	Fe site	δ_{iso} / mm s^{-1}	Δ_{QS} / mm s^{-1}	Γ / mm s^{-1}	LS %	HS %
80(1)	Fe^{2+} LS	0.47(1)	0.31(1)	0.34(1)	80(2)	20(2)
	Fe^{2+} HS1	1.02(1)	2.41(2)	0.41(3)	20(2)	
	Fe^{2+} HS2					
130(1)	Fe^{2+} LS	0.47(1)	0.30(1)	0.33(1)	80(2)	20(2)
	Fe^{2+} HS1	0.99(1)	2.40(1)	0.35(1)	20(2)	
	Fe^{2+} HS2					
160(1)	Fe^{2+} LS	0.46(1)	0.30(1)	0.34(1)	80(2)	20(2)
	Fe^{2+} HS1	0.99(1)	2.32(2)	0.34(2)	20(2)	
	Fe^{2+} HS2					
190(1)	Fe^{2+} LS	0.44(1)	0.30(1)	0.35(1)	78(2)	22(2)
	Fe^{2+} HS1	0.97(1)	2.26(2)	0.35(2)		
	Fe^{2+} HS2					
230(1)	Fe^{2+} LS	0.41(1)	0.32(1)	0.36(1)	71(2)	29(2)
	Fe^{2+} HS1	0.94(1)	2.19(1)	0.32(3)	20(2)	
	Fe^{2+} HS2	1.11(10)	1.08(24)	0.53(12)	9(2)	
250(1)	Fe^{2+} LS	0.42(F)	0.32(F)	0.36(F)	66(2)	34(2)
	Fe^{2+} HS1	0.94(2)	2.19(F)	0.32(F)	17(2)	
	Fe^{2+} HS2	1.21(1)	1.42(9)	0.84(9)	17(2)	
293(2)	Fe^{2+} LS	0.39(1)	0.25(1)	0.32(1)	63(2)	37(2)
	Fe^{2+} HS1	0.93(1)	2.16(2)	0.32(F)	16(2)	
	Fe^{2+} HS2	0.85(3)	1.46(3)	0.45(5)	21(2)	

(F) – the parameter was fixed.

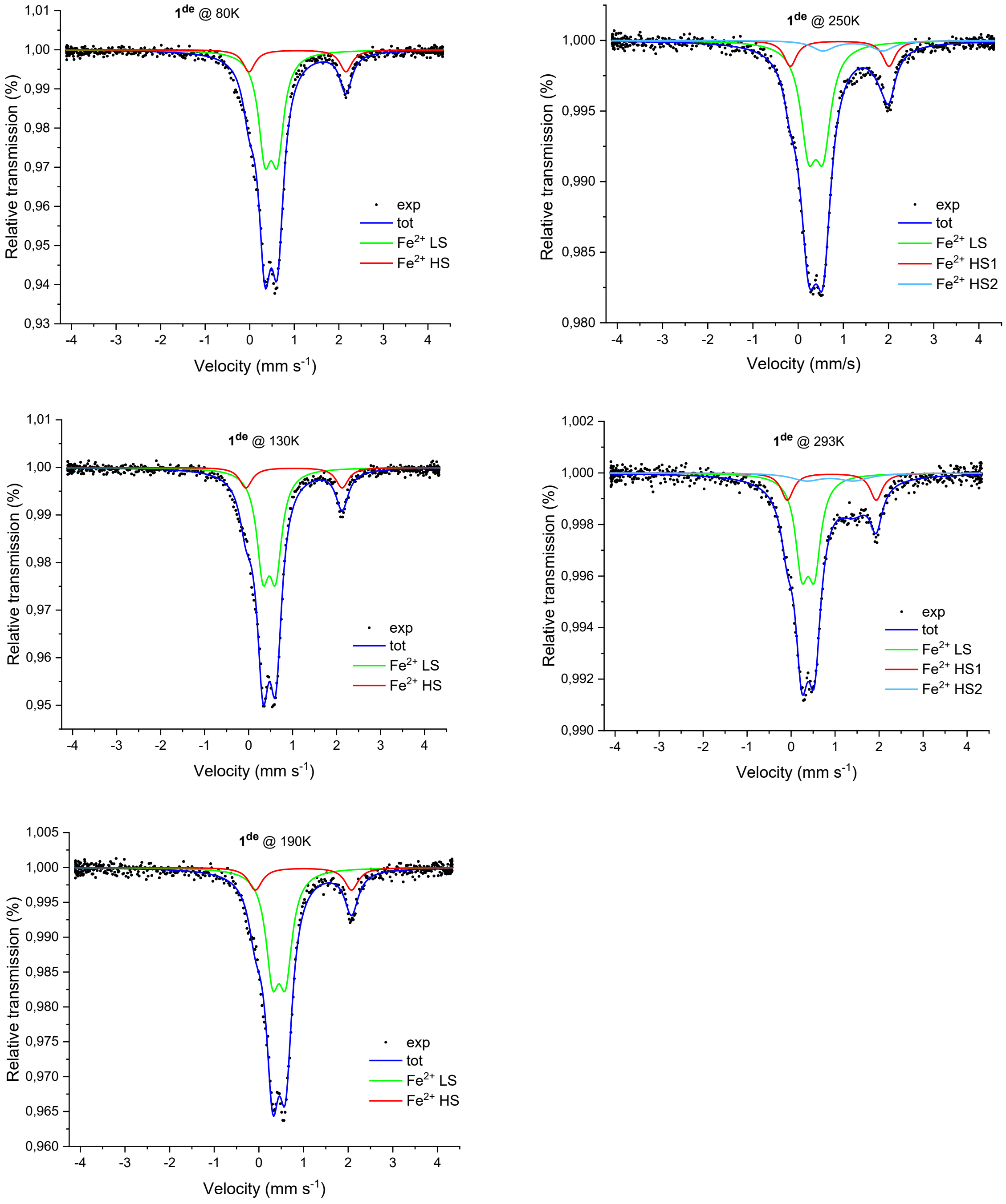

Figure S36. Temperature dependent ^{57}Fe Mössbauer spectra for **1de** together with the fits.

Table S20. The most important parameters of ^{57}Fe Mössbauer spectra for $\mathbf{1^{de}}$.

T (K)	Fe site	δ_{iso} / mm s^{-1}	Δ_{QS} / mm s^{-1}	Γ / mm s^{-1}	LS %	HS %
80(1)	Fe^{2+} LS	0.48(1)	0.29(1)	0.35(1)	82(2)	18(2)
	Fe^{2+} HS1	1.07(1)	2.17(1)	0.33(1)	18(2)	
	Fe^{2+} HS2					
130(1)	Fe^{2+} LS	0.48(1)	0.29(1)	0.35(1)	82(2)	18(2)
	Fe^{2+} HS1	1.07(1)	2.17(1)	0.33(1)	18(2)	
	Fe^{2+} HS2					
190(1)	Fe^{2+} LS	0.45(1)	0.29(1)	0.36(1)	80(2)	20(2)
	Fe^{2+} HS1	1.00(1)	2.15(1)	0.37(2)	20(2)	
	Fe^{2+} HS2					
250(1)	Fe^{2+} LS	0.39(1)	0.33(1)	0.42(1)	72(2)	28(2)
	Fe^{2+} HS1	0.92(1)	2.18(2)	0.34(F)	13(2)	
	Fe^{2+} HS2	1.21(1)	1.32(1)	0.59(1)	15(2)	
293(2)	Fe^{2+} LS	0.39(1)	0.29(1)	0.35(1)	56(2)	44(2)
	Fe^{2+} HS1	0.92(1)	2.02(2)	0.34(4)	16(2)	
	Fe^{2+} HS2	0.89(3)	1.07(9)	0.86(10)	28(2)	

(F) – the parameter was fixed.

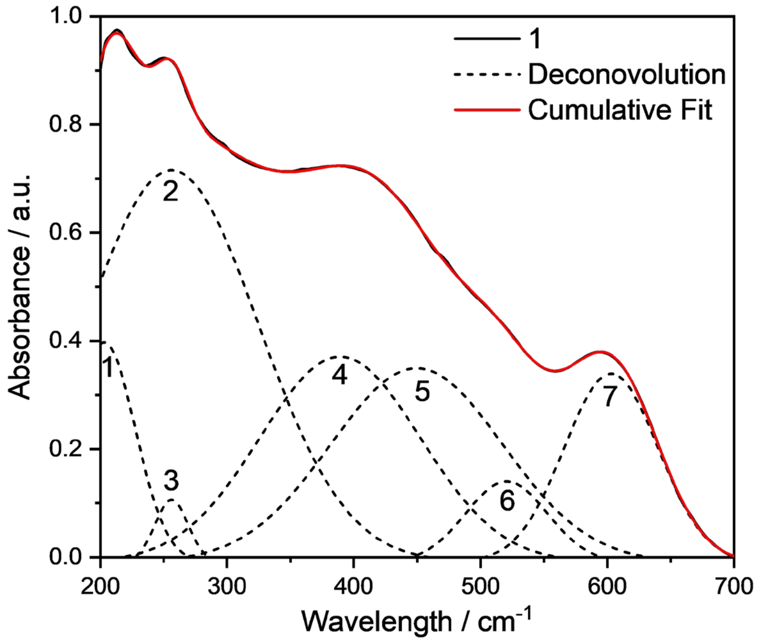

Figure S37. Solid-state UV-Vis-NIR absorption spectra of **1** in the 200 – 700 nm range together with the deconvoluted absorption components 1-7. The black solid lines represent the experimental data, and the red solid lines show the calculated sums while the black dotted lines represent the absorption components. *Assignment*: Peaks 1 (203 nm) – π-π* transitions of bzbpen ligands; peaks 2 (256 nm), 3 (257 nm) and 4 (389 nm) – CT transitions within $[W(CN)_8]^{3-/4-}$; peak 5 (449 nm) – ligand field transition (LF) of $[W(CN)_8]^{4-}$; peaks 6 (520 nm) and peak 7 (603 nm) – LF transitions of $[Fe^{II}(bzbpen)(\mu\text{-}NC)_2]$. The above assignment is also representative to the spectra of $\mathbf{1^{de}}$.

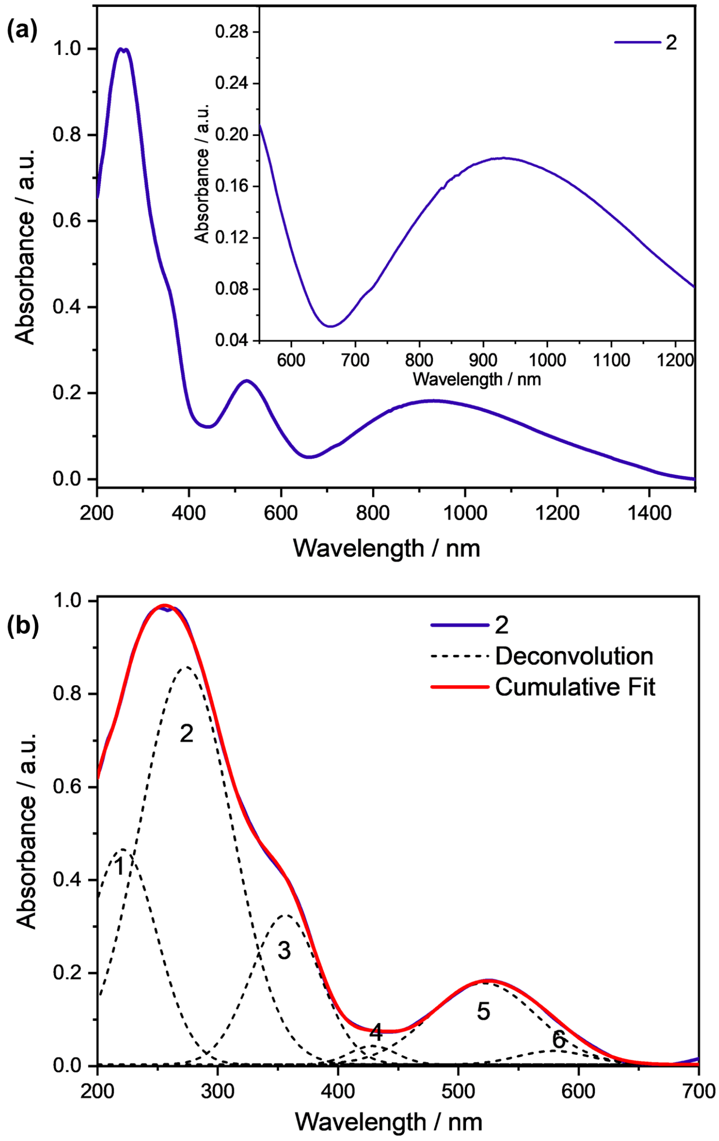

Figure S38. (a) Solid-state UV-Vis-NIR absorption spectra of **2** in the range of 200 – 1500 nm. Inset: expanded plot of the Vis-NIR part above 550 nm; (b) Deconvoluted absorption components 1-6 in the range of 200-700 nm; The blue solid lines represent the experimental data, and the red solid lines show the calculated sums while the black dotted lines represent the absorption components. *Assignment*: peaks 1 (221 nm) and 2 (272 nm) – π-π* transitions of bzbpen ligands and CT transitions within $[W(CN)_8]^{3-/4-}$; peaks 3 (355 nm) – CT transitions of $[W(CN)_8]^{3-/4-}$; peak 4 (430 nm) – LF transitions of $[W(CN)_8]^{4-}$; peak 5 (520 nm): metal-to-metal charge transfer (MMCT) from Co^{II}_{HS} to W^{V}; peak 6 (580 nm): d-d transition in high spin Co^{II}. The other LF transition of Co^{II} causes broad peak at 920 nm (*see* Figure 6).

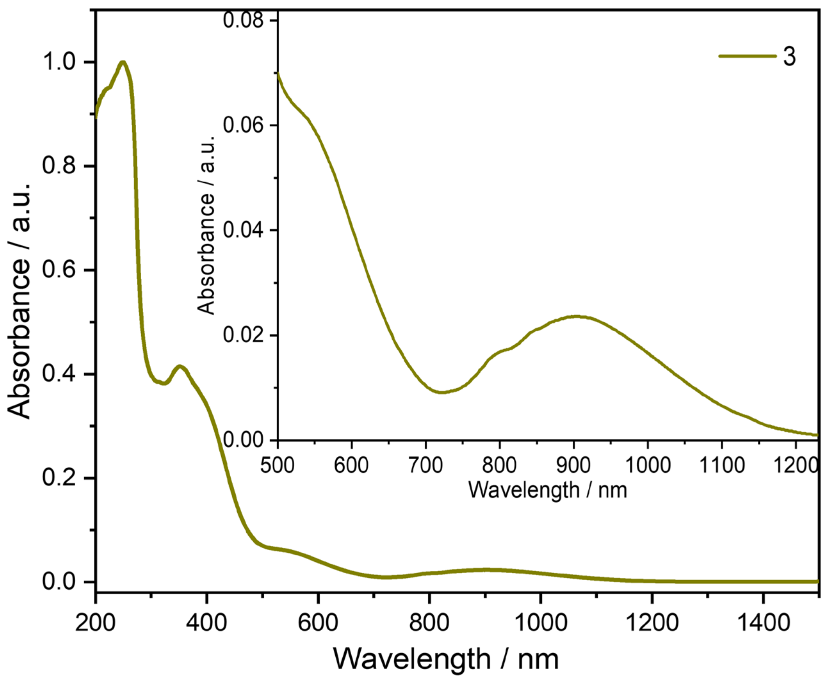

Figure S39. Solid-state UV-Vis-NIR absorption spectra of **3** in the 200 – 1500 nm range. Inset: expanded plot of the Vis-NIR part above 500 nm. *Assignment:* λ = 200-300 nm – transitions of bzbpen ligands and CT transitions of $[W(CN)_8]^{3-/4-}$; λ = 300-450 nm – CT and LF transitions of $[W(CN)_8]^{3-/4-}$; λ > 450 nm LF transitions of $[Ni^{II}(bzbpen)(\mu\text{-}NC)_2]$ moieties.

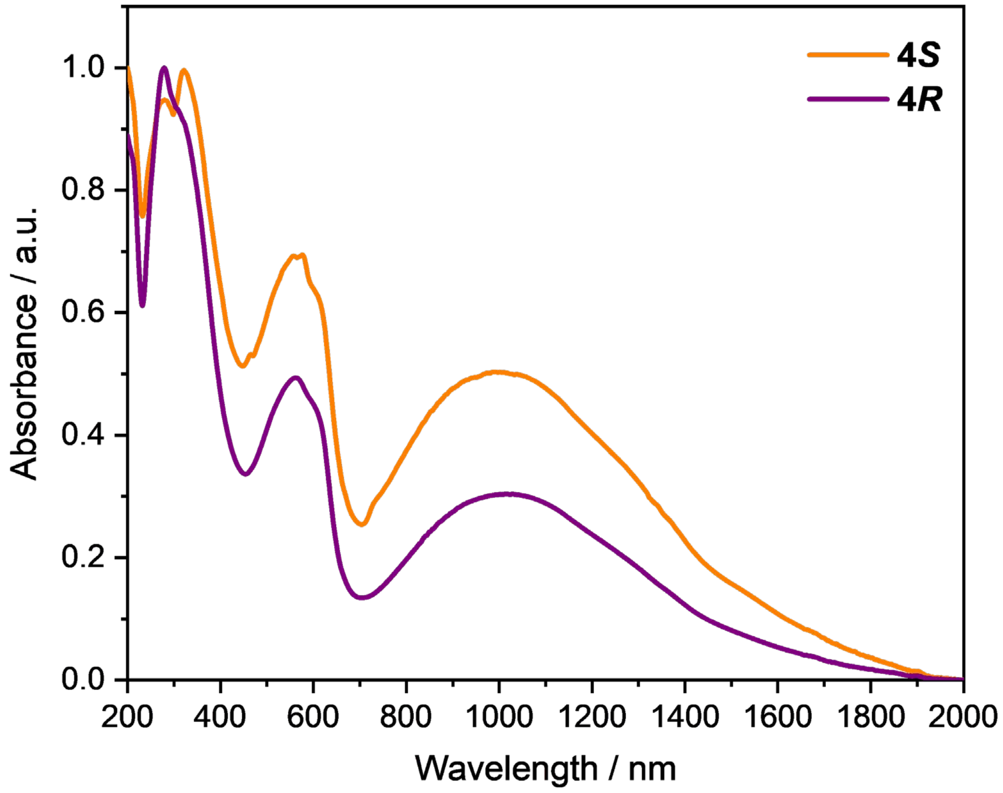

Figure S40. Solid-state UV-Vis-NIR absorption spectra of **4*R*** and **4*S*** in the 200 – 2000 nm range. *Assignment*: λ = 200-300 nm - transitions of *S*-pabh ligands; λ = 240-440 nm – transitions of $[W(CN)_8]^{3-/4-}$; λ = 450-650 nm – MMCT transition from Co^{II}_{HS} to W^{V}, and d-d transitions in high spin Co^{II}. The other d-d transition of Co^{II} causes broad peak at 1000 nm.

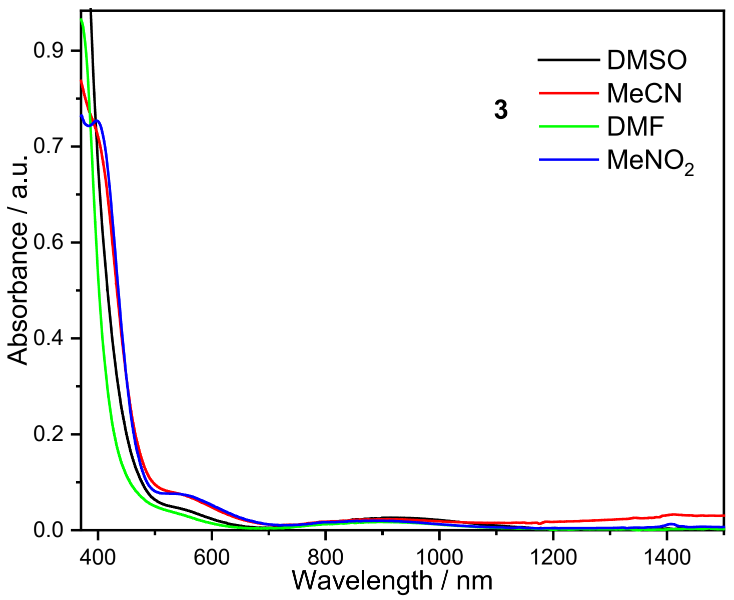

Figure S41. UV-vis-NIR spectra of **3** (0.1 mmol/L) in different solvents at room temperature.

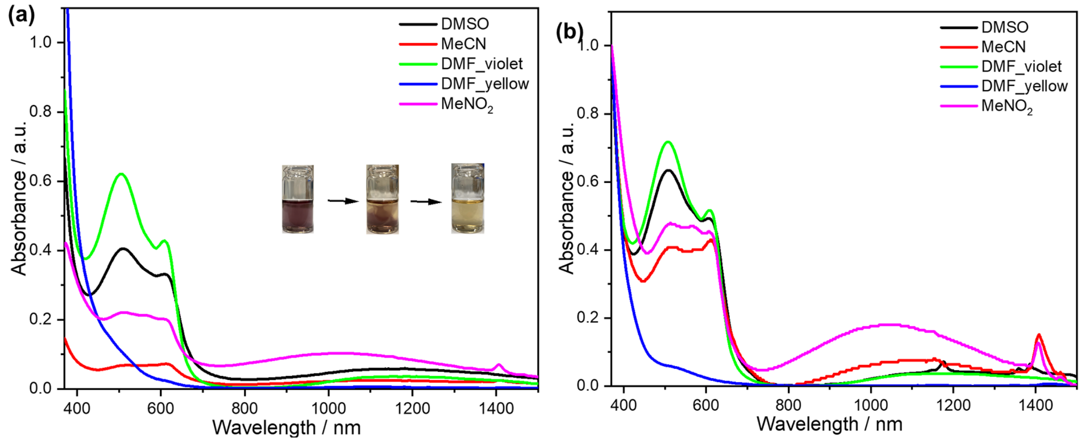

Figure S42. UV-vis-NIR spectra of 4R (a) and 4S (b) (0.2 mmol/L) in different solvents at room temperature. Photographs in (a) shows the decomposition of **4*R*/4*S*** in DMF solvent.

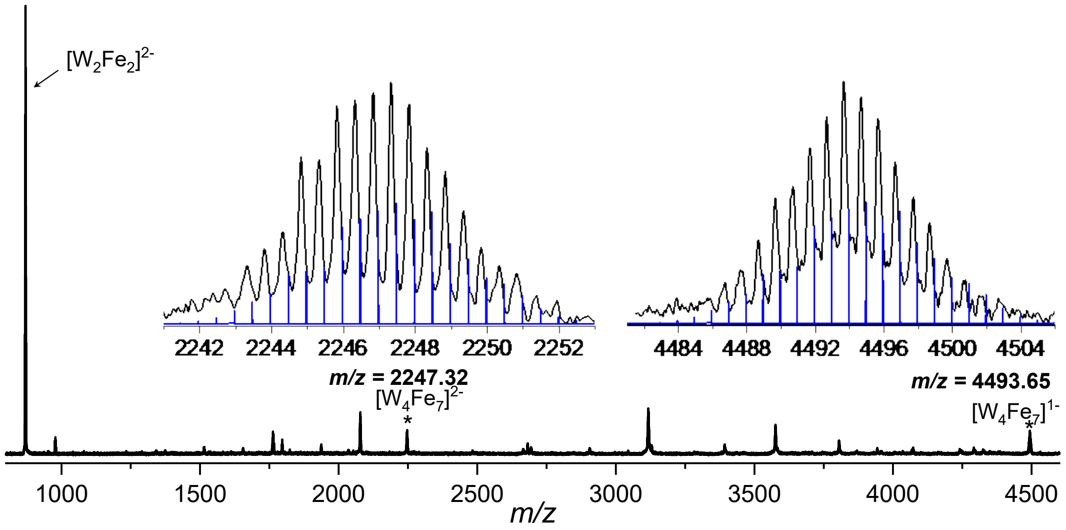

Figure S43. Electrospray mass spectrum of **1**, showing the peak-sets for $[W_4Fe_7]^{1-}$ and $[W_4Fe_7]^{2-}$ anionic motifs. The inset shows an expansion (black line) and simulation (blue line) of these two peaks.

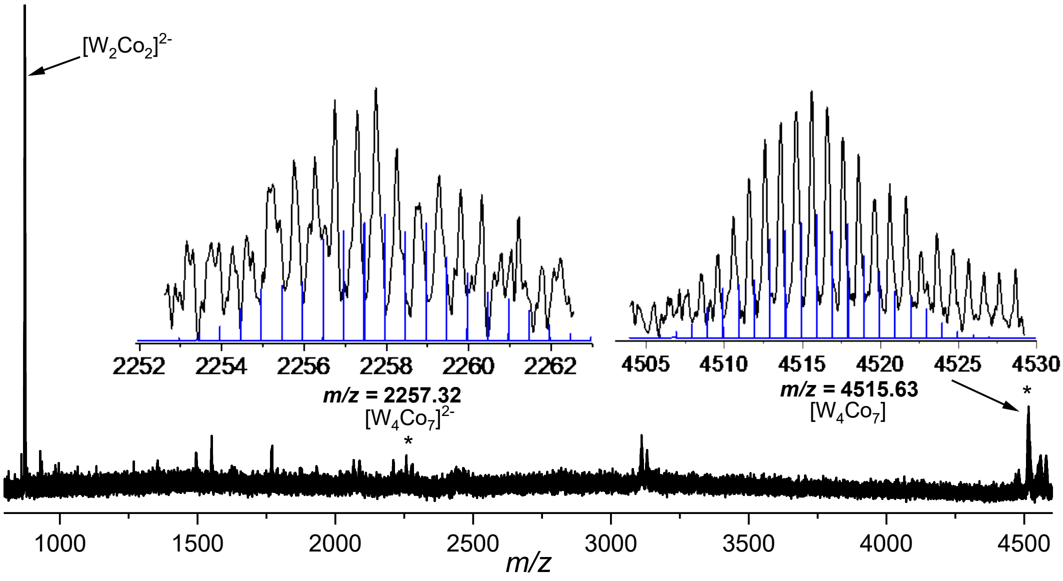

Figure S44. Electrospray mass spectrum of **2**, showing the peak-sets for $[W_4Co_7]^{1-}$ and $[W_4Co_7]^{2-}$ anionic motifs. The inset shows an expansion (black line) and simulation (red line) of these two peaks.

9. References

1. Bok, L. D. C.; Leipoldt, J. G.; Basson, S. S. The preparation of $Cs_3Mo(CN)_8\cdot 2H_2O$ and $Cs_3W(CN)_8\cdot 2H_2O$. *Z. Anorg. Allg. Chem.* **1975**, 415, 81-83.
2. Pribush, R.; Archer, R. D. Transition Metal Eight-Coordination. VI. Isomerization through Electronic and Environmental Effects-Electron Spin Resonance, Magnetic Circular Dichroism, and Electronic Spectra of Octacyanotungstate (IV) and (V). *Inorg. Chem.* **1974**, 13, 2556.
3. Zheng, C.; Jia, S.; Dong, Y.; Xu, J.; Sui, H.; Wang, F.; Li, D. Symmetry Breaking and Two-Step Spin-Crossover Behavior in Two Cyano-Bridged Mixed-Valence $\{Fe^{III}_2(\mu\text{-}CN)_4Fe^{II}_2\}$ Cluster. *Inorg. Chem.* **2019**, 58, 14316-14324.
4. Shiga, T.; Iijima, F.; Tetsuka, T.; Newton G. N.; Oshio, H. $[M_6M'_4]$ Cage Compounds with Chiral Bidentate Ligands. *Macromol. Symp.* **2012**, *317-318*, 286–292.
5. Bain, G. A.; Berry, J. F. Diamagnetic Corrections and Pascal's Constants. *J. Chem. Educ*. **2008**, 85, 532.
6. Sheldrick, G. A Short History of SHELX. *Acta Crystallogr. Sect. A: Found. Crystallogr*. **2008**, A64, 112-122.
7. Sheldrick, G. Crystal Structure Refinement with SHELXL. *Acta Crystallogr., Sect. C: Struct. Chem*. **2015**, C71, 3-8.
8. Dolomanov, O. V.; Bourhis, L. J.; Gildea, R. J.; Howard, J. A. K.; Puschmann, H. OLEX2: A Complete Structure Solution, Refinement and Analysis Program. *J. Appl. Cryst*. **2009**, 42, 339-341.
9. Macrae, C. F.; Edgington, P. R.; McCabe, P.; Pidcock, E.; Shields, G. P.; Taylor, R.; Towler, M.; Streek, J. Mercury: visualization and analysis of crystal structures. *J. Appl. Cryst*. **2006**, 39, 343-350
10. Llunell, M.; Casanova, D.; Cirera, J.; Alemany, P.; Alvarez, S. SHAPE, version 2.1; Universitat de Barcelona: Barcelona, Spain, **2013**.
11. Sieklucka, B. New Donor-Acceptor System Based on $[Pt(NH_3)_4]^{2+}$ and $[W(CN)_8]^{3-}$ Ions. *J. Chem. Soc. Dalton Trans.* **1997**, 869-872.
12. Bonadio, F.; Gross, M.; Stoeckli, H.; Decurtins, S. High-spin molecules: synthesis, X-ray characterization, and magnetic behavior of two new cyano-bridged $Ni^{II}_9Mo^{V}_6$ and $Ni^{II}_9W^{V}_6$ clusters with a $S = 12$ ground state. *Inorg. Chem*. **2002**, *41*, 5891-5896.
13. Chorazy, S.; Rezyński, M.; Podgajny, R.; Nogás, W.; Buda, S.; Rams, M.; Nitek, W.; Nowicka, B.; Mlynarski, J.; Ohkoshi, S.; Sieklucka, B. Implementation of Chirality into High-Spin Ferromagnetic $Co^{II}_9W^{V}_6$ and $Ni^{II}_9W^{V}_6$ Cyanido-Bridged Clusters. *Cryst. Growth Des*. **2015**, *15*, 3573-3581.
14. Nowicka, B.; Rams, M.; Stadnicka, K.; Sieklucka, B. Reversible Guest-Induced Magnetic and Structural Single-Crystal-to-Single-Crystal Transformation in Microporous Coordination Network $\{[Ni(cyclam)]_3[W(CN)_8]_2\}_n$ *Inorg. Chem.* **2007**, *46*, 8123-8125.
